# Supplementary material for: Urinary markers of oxidative stress respond to infection and late-life in wild chimpanzees
Source: PLoS One. 2020 Sep 11;15(9):e0238066. doi: 10.1371/journal.pone.0238066 (PMC7486137; doi:10.1371/journal.pone.0238066)
Supplement: S1 Table — (DOCX) [file pone.0238066.s001.docx]

**­­S1 Table. Sampling per biomarker before, during, and after respiratory epidemic.**

| **Biomarker** | **n**  **_individuals_** | **n**  **_samples_** | **Mean ± sd of samples per individual** | | | | | **Proportion subjects represented** | | |
| --- | --- | --- | --- | --- | --- | --- | --- | --- | --- | --- |
|  |  |  | Before | During | | After | Before | | During | After |
| 8-OHdG | 26 | 258 | 3.35 ± 1.81 | 4.19 ± 3.15 | 2.7 ± 1.89 | | | 1 | 1 | 0.88 |
| Isoprostanes | 18 | 133 | 2.59 ± 1.8 | 3.75 ± 2.46 | 1.93 ± 1.03 | | | 0.94 | 0.89 | 0.83 |
| MDA- TBARS | 22 | 191 | 2.95 ± 1.79 | 4.1 ± 3.43 | 2.88 ± 2.06 | | | 0.91 | 0.95 | 0.73 |
| Neopterin | 25 | 229 | 2.88 ± 1.59 | 4.04 ± 3.12 | 2.55 ± 1.9 | | | 1 | 1 | 0.88 |
| TAC | 20 | 172 | 2.9 ± 1.52 | 4.11 ± 2.54 | 2.11 ± 0.99 | | | 1 | 0.9 | 0.95 |
