# Supplementary material for: Urinary markers of oxidative stress respond to infection and late-life in wild chimpanzees
Source: PLoS One. 2020 Sep 11;15(9):e0238066. doi: 10.1371/journal.pone.0238066 (PMC7486137; doi:10.1371/journal.pone.0238066)
Supplement: S2 Table — (DOCX) [file pone.0238066.s002.docx]

**S2 Table. Individual sampling per biomarker before, during, and after severe injury.**

| **ChimpID** | **Biomarker** | **Period of Injury** | | |
| --- | --- | --- | --- | --- |
|  |  | **Before** | **During** | **After** |
| BT | 8-OHdG | 7 | 9 | 3 |
|  | Isoprostanes | 6 | 8 | 3 |
|  | MDA- TBARS | 7 | 9 | 2 |
|  | Neopterin | 6 | 8 | 3 |
|  | TAC | 6 | 9 | 3 |
| GG | 8-OHdG | 4 | 5 | 3 |
|  | Isoprostanes | 3 | 4 | 2 |
|  | MDA- TBARS | 2 | 4 | 3 |
|  | Neopterin | 2 | 5 | 1 |
|  | TAC | 3 | 4 | 3 |
| PB | 8-OHdG | 8 | 4 | 7 |
|  | Isoprostanes | 6 | 5 | 4 |
|  | MDA- TBARS | 8 | 4 | 7 |
|  | Neopterin | 7 | 5 | 4 |
|  | TAC | 5 | 5 | 4 |

BT snare: Before 6/29/2016 – 7/25/16, During 9/4/16 –12/1/2016, After 1/5/17 – 2/26/17

GG snare: Before 1/9/2016 – 5/6/2016, During 5/23/16 –6/24/2016, After 11/29/16 – 1/26/17

PB combat wounds: Before 7/5/2012 – 11/6/2012, During 11/14/2012 –12/3/2012, After 12/28/12 – 2/1/13
