# Supplementary material for: Urinary markers of oxidative stress respond to infection and late-life in wild chimpanzees
Source: PLoS One. 2020 Sep 11;15(9):e0238066. doi: 10.1371/journal.pone.0238066 (PMC7486137; doi:10.1371/journal.pone.0238066)
Supplement: S3 Table. Individual sampling ranges A) per year and B) per biomarker per year leading up to death — (DOCX) [file pone.0238066.s003.docx]

**S3 Table. Individual sampling ranges per year for lead up to death validation.**

| ChimpID |  | sex | avg age | 2008 | 2009 | 2010 | 2011 | 2012 | 2013 | 2014 | 2015 | 2016 | 2017 |
| --- | --- | --- | --- | --- | --- | --- | --- | --- | --- | --- | --- | --- | --- |
| BL |  | F | 51.7 | 5 | 7 | 7 | 3 | 1 | 7 | 6 | 5 | 3 | - |
| KK |  | M | 26.5 | 0 | 7 | 1 | 2 | 5 | 15 | - | - | - | - |
| OU |  | F | 33.6 | 0 | 2 | 12 | 3 | 2 | 8 | 12 | 11 | 5 | 10 |
| ST |  | M | 55.6 | 5 | 3 | 0 | 6 | 4 | 4 | - | - | - | - |

* Death dates: BL 6/4/2016, KK 5/24/2013, OU 7/21/2017, ST 6/1/2013
