# Supplementary material for: Urinary markers of oxidative stress respond to infection and late-life in wild chimpanzees
Source: PLoS One. 2020 Sep 11;15(9):e0238066. doi: 10.1371/journal.pone.0238066 (PMC7486137; doi:10.1371/journal.pone.0238066)
Supplement: S4 Table — Models include only samples from individuals not during or after epidemic or injury. Significant relationships in bold. (DOCX) [file pone.0238066.s004.docx]

**S4 Table. Effect of time of day on biomarkers.** Models include only samples from individuals not during or after epidemic or injury. Significant relationships in bold.

| **Biomarker** | **n**  **_individuals_** | **n**  **_samples_** | **Predictor** | **Beta** | **SE** | **95% CI** | **p** |
| --- | --- | --- | --- | --- | --- | --- | --- |
| 8-OHdG | 33 | 490 | Intercept | 2.59 | 0.14 | 2.31 - 2.86 | <0.0001 |
|  |  |  | Hour | 0.02 | 0.01 | -0.01 - 0.04 | 0.162 |
| Isoprostanes | 20 | 95 | Intercept | 1.73 | 0.28 | 1.19 - 2.28 | <0.0001 |
|  |  |  | Hour | -0.04 | 0.03 | -0.09 - 0.01 | 0.143 |
| MDA-TBARS | 28 | 197 | Intercept | 1.48 | 0.18 | 1.14 - 1.83 | <0.0001 |
|  |  |  | Hour | 0.07 | 0.02 | 0.04 - 0.1 | **<0.0001** |
| Neopterin | 33 | 324 | Intercept | 7.28 | 0.12 | 7.05 - 7.51 | <0.0001 |
|  |  |  | Hour | -0.04 | 0.01 | -0.07 - -0.02 | **<0.0001** |
| TAC | 28 | 207 | Intercept | 1.91 | 0.13 | 1.65 - 2.18 | <0.0001 |
|  |  |  | Hour | -0.03 | 0.01 | -0.05 - 0 | **0.033** |
