# Supplementary material for: Urinary markers of oxidative stress respond to infection and late-life in wild chimpanzees
Source: PLoS One. 2020 Sep 11;15(9):e0238066. doi: 10.1371/journal.pone.0238066 (PMC7486137; doi:10.1371/journal.pone.0238066)
Supplement: S5 Table. Correlations between OS biomarkers, assessed with generalized linear mixed effects models with individual ID as a random effect. Significant relationships in bold — (DOCX) [file pone.0238066.s005.docx]

**S5 Table. Correlations between OS biomarkers**, assessed with generalized linear mixed effects models with individual ID as a random effect. Significant relationships in bold.

| **Biomarker 1** | **Biomarker 2** | **n**  **_individuals_** | **n**  **_samples_** | **Rho** | **p** |
| --- | --- | --- | --- | --- | --- |
| **8-OHdG** | **Isop** | 26 | 336 | **0.29** | **<0.001** |
| **8-OHdG** | **MDA-TBARS** | 33 | 484 | **0.1** | **0.03** |
| **8-OHdG** | **Neo** | 37 | 631 | **0.09** | **0.03** |
| 8-OHdG | TAC | 32 | 470 | -0.05 | 0.3 |
| **Isop** | **MDA-TBARS** | 26 | 291 | **0.12** | **0.047** |
| Isop | Neo | 26 | 300 | 0.07 | 0.23 |
| **Isop** | **TAC** | 26 | 327 | **0.49** | **<0.001** |
| MDA-TBARS | Neo | 33 | 381 | 0.06 | 0.27 |
| MDA-TBARS | TAC | 26 | 288 | 0 | 0.98 |
| **Neo** | **TAC** | 32 | 441 | **0.13** | **0.01** |
