# Supplementary material for: Urinary markers of oxidative stress respond to infection and late-life in wild chimpanzees
Source: PLoS One. 2020 Sep 11;15(9):e0238066. doi: 10.1371/journal.pone.0238066 (PMC7486137; doi:10.1371/journal.pone.0238066)
Supplement: S6 Table — Betas and standard deviations of predictors from generalized linear mixed effects model and percentage of overall variance explained by individual ID as a random effect. Age by sex interactions in grey shading, extracted from separate model that included main effects of age and sex. Significant effects in bold. (DOCX) [file pone.0238066.s006.docx]

**S6 Table. Cross-sectional variation in OS biomarker by individual age and sex.** Betas and standard deviations of predictors from generalized linear mixed effects model and percentage of overall variance explained by individual ID as a random effect. Age by sex interactions in grey shading, extracted from separate model that included main effects of age and sex. Significant effects in bold.

| **OS biomarker** | **n**  **_individuals_** | **n**  **_samples_** | **predictor** | **Beta** | **SE** | **95% CI** | **p** | **% RE** |
| --- | --- | --- | --- | --- | --- | --- | --- | --- |
| 8-OHdG | 36 | 582 | Intercept | 2.82 | 0.06 | 2.71 - 2.94 | < 0.001 | 6.92 |
|  |  |  | sex (M) | -0.07 | 0.08 | -0.23 - 0.09 | 0.401 |  |
|  |  |  | **age** | **-0.1** | **0.05** | **-0.19 - -0.01** | **0.028** |  |
| 8-OHdG int |  |  | sex (M) : age | -0.06 | 0.08 | -0.22 - 0.09 | 0.42 | 6.46 |
| Isoprostanes | 23 | 182 | Intercept | 1.34 | 0.13 | 1.08 - 1.6 | < 0.001 | 18.46 |
|  |  |  | sex (M) | -0.05 | 0.19 | -0.43 - 0.33 | 0.793 |  |
|  |  |  | age | 0.05 | 0.1 | -0.14 - 0.24 | 0.606 |  |
| Iso int |  |  | sex (M) : age | 0.09 | 0.18 | -0.27 - 0.45 | 0.618 | 17.6 |
| MDA-TBARS | 31 | 249 | Intercept | 2.59 | 0.07 | 2.45 - 2.72 | < 0.001 | 6.53 |
|  |  |  | sex (M) | -0.04 | 0.09 | -0.21 - 0.14 | 0.692 |  |
|  |  |  | age | -0.01 | 0.04 | -0.09 - 0.07 | 0.829 |  |
| MDA-TBARS int |  |  | sex (M) : age | -0.03 | 0.08 | -0.19 - 0.14 | 0.734 | 6.53 |
| Neopterin | 36 | 409 | Intercept | 6.97 | 0.06 | 6.84 - 7.09 | < 0.001 | 8 |
|  |  |  | sex (M) | 0.05 | 0.09 | -0.13 - 0.23 | 0.562 |  |
|  |  |  | age | -0.01 | 0.05 | -0.1 - 0.08 | 0.831 |  |
| Neopterin int |  |  | sex (M) : age | -0.01 | 0.09 | -0.19 - 0.17 | 0.922 | 8 |
| TAC | 31 | 295 | Intercept | 0.1 | 0.31 | -0.5 - 0.7 | 0.741 | 14.97 |
|  |  |  | sex (M) | -0.32 | 0.47 | -1.24 - 0.6 | 0.504 |  |
|  |  |  | age | -0.02 | 0.24 | -0.48 - 0.44 | 0.927 |  |
| TAC int |  |  | sex (M) : age | 0.38 | 0.47 | -0.55 - 1.3 | 0.436 | 15.06 |
