# Supplementary material for: Urinary markers of oxidative stress respond to infection and late-life in wild chimpanzees
Source: PLoS One. 2020 Sep 11;15(9):e0238066. doi: 10.1371/journal.pone.0238066 (PMC7486137; doi:10.1371/journal.pone.0238066)
Supplement: S7 Table — Betas and standard deviations of predictors from generalized linear mixed effects model and percentage of overall variance explained by individual ID as a random effect. Age by sex interactions in grey shading, extracted from separate model that included main effects of age and sex. Significant effects in bold. (DOCX) [file pone.0238066.s007.docx]

**S7 Table. Cross-sectional variation in OS biomarker of past prime individuals (> 35 years old) by age and sex.** Betas and standard deviations of predictors from generalized linear mixed effects model and percentage of overall variance explained by individual ID as a random effect. Age by sex interactions in grey shading, extracted from separate model that included main effects of age and sex. Significant effects in bold.

| **OS biomarker** | **n**  **_individuals_** | **n**  **_samples_** | **predictor** | **Beta** | **SE** | **95% CI** | **p** | **% RE** |
| --- | --- | --- | --- | --- | --- | --- | --- | --- |
| 8-OHdG | 9 | 138 | Intercept | 2.78 | 0.1 | 2.59 - 2.97 | p < 0.001 | 2.88 |
|  |  |  | sex (M) | -0.13 | 0.12 | -0.38 - 0.11 | 0.284 |  |
|  |  |  | age | 0 | 0.09 | -0.19 - 0.18 | 0.972 |  |
| 8-OHdG int |  |  | sex (M) : age | -0.01 | 0.14 | -0.29 - 0.26 | 0.931 | 2.57 |
| Isoprostanes | 6 | 71 | Intercept | 1 | 0.28 | 0.45 - 1.55 | 0 | 21.02 |
|  |  |  | sex (M) | 0.42 | 0.4 | -0.36 - 1.19 | 0.291 |  |
|  |  |  | age | 0.13 | 0.2 | -0.26 - 0.53 | 0.502 |  |
| Iso int |  |  | **sex (M) : age** | **-0.55** | **0.15** | **-0.84 - -0.26** | **0** | 0 |
| MDA-TBARS | 9 | 74 | Intercept | 2.6 | 0.06 | 2.48 - 2.72 | p < 0.001 | 0 |
|  |  |  | sex (M) | -0.06 | 0.08 | -0.22 - 0.1 | 0.474 |  |
|  |  |  | **age** | **0.09** | **0.04** | **0.02 - 0.16** | **0.013** |  |
| MDA-TBARS int |  |  | sex (M) : age | 0.04 | 0.07 | -0.1 - 0.19 | 0.558 | 0 |
| Neopterin | 9 | 102 | Intercept | 7.05 | 0.14 | 6.77 - 7.33 | p < 0.001 | 4.57 |
|  |  |  | sex (M) | -0.1 | 0.2 | -0.49 - 0.3 | 0.63 |  |
|  |  |  | age | 0.03 | 0.12 | -0.2 - 0.27 | 0.775 |  |
| Neopterin int |  |  | sex (M) : age | 0.03 | 0.2 | -0.36 - 0.41 | 0.897 | 4.35 |
| TAC | 6 | 77 | Intercept | -0.58 | 0.66 | -1.86 - 0.71 | 0.458 | 17.73 |
|  |  |  | sex (M) | 0.5 | 1.04 | -1.54 - 2.53 | 0.659 |  |
|  |  |  | age | 0.14 | 0.48 | -0.8 - 1.08 | 0.785 |  |
| TAC int |  |  | **sex (M) : age** | **-1.96** | **0.64** | **-3.22 - -0.7** | **0.003** | 0 |
