# Supplementary material for: Urinary markers of oxidative stress respond to infection and late-life in wild chimpanzees
Source: PLoS One. 2020 Sep 11;15(9):e0238066. doi: 10.1371/journal.pone.0238066 (PMC7486137; doi:10.1371/journal.pone.0238066)
Supplement: S8 Table — Models A) including and B) excluding relatively young individual KK. Betas and standard deviations of predictors from linear (neopterin and TAC) and generalized linear mixed effects model and percentage of overall variance explained by individual ID as a random effect. Significant effects in bold. (DOCX) [file pone.0238066.s008.docx]

**S8 Table. Variation in OS biomarkers among subjects that died during observation over years leading up to death.** Models A) including and B) excluding relatively young individual KK**.** Betas and standard deviations of predictors from linear (neopterin and TAC) and generalized linear mixed effects model and percentage of overall variance explained by individual ID as a random effect. Significant effects in bold.

| **A) OS biomarker** | **N**  **_individuals_** | **N**  **_samples_** | | **predictor** | **Beta** | **SE** | **95% CI** | **p** | **% RE** |
| --- | --- | --- | --- | --- | --- | --- | --- | --- | --- |
| 8-OHdG | 4 | 158 | Intercept | | 2.74 | 0.07 | 2.59 - 2.88 | < 0.001 | 1.84 |
|  |  |  | **Years until death** | | **-0.11** | **0.04** | **-0.18 - -0.05** | **0.001** |  |
|  |  |  | Sex (M) | | 0.04 | 0.11 | -0.19 - 0.26 | 0.751 |  |
| Isoprostanes | 4 | 117 | Intercept | | 1.18 | 0.08 | 1.03 - 1.33 | < 0.001 | 1.17 |
|  |  |  | **Years until death** | | **0.1** | **0.05** | **0.01 - 0.2** | **0.03** |  |
|  |  |  | Sex (M) | | 0.05 | 0.14 | -0.22 - 0.32 | 0.721 |  |
| MDA - TBARS | 4 | 103 | Intercept | | 2.59 | 0.08 | 2.44 - 2.74 | 0 | 1.39 |
|  |  |  | Years until death | | 0.09 | 0.05 | -0.01 - 0.19 | 0.091^†^ |  |
|  |  |  | Sex (M) | | 0.03 | 0.12 | -0.21 - 0.27 | 0.797 |  |
| Neopterin | 4 | 122 | Intercept | | 49.89 | 60.73 | -69.14 - 168.91 | 0.623 | 0.71 |
|  |  |  | Years until death | | 92.73 | 50.05 | -5.36 - 190.82 | 0.067^†^ |  |
|  |  |  | Sex (M) | | -225.82 | 116.68 | -454.51 - 2.87 | 0.19 |  |
| TAC | 4 | 120 | Intercept | | -0.32 | 0.42 | -1.15 - 0.5 | 0.561 | 4.5 |
|  |  |  | Years until death | | 0.2 | 0.21 | -0.22 - 0.62 | 0.345 |  |
|  |  |  | Sex (M) | | -0.14 | 0.72 | -1.54 - 1.27 | 0.863 |  |
| **B) OS biomarker** | **N**  **_individuals_** | **N**  **_samples_** | | **predictor** | **Beta** | **SE** | **95% CI** | **p** | **% RE** |
| 8-OHdG | 3 | 128 | Intercept | | 2.76 | 0.08 | 2.6 - 2.93 | < 0.001 | 2.56 |
|  |  |  | **Years until death** | | **-0.09** | **0.04** | **-0.16 - -0.02** | **0.011** |  |
|  |  |  | Sex (M) | | 0 | 0.16 | -0.3 - 0.31 | 0.989 |  |
| Isop | 3 | 103 | Intercept | | 1.17 | 0.08 | 1.02 - 1.32 | < 0.001 | 1.53 |
|  |  |  | **Years until death** | | **0.09** | **0.05** | **0 - 0.18** | **0.046** |  |
|  |  |  | Sex (M) | | -0.02 | 0.17 | -0.34 - 0.31 | 0.919 |  |
| MDA - TBARS | 3 | 82 | Intercept | | 2.57 | 0.07 | 2.43 - 2.72 | 0 | 1.43 |
|  |  |  | Years until death | | 0.1 | 0.05 | -0.01 - 0.2 | 0.072^†^ |  |
|  |  |  | Sex (M) | | 0.1 | 0.14 | -0.16 - 0.37 | 0.445 |  |
| Neo | 3 | 104 | Intercept | | 32.49 | 46.98 | -59.58 - 124.57 | 0.627 | 0.27 |
|  |  |  | **Years until death** | | **113.59** | **43.28** | **28.76 - 198.43** | **0.01** |  |
|  |  |  | **Sex (M)** | | **-382.35** | **122.78** | **-622.99 - -141.71** | **0.031** |  |
| TAC | 3 | 106 | Intercept | | -0.37 | 0.39 | -1.13 - 0.39 | 0.523 | 3.46 |
|  |  |  | Years until death | | 0.14 | 0.22 | -0.29 - 0.58 | 0.517 |  |
|  |  |  | Sex (M) | | -0.72 | 0.86 | -2.4 - 0.96 | 0.472 |  |
