# Supplementary figures and images for: Urinary markers of oxidative stress respond to infection and late-life in wild chimpanzees
Source: PLoS One. 2020 Sep 11;15(9):e0238066. doi: 10.1371/journal.pone.0238066 (PMC7486137; doi:10.1371/journal.pone.0238066)

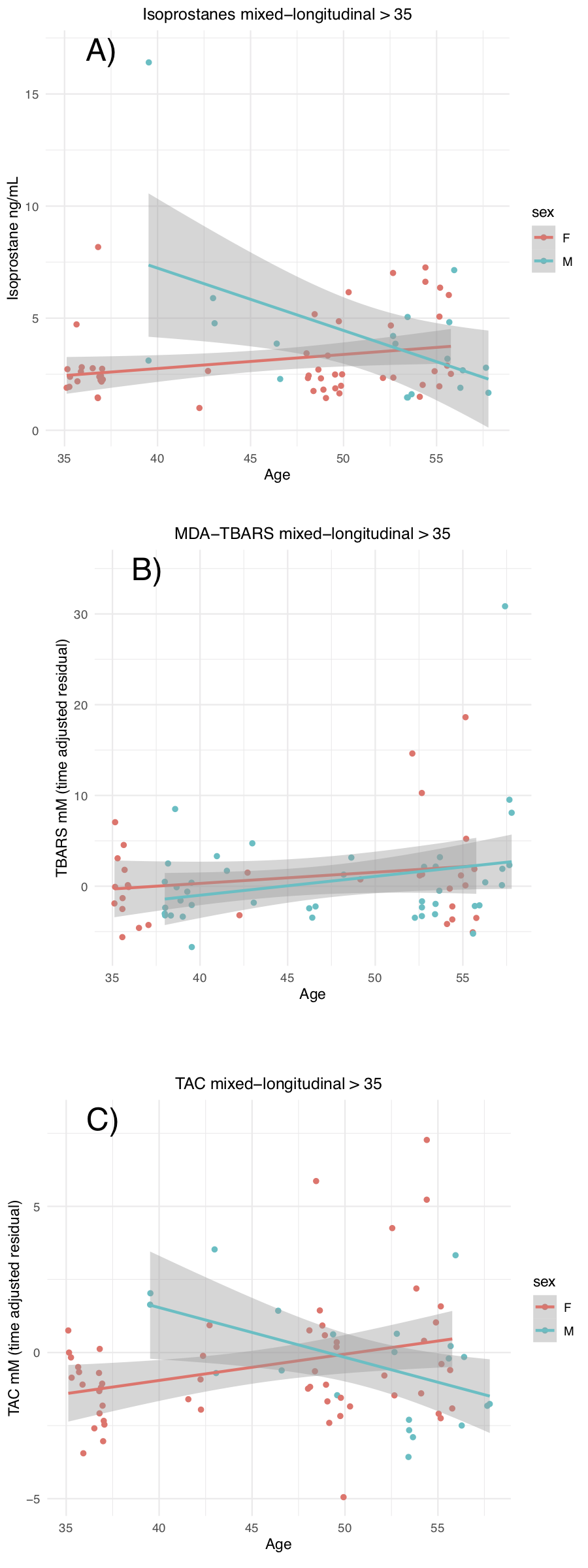

Supplement: S1 Fig — A) Isoprostanes increase with age among females and decrease among males (n = 6 individuals, 71 samples); B) MDA-TBARS increases with age (n = 9 individuals, 74 samples); C) TAC increases with age among females and decreases among males (n = 6 individuals, 77 samples). (TIFF) [file pone.0238066.s009.tiff]
