# Supplementary material for: Urinary markers of oxidative stress respond to infection and late-life in wild chimpanzees
Source: PLoS One. 2020 Sep 11;15(9):e0238066. doi: 10.1371/journal.pone.0238066 (PMC7486137; doi:10.1371/journal.pone.0238066)
Supplement: Attachment — Submitted filename: PONE-D-20-04637_Revised.pdf [file pone.0238066.s010.pdf]

PLOS ONE

Urinary markers of oxidative stress respond to infection and late-life in wild chimpanzees

--Manuscript Draft--

**Manuscript Number:**	PONE-D-20-04637
**Article Type:**	Research Article
**Full Title:**	Urinary markers of oxidative stress respond to infection and late-life in wild chimpanzees
**Short Title:**	Oxidative stress, infection, and aging in wild chimpanzees
**Corresponding Author:**	Nicole A. Thompson, Ph.D.<br>University of New Mexico<br>Albuquerque, NM UNITED STATES
**Keywords:**	aging, oxidative stress, primates, field work, biomarkers
**Abstract:**	Oxidative stress (OS) plays a central role in aging and results from a variety of stressors, making it a powerful measure of health and a way to examine costs associated with life history investments within and across species. However, few urinary OS markers have been examined under field conditions, particularly in primates, and their utility to non-invasively monitor the costs of acute stressors versus the long-term damage associated with aging is poorly understood. In this study, we examined variation in 5 urinary markers of oxidative damage and protection under 5 validation paradigms for 37 wild, chimpanzees living in the Kibale National Park, Uganda. We used 924 urine samples to examine responses to acute immune challenge (respiratory illness or severe wounding), as well as mixed-longitudinal and intra-individual variation with age. DNA damage (8-OHdG) correlated positively with all other markers of damage (F-isoprostanes, MDA-TBARS, and neopterin). Within individuals, all markers of damage responded to at least one if not both types of acute infection. While OS is expected to increase with age, this was not generally true in chimpanzees. However, significant changes in oxidative damage were detected within a few years of death among aged individuals. Our results indicate that OS can be measured using field-collected urine and integrates short- and long-term aspects of health. They further suggest that more data are needed from long-lived, wild animals to illuminate if common age-related increases in inflammation and OS damage are typical or recently aberrant in humans.
**Order of Authors:**	Nicole A. Thompson, Ph.D.
	Melissa Emery Thompson
	Martin A. Muller
	Zarin Machanda
	Emily Otali
	Richard Wrangham
**Opposed Reviewers:**	
**Additional Information:**	
**Question**	**Response**
**Financial Disclosure**<br><br>Enter a financial disclosure statement that describes the sources of funding for the work included in this submission. Review the submission guidelines for detailed requirements. View published research articles from *PLOS ONE* for specific	This work was funded by the National Institute of Aging and the NIH Office for Research on Women's Health award R01-049395 (MET, MM, ZM, EO, RW, https://www.nia.nih.gov/), and by the Leakey Foundation (https://leakeyfoundation.org/), the National Science Foundation (BCS-1355014, BCS-0849380, MET, MM, ZM, EO, RW, https://www.nsf.gov/), the University of New Mexico, and Harvard University. The funders had no role in study design, data collection and analysis, decision to publish, or preparation of the manuscript.

Powered by Editorial Manager® and ProduXion Manager® from Aries Systems Corporation

examples.<br><br>This statement is required for submission and **will appear in the published article** if the submission is accepted. Please make sure it is accurate.<br><br>**Unfunded studies**<br>Enter: *The author(s) received no specific funding for this work.*<br><br>**Funded studies**<br>Enter a statement with the following details:<br>• Initials of the authors who received each award<br>• Grant numbers awarded to each author<br>• The full name of each funder<br>• URL of each funder website<br>• Did the sponsors or funders play any role in the study design, data collection and analysis, decision to publish, or preparation of the manuscript?<br>• **NO** - Include this sentence at the end of your statement: *The funders had no role in study design, data collection and analysis, decision to publish, or preparation of the manuscript.*<br>• **YES** - Specify the role(s) played.<br><br>* typeset	
**Competing Interests**<br><br>Use the instructions below to enter a competing interest statement for this submission. On behalf of all authors, disclose any competing interests that could be perceived to bias this work—acknowledging all financial support and any other relevant financial or non-financial competing interests.<br><br>This statement **will appear in the published article** if the submission is accepted. Please make sure it is accurate. View published research articles from *PLOS ONE* for specific examples.	The authors have declared that no competing interests exist.

**NO authors have competing interests**<br><br>Enter: *The authors have declared that no competing interests exist.*<br><br>**Authors with competing interests**<br><br>Enter competing interest details beginning with this statement:<br><br>*I have read the journal's policy and the authors of this manuscript have the following competing interests: [insert competing interests here]*<br><br>* typeset	
**Ethics Statement**<br><br>Enter an ethics statement for this submission. This statement is required if the study involved:<br><br>• Human participants<br>• Human specimens or tissue<br>• Vertebrate animals or cephalopods<br>• Vertebrate embryos or tissues<br>• Field research<br><br>Write "N/A" if the submission does not require an ethics statement.<br><br>General guidance is provided below. Consult the submission guidelines for detailed instructions. **Make sure that all information entered here is included in the Methods section of the manuscript.**	The University of New Mexico IACUC approved of this study protocol 18-200739-MC. All study methods were non-invasive and observers maintain a distance of 5 m from chimpanzees at all times.

Powered by Editorial Manager® and ProduXion Manager® from Aries Systems Corporation

**Format for specific study types**<br><br>**Human Subject Research (involving human participants and/or tissue)**<br>• Give the name of the institutional review board or ethics committee that approved the study<br>• Include the approval number and/or a statement indicating approval of this research<br>• Indicate the form of consent obtained (written/oral) or the reason that consent was not obtained (e.g. the data were analyzed anonymously)<br><br>**Animal Research (involving vertebrate animals, embryos or tissues)**<br>• Provide the name of the Institutional Animal Care and Use Committee (IACUC) or other relevant ethics board that reviewed the study protocol, and indicate whether they approved this research or granted a formal waiver of ethical approval<br>• Include an approval number if one was obtained<br>• If the study involved *non-human primates*, add *additional details* about animal welfare and steps taken to ameliorate suffering<br>• If anesthesia, euthanasia, or any kind of animal sacrifice is part of the study, include briefly which substances and/or methods were applied<br><br>**Field Research**<br><br>Include the following details if this study involves the collection of plant, animal, or other materials from a natural setting:<br>• Field permit number<br>• Name of the institution or relevant body that granted permission	
**Data Availability**<br><br>Authors are required to make all data underlying the findings described fully available, without restriction, and from the time of publication. PLOS allows rare exceptions to address legal and ethical concerns. See the PLOS Data Policy and FAQ for detailed information.	Yes - all data are fully available without restriction

A Data Availability Statement describing where the data can be found is required at submission. Your answers to this question constitute the Data Availability Statement and **will be published in the article**, if accepted.<br><br>**Important:** Stating 'data available on request from the author' is not sufficient. If your data are only available upon request, select 'No' for the first question and explain your exceptional situation in the text box.<br><br>Do the authors confirm that all data underlying the findings described in their manuscript are fully available without restriction?	
**Describe where the data may be found in full sentences. If you are copying our sample text, replace any instances of XXX with the appropriate details.**<br><br>• If the data are **held or will be held in a public repository**, include URLs, accession numbers or DOIs. If this information will only be available after acceptance, indicate this by ticking the box below. For example: *All XXX files are available from the XXX database (accession number(s) XXX, XXX.*).<br>• If the data are all contained **within the manuscript and/or Supporting Information files**, enter the following: *All relevant data are within the manuscript and its Supporting Information files.*<br>• If neither of these applies but you are able to provide **details of access elsewhere**, with or without limitations, please do so. For example:<br><br>*Data cannot be shared publicly because of [XXX]. Data are available from the XXX Institutional Data Access / Ethics Committee (contact via XXX) for researchers who meet the criteria for access to confidential data.*<br><br>*The data underlying the results presented in the study are available from (include the name of the third party*	The data are currently available publicly on my GitHub page:<br>https://github.com/Gavago/Oxidative-stress-chimpanzee.git

and contact information or URL).<br>• This text is appropriate if the data are owned by a third party and authors do not have permission to share the data.<br><br>* typeset	
Additional data availability information:	

Powered by Editorial Manager® and ProduXion Manager® from Aries Systems Corporation

Dear Editors,

I am writing to submit our manuscript **"Urinary markers of oxidative stress respond to infection and late-life in wild chimpanzees"** for the exclusive consideration of PLOS ONE. My coauthors and I have no conflicts of interest to declare.

Background: For decades, oxidative stress has been a focal point of research on aging in humans and laboratory animals. More recently, it has been examined as a mechanism of trade-offs by which the wear-and-tear of life translates into biological decline in capture-sampled vertebrates. Markers of oxidative stress are commonly assayed in blood and sera, however many studies of wild animals are restricted to non-invasive sampling. As gerontology and evolutionary ecology begin to integrate, there is a need for reliable tools to evaluate oxidative damage and protection non-invasively in the wild, particularly as wild non-human primates and great apes provide valuable comparative data for human aging.

Our study: We performed a validation analysis of 5 urinary biomarkers of oxidative damage and protection in field collected urine from a community of wild chimpanzees in The Kibale National Forest, Uganda. We capitalized on the chimpanzee community's long-term health records, a rich collection of urine samples, and natural experiments to evaluate 1) the acute effects of infection and 2) the potential cumulative effect of age on urinary oxidative status. As a proof-of-concept, we evaluated acute responses in oxidative markers to a widespread respiratory epidemic and also to instances of severe open-wound injuries. We then further evaluated whether markers of damage increased with age in a mixed longitudinal sample and also within individuals approaching death.

Findings: We found that all markers of damage responded to at least one validation treatment, and that our marker of DNA damage positively correlated with all other damage markers, signaling its utility. Importantly, we find that different types of damage resulted from different kinds of infection (bacterial vs. viral) and in different time courses. This highlights the complexity and dynamism of oxidative status and the need for evaluating it with multiple key markers. Counter to predictions, we found no increase in oxidative damage with age in our mixed-longitudinal sample, though increased damage did appear in individuals as they approached death. Further, we found no overt signal of immunosenescence in chimpanzees, though again, an increase in lipid damage and inflammation among senescent individuals, suggesting that human-like patterns of inflammaging might occur in extreme cases.

Significance and novelty: Our study highlights markers of DNA damage, lipid peroxidation, and inflammation as useful tools to non-invasively monitor the oxidative status of wild primates. Our results further warrant future research on great apes and non-Western and post-industrialized human populations to evaluate whether inflammaging is a recent and atypical development in humans.

We expect this work to be of broad interest to researchers in disciplines including ecology, gerontology, and public health. We, the authors, thank you for your consideration of this work.

Sincerely,

Nicole A. Thompson, PhD
Department of Anthropology
University of New Mexico

Manuscript
Click here to download Manuscript OXS validation manuscript to submit no figures.docx

Urinary markers of oxidative stress respond to infection and late-life in wild chimpanzees

Nicole A. Thompson[1,2], Emily Otali[3], Zarin Machanda[4,3], Martin N. Muller[1,3], Richard Wrangham[5,3], Melissa Emery Thompson[1,3]

[1] University of New Mexico, Department of Anthropology, Albuquerque, NM, USA 87131

[2] University of New Mexico, Academic Science Education and Research Training program, Health Sciences Center, Albuquerque, NM, USA 87131

[3] Kibale Chimpanzee Project, Fort Portal, Uganda

[4] Tufts University, Department of Anthropology, Medford, MA, USA 02155

[5] Harvard University, Department of Human Evolutionary Biology, Cambridge, MA, USA 02138

Abstract

Oxidative stress (OS) plays a central role in aging and results from a variety of stressors, making it a powerful measure of health and a way to examine costs associated with life history investments within and across species. However, few urinary OS markers have been examined under field conditions, particularly in primates, and their utility to non-invasively monitor the costs of acute stressors versus the long-term damage associated with aging is poorly understood. In this study, we examined variation in 5 urinary markers of oxidative damage and protection under 5 validation paradigms for 37 wild, chimpanzees living in the Kibale National Park, Uganda. We used 924 urine samples to

examine responses to acute immune challenge (respiratory illness or severe wounding), as well as mixed-longitudinal and intra-individual variation with age. DNA damage (8-OHdG) correlated positively with all other markers of damage (F-isoprostanes, MDA-TBARS, and neopterin). Within individuals, all markers of damage responded to at least one if not both types of acute infection. While OS is expected to increase with age, this was not generally true in chimpanzees. However, significant changes in oxidative damage were detected within a few years of death among aged individuals. Our results indicate that OS can be measured using field-collected urine and integrates short- and long-term aspects of health. They further suggest that more data are needed from long-lived, wild animals to illuminate if common age-related increases in inflammation and OS damage are typical or recently aberrant in humans.

Introduction

As aerobic life forms use oxygen for energy metabolism, immune defense, and cell signaling, they are subject to cellular damage from oxygen's reactive species (Bartz & Piantadosi, 2010; Halliwell, 2006; Pham-Huy et al., 2008). The body balances reactive oxygen species with antioxidants and repair mechanisms, yet acute conditions can disrupt the balance to favor reactive oxidants, leading to a state of oxidative stress (OS, Finkel & Holbrook, 2000; Kregel & Zhang, 2007; Pizzino et al., 2017). As proximate damage accumulates, it can hinder physiological function, thus several theories place OS as a central mechanism of biological aging and age-related disease (Finkel & Holbrook, 2000; Kregel & Zhang, 2007; Pizzino et al., 2017). OS markers have been examined in relation to aging and challenges among animals living in the wild (particularly MDA-TBARS,

reactive oxygen metabolites, and various antioxidants, Metcalfe & Monaghan, 2013) and in the laboratory (Melov, 2002), usually in blood or other cells. However, several measures of OS are possible in urine, suggesting it may be feasible to develop a non-invasive toolkit to monitor OS in field settings where blood sampling is not possible. The objective of this paper is to examine a suite of urinary OS markers to determine their suitability for monitoring both acute, short term health challenges (e.g. infections and wounding) as well as long-term patterns of aging within a population of wild primates.

Organisms are expected to experience fundamental tradeoffs between life history functions such that, for example, energetic investments in immediate survival (immunity) and reproduction detract from longevity. Such marked energetic investments often result in a state of OS, which over a lifetime can lead to cumulative damage and functional loss, i.e. aging (Metcalfe & Alonso-Alvarez, 2010; Metcalfe & Monaghan, 2013; Selman et al., 2012; Soulsbury & Halsey, 2018; Speakman et al., 2015). Cumulative damage is particularly likely as endogenous antioxidants and the function of DNA repair mechanisms decline with age (J.-H. Chen et al., 2007; Fraga et al., 1990; Gorbunova et al., 2007; Hamilton et al., 2001; Kregel & Zhang, 2007; Maynard et al., 2015). For this reason, research has focused on oxidative stress as a mechanism of aging itself over half a century (Finkel & Holbrook, 2000; Harman, 1956; Sohal & Orr, 2012; Soulsbury & Halsey, 2018).

A central cause of oxidative damage and biological aging is the cellular or inflammatory immune response to infectious disease (Finkel & Holbrook, 2000; Pizzino et al., 2017). When exposed to a pathogen, the NADPH complex in phagocytes initiates the production of a cascade of reactive oxygen species such as superoxide and hydrogen

peroxide, and pro-oxidant cytokines such as TNF and IL-1 (Babior, 2000; Li et al., 2017; Thomas, 2017). Reactive oxidants damage pathogens but also cause collateral damage to self by oxidizing DNA, proteins, and lipids in tissues and in circulation (see Figure 1 in Selman et al., 2012). The accumulation of oxidative damage in cells and the independent decline in adaptive immunity with age can lead to the inflammatory response's common over-activation in old age (Daynes et al., 2003; Franceschi & Campisi, 2014; Martin-Montalvo et al., 2013). In humans, a chronic state of "inflammaging" is characterized by the self-perpetuating relationship of oxidative damage and inflammation in older individuals, and is involved in the pathogenesis of nearly all age-related disease including atherosclerosis, cancer, cataracts, and neurodegenerative disease (Franceschi et al., 2018; Franceschi & Campisi, 2014; Handa et al., 2017). However, recent studies indicate that the experience of chronic inflammation and its pathological consequences vary considerably across human populations (Gurven et al., 2008, 2009; Raichlen et al., 2017; Raichlen & Alexander, 2017), calling into question whether these diseases are natural and inevitable sequelae of human aging or consequences of modern environments. Evaluating the broader occurrence of chronic inflammation during aging among non-human primates could, therefore, illuminate the evolutionary roots of these common human ailments.

Markers of Oxidative Stress

While there is great interest in examining the role of redox status in health and longevity among wild primates, field researchers are currently in need of non-invasive methods to do so. Often, OS markers have been assayed in isolation, because they are simple to quantify (e.g. antioxidants) or inexpensive (e.g. MDA, Metcalfe & Monaghan,

2013). However, redox status involves a complex cascade of events, involving protection, damage, and repair of multiple target molecules at multiple time points and in multiple bodily regions (Lushchak, 2014; Pinchuk & Lichtenberg, 2014). Many sources therefore recommend measuring redox status via multiple markers that each provide an index of particular processes and responses (Belsky et al., 2015; Metcalfe & Monaghan, 2013; Speakman et al., 2015). In this study, we selected several candidate markers for which commercial urinary assays are available, that are known to be stable, and that have been in common use in clinical studies with evidence of response under stress conditions (e.g. disease).

Neopterin is a marker of the inflammatory immune response in primates and consequently an indicator of oxidative stress, as it is produced along with reactive oxygen species by activated macrophages (Murr et al., 2002). Its stability and response to viral and bacterial infection has been well-documented in laboratory (Denz et al., 1990; Higham et al., 2015), zoo (Behringer et al., 2017), and field-based studies of primates (D. F. Wu et al., 2018). Further, and consistent with patterns of immunosenescence and inflammaging, neopterin increases with age in humans (Murr et al., 2003) and Barbary macaques (Müller et al., 2017).

Products of oxidative damage to DNA, lipids, and proteins are also excreted in urine. Many such products have been validated as markers of acute stressors and are seen to correlate with aging in human and laboratory animal models (Il'yasova et al., 2012). 8-hydroxy-2'-deoxyguanosine (8-OHdG) is a product of the oxidation of the nucleotide guanine that is excreted in urine after base excision repair (Korkmaz et al., 2018). 8-OHdG increases in response to toxin exposures (CCl4, Kadiiska et al., 2005) and is

higher among individuals with vs. without various chronic diseases (Korkmaz et al., 2018; D. Wu et al., 2017). DNA damage increases with age in several critical tissues in both humans and rodents (Fraga et al., 1990; Frisard et al., 2007; Gan et al., 2012; Hamilton et al., 2001; Martin, 2008; Wolf et al., 2005).

Popular measures of lipid peroxidation, and corresponding damage to cellular membranes, include malondialdehyde (MDA) and, more recently, isoprostanes (Il'yasova et al., 2012). While MDA has been more commonly assayed across taxa due to its low cost and ease of measurement, it is nonspecific to lipid peroxidation and may conflate independent physiological processes (Il'yasova et al., 2012; Longmire et al., 1994). Isoprostanes, which are prostaglandin-like compounds that result from interaction of reactive oxygen species with arachidonic acid, appear more reliable and are growing in popularity (Il'yasova et al., 2009, 2010, 2012; Liu et al., 1999; Roberts & Morrow, 2000). Both markers increase in response to toxin exposure in rodents and are elevated in humans during various chronic diseases and smoking (Basu, 2008; Jain et al., 2002; Nowak et al., 1999; Tucker et al., 2013; Whiting et al., 2008, 2008). Humans and rodent models show increases in lipid peroxidation with age (MDA-TBARS, İnal et al., 2001; isoprostanes, Praticò, 2002); however, field studies in rhesus macaques and Soay sheep show no variation in MDA with age (Georgiev et al., 2015; Nussey et al., 2009).

OS can also be assessed via protein oxidation products, but these are less stable than other markers. To date, popular markers such as protein carbonyls and dityrosine are difficult to detect in urine in a consistent and reliable way (Cayman Chemical, 2016; Il'yasova et al., 2012; Kadiiska et al., 2005).

Protection from oxidative damage can also be measured non-invasively, as urine contains viable antioxidants from both endogenous and dietary origin. Total antioxidant capacity (TAC) is a measure of oxidative resistance indicated by the ability of a medium to prevent a redox reaction between controlled substances (Duplancic et al., 2011) and has been validated to negatively correlate with isoprostane levels (Lee et al., 2017). Endogenous antioxidants, in particular, often vary with age in a complex manner, as their basal expression increases but their inducibility of expression decreases with age in humans and rodents (Zhou et al., 2018).

Study system

We evaluated markers of OS in a mixed-longitudinal sample among wild chimpanzees in the Kanyawara community in the Kibale National Park, Uganda. Chimpanzees are long-lived (maximum lifespan > 60 years) and share a close evolutionary relationship with humans. Data from longevous animals are in need to clarify the role of OS in shaping life history trade-offs and aging (Belsky et al., 2015; Speakman et al., 2015), and data on chimpanzees provide a particularly essential comparison to evaluate evolutionary changes in the aging process during human evolution. While experimental studies of OS, with controlled exposure to stressors, have yielded important insights, naturalistic studies are necessary to examine the arc of senescence against the realistic evolutionary backdrop of limited resource abundance and heavy antigenic exposure (Fletcher et al., 2012; Metcalfe & Monaghan, 2013; Selman et al., 2012; Simm et al., 2008). Our study of the Kanyawara community also has the distinct advantage of dense, long-term urinary sampling, with daily observations of individual health status. This dataset provides an opportunity for analyzing responses to

natural experiments (e.g. infection, injury) and aging within individuals, and so reduces the potential confound of mortality selection to which cross-sectional designs are prone (Georgiev et al., 2015; Moffitt et al., 2017; Nussey et al., 2009; Selman et al., 2012; Speakman et al., 2015; Wrigley-Field, 2014).

This study aims to provide a biological validation of a panel of OS markers derived from field-collected urine. While products of OS are broadly applicable across animals, and urinary assays for OS are in common use for humans, we wanted to evaluate if these markers were sensitive enough that we could detect responses to major immunological challenges, even under conditions of opportunistic sample collection and under field collection and storage constraints. We also wanted to evaluate whether different markers yielded different information. Our biological validation had three facets. First, while the various markers we used reflect different aspects of redox status, we expected them to be moderately correlated with one another. Specifically, we expected markers associated with infection (neopterin) and oxidative damage (8-OHdG, F2-isoprostanes, MDA-TBARS) to be positively correlated with one another, and negatively correlated with antioxidant capacity (TAC). Second, we expected that these markers should exhibit a significant acute response to active immunological challenges: an epidemic of respiratory illness and cases of severe wounding. We expected levels of neopterin and markers of oxidative damage to increase in response to these challenges, and for TAC to decrease as antioxidant defenses were exhausted. These tests served as a proof of concept that these markers could reliably detect acute insults, and further to discriminate whether the different challenges (wounds vs. respiratory viral infection) yielded different oxidative responses. Our third major goal was to extend these findings

to test the hypothesis that the cumulative immunological burden of living under pathogen exposure leads to an increase in oxidative damage with age. Here, we examined the correlation of OS markers with age using a mixed-longitudinal sample comprised of repeated samples of 36 adult individuals. Because this approach introduces the possibility of mortality selection, we additionally looked at how OS markers changed in the several years leading up to death in a more densely sampled longitudinal dataset from 4 individuals. We expected markers of oxidative damage to increase, and antioxidant capacity to decrease, both as chimpanzees aged and as the time to death decreased.

Methods

Study site and data collection

Data were collected between 2009 and 2017 on the Kanyawara community of wild, unprovisioned chimpanzees in Kibale National Forest, Uganda. Ages of most subjects born after 1987 were known to within one month based on long-term demographic records collected during near-daily observations. Female chimpanzees often exhibit primary dispersal at sexual maturity, and thus new nulliparous females were assigned an age at immigration of 13 years, based upon the average age of females who have dispersed from the community. Ages of older individuals, most of whom were first identified in 1983, were estimated based on body size, if immature at first sighting, or on signs of relative age, including condition of body and hair and the presence of dependent offspring (see Hill et al., 2001; Muller & Wrangham, 2014).

Trained staff of the Kibale Chimpanzee Project recorded daily clinical signs of all observed individuals, including coughing, sneezing, wounds, limping, and skin conditions (see Emery Thompson et al., 2018). Coughs were further noted as dry or wet/productive, while wound documentation included descriptions of location, severity, and healing process. Observers collected urine samples opportunistically from identified individuals, using a disposable pipette to transfer urine from a plastic bag or leaf into 2.0 ml tubes. Urine samples had minimal dirt contamination and no contamination with feces, were stored immediately on ice, and were frozen within 0 – 14 hrs. OS marker concentrations exist in a more stable state in urine than in other media (Il'yasova et al., 2012), and most have been shown to vary minimally after remaining at room temperature upon collection for up to 16 – 24 hours (urinary neopterin, Heistermann & Higham, 2015; plasma TAC, Koracevic et al., 2001; urinary MDA, Lee & Kang, 2008; urinary 8-OHdG, Matsumoto et al., 2017). While no formal study has assessed the stability of urinary isoprostanes upon collection, concentrations in thawed urine samples decrease <15% after storage in the dark at room temperature for 2 days (Yan et al., 2007). Chimpanzee samples were transported frozen and on ice to the Comparative Human and Primate Physiology Center at UNM and stored at < -30°C until further analysis. Using linear regression, we found a weak negative relationship between storage time and MDA-TBARS concentration (ß = -0.3, $p = 0.046$) but none in any other biomarker ($p > 0.05$). We therefore reran age-related analyses of MDA-TBARS using samples stored for ≤ 3 years total (mixed-longitudinal), and with a difference in storage time ≤ 3 years (longitudinal, lead up to death) and found no change to our original results.

Assays for biomarkers of oxidative stress

Samples were assayed for 5 different urinary OS biomarkers using kits designed for urine. We used ELISAs for 8-hydroxy-2'-deoxyguanosine (**8-OHdG**, Catalog No. KOG-200S/E Genox JaICA), 15-Isoprostane F_{2t} (**isoprostanes**, Prod. No. E85 Oxford Biomedical Research), and **neopterin (**Ref. RE59321, IBL International), and colorimetric assays for malondialdehyde as thiobarbituric acid reactive substances (**MDA-TBARS**, Item No. 700870 Cayman Chemical) and total antioxidant capacity (**TAC**, $ABTS^+$ method, Item No. 709001 Cayman Chemical). In all cases, the chimpanzee samples yielded results with the suggested protocols and dilutions. We measured specific gravity of all samples with an Atago™ handheld refractometer (PAL-10S) and standardized biomarker concentrations for specific gravity following Miller et al. (2004). These corrections can lead to conspicuous outliers in very dilute samples. After visually inspecting the data inflated biomarker values at low dilutions, we omitted isoprostanes, neopterin, and TAC values for samples with SG < 1.004. Average recovery of enzyme immunoassays on spiked urine samples was 116% for 8-OHdG (88 – 137%) and 114% for isoprostanes (106 – 123%). Behringer et al. (2017) report 100.3% recovery of neopterin from spiked chimpanzee urine samples. Intra-assay CVs were 5.9% for 8-OHdG, 5.8% for isoprostanes, 3.1% for MDA-TBARS, 5.9% for neopterin, and 2.8% for TAC. Inter-assay CVs for high and low controls were 12.2% and 13.6% for 8-OHdG, 10.1% and 14.2% for isoprostanes, 3.7% and 6.7% for MDA-TBARS, 7.9% and 10.3% for neopterin, and 11.7% and 13.6% for TAC.

We aimed to select samples that had never previously been thawed, but this was not always possible as samples are periodically used for other laboratory studies. Sample

values were highly correlated after first and second thaws within all assays (range Spearman $r_S = 0.94 – 0.99$). We examined changes in biomarker concentrations between thaws by calculating the average proportional change in values between first and second thaws and performing a 2-tailed Wilcoxon rank sum test for significant differences: 131% for 8-OHdG (N = 36, W = 504, $p = 0.11$), 116% for isoprostanes (N = 36, W = 549, $p = 0.27$), 86.7% for MDA-TBARS (N = 37, W = 792, $p = 0.26$), 99.8% for neopterin (N = 30, W = 468, $p = 0.80$), and 130% for TAC (N = 35, W = 285, $p < 0.001$). We therefore aimed to assay samples on their first thaw for 8-OHdG, isoprostanes, and TAC. To increase sample size for underrepresented individuals, we assayed some samples that were previously thawed once (1-4 hours). The total proportion of samples assayed after their first thaw was 89% for 8-OHdG, 67% for isoprostanes, and 78% for TAC. MDA-TBARS and neopterin were assayed on either the first thaw or the second, after having been previously thawed for <1 hr. We were unable to assay all samples for all markers due to small sample volumes, thus the sample sizes vary by marker.

Validation approach, subjects, and sample selection

We conducted 4 different types of validation analyses to examine the relationship of oxidative stress biomarkers with aging, infection, and injury, as below. We assayed samples of individuals ≥ 10 years old, except in one case study of a 6 yr old male (BT) that experienced a serious injury.

1) <u>Before, during, and after respiratory epidemic:</u> Viral respiratory infections reliably induce macrophage responses and oxidative stress in humans (Li et al.,

2017; Schwarz, 1996). A severe respiratory epidemic broke out in the Kanyawara community in 2013, during which 87% of chimpanzees were observed to have wet, productive coughs. Respiratory epidemics have been a major source of mortality at Kanyawara, and in the 2013 outbreak, traced to human rhinovirus C, five individuals died (~ 9%, Emery Thompson et al., 2018; Scully et al., 2018). We used 265 samples from 27 individuals (12 M, 15 F) to examine within-individual changes in OS biomarkers between periods before, during, and after the 2013 epidemic. For "during" the epidemic, we chose samples from the 4-month period when the weekly proportion of community members showing signs of coughing and sneezing were elevated (date ranges in Table 1). This period was bookended by severe peaks in respiratory signs. We then identified 7-month periods "before" and "after" the prolonged outbreak. Given a single week when several members showed severe respiratory signs 2 months prior to the prolonged outbreak, we excluded these 2 months and restricted our control sample to the window 2-7 months before the outbreak. All individuals were sampled for at least two periods for any given biomarker. For specific sampling per biomarker, see Table S1.

2) Before, during, and after serious injury: As a further proof-of-concept that acute challenges lead to OS, we examined OS biomarker responses to traumatic injury in case studies of three individuals. We selected cases that were severe enough that they posed a strong risk of bacterial infection. Two chimpanzees, GG (14 yo F) and BT (6.3 yo M), were caught by wire hunting snares, which pose significant

risk of infection because the wire embeds in the flesh (King'ori, Obanda, Ndambiri, Runo, & Chiyo, 2018). While veterinary intervention is sometimes possible, it was not in these cases, thus neither individual received any antibiotics or other treatment. For each individual, the injuries resulted in the loss of 4 fingers. The third case, PB (18 yo M), was attacked and severely wounded by chimpanzees from a neighboring community (such attacks are often fatal). He received bite and/or tear wounds on both thighs, chest, face, testes, anus, heel, and hand; these resulted in significantly impaired movement and took >2 weeks to heal. All subjects were sampled over the 3 months before the injury, during the period between injury and healing (by visual assessment for bleeding, inflammation, infection, and scabbing), and for 3 months after the injury healed. For sampling date ranges by individual and biomarker, see Table S2.

3) Quarterly mixed-longitudinal: To assess natural variation by age and sex in OS biomarkers, we assayed 594 samples from 36 chimpanzees (18 F, 18 M) aged 10+ years over a period of approximately 5 years (range 0.75 – 7.8 yrs/individual) for an average of 16.5 ± 10.2 samples/individual. We attempted to represent subjects consistently over time by selecting approximately one sample in each quarter-year. Mixed-longitudinal samples did not include samples collected during and after the respiratory epidemic or severe injury.

4) Lead up to death: We additionally conducted more intensive longitudinal sampling of 4 individuals who died during the study period. Three were among the oldest chimpanzees known from Kanyawara: OU (F age 48, cause unknown), ST (M age 58, respiratory illness), and BL (F age 56 yrs, cause unknown) while

the fourth died suddenly in his prime (KK, M age 28, respiratory illness plus physical trauma). We sampled each individual throughout the 6 to 9 consecutive years preceding death, for an average of 5.0 ± 3.6 samples per individual-year (total N = 161 samples). Because KK's relative young age at death, we ran statistical models both including and excluding him. For sampling date ranges per individual and sample numbers per year see Table S3.

Statistical analysis

Oxidative stress varies throughout the day, as a result of the metabolic costs of daily activities and circadian rhythms in antioxidant production (Hardeland et al., 2003; Kanabrocki et al., 2002). We attempted to minimize time of day effects by prioritizing samples taken in the morning hours; however, this was not always possible. We therefore assessed the relationship between time of sample collection and biomarker concentrations using a generalized linear mixed model (GLMM, in R package "lme4") with a gamma error distribution and log-link function. Models included biomarker concentration as a response, hour of sample collection as a fixed effect, and individual as a random intercept with hour of collection as a random slope. To minimize the influence of potential outliers, these models were applied to samples collected during baseline conditions, i.e. not collected during or after a respiratory epidemic or injury, and not from the four individuals whose deaths were the focus of our analyses above. Because MDA-TBARS, neopterin, and TAC varied significantly with time of day (Table S4), we thereafter expressed their concentrations as time corrected values, calculated as residuals from predicted values, calculated using the population slope and intercept of baseline models.

As individuals were occasionally sampled more than once on a given day, our final dataset used average daily values of either unadjusted biomarker concentrations (8-OHdG and isoprostanes) or their time-adjusted residuals (MDA-TBARS, neopterin, and TAC). All Ns reported reflect the statistical sample size = days of sampling.

We constructed linear and generalized linear mixed models (LMMs and GLMMs) to test the predictions of our validation hypotheses. For all models, we assessed the appropriateness of generalized error distributions using the “descdist” function (R package “fitdistrplus”) prior to modeling and visually inspecting quantile-quantile plots of model residuals. To assess correlation between markers, we examined pairs of biomarker concentrations as fixed effect and response, with a random effect of individual. In validation models assessing OS marker variation by infection and age, we included subject age and sex as fixed effects with individual as a random intercept. In modeling biomarker responses to a respiratory epidemic or injury, we included time period with levels of before, during, and after as a fixed effect. In mixed-longitudinal analyses, we considered an age-by-sex interaction term to assess whether biomarkers varied with individual age differently by sex. While it may in the future be desirable to collapse biomarker values into fewer dimensions or a single index, this was not practical in the given dataset because not all samples had sufficient volume for all markers, and, as our study evolved, newly-added isoprostanes were assessed in different samples to aim for urine not previously thawed. Thus, too few samples contained the full complement for dimension reduction, and we chose to focus on the individual markers in this study.

Results

i. Correlations between OS biomarkers

Isoprostanes, MDA-TBARS, and neopterin all positively correlated with 8-OHdG (Figure 1, Table S5). The strongest correlation of these was between isoprostanes and 8-OHdG, markers of lipid and DNA damage, respectively. Isoprostanes and MDA-TBARS, both measures of lipid peroxidation, positively correlated with one another, however weakly (Spearman's correlation, $r_s = 0.12$, $p = 0.047$, 2-tailed). TAC positively correlated with both neopterin and isoprostanes, and did not correlate with 8-OHdG or MDA-TBARS, counter to predictions that TAC would negatively correlate with markers of oxidative damage.

Figure 1. Correlations between OS biomarkers. Spearman's rho written in heat tiles, with significant correlations indicated as **** p < 0.0001, *** p < 0.001, ** p < 0.01, * p < 0.05, and † p < 0.10.

Validation 1. Before, during, and after respiratory epidemic

Several OS biomarkers changed significantly during and/or after the respiratory outbreak, though not always in the ways predicted. Only 8-OHdG was elevated during the epidemic period compared with before (Table 1, Figure 2A), and levels of 8-OHdG remained elevated after the epidemic was over. MDA-TBARS (Figure 2C) and neopterin (Figure 2D) did not increase during the epidemic itself, but were increased in the recovery period after the epidemic. Neither isoprostanes nor TAC varied over the course of the epidemic (Figure 2B & 2E). Notably, several samples from the 4 adults who died during the epidemic (1 young infant also died and was not sampled) were high for 8-

OHdG, MDA-TBARS, or neopterin before the period when respiratory signs were observed.

Figure 2. Variation in concentrations of A) 8-OHdG, B) F-Isoprostanes, C) MDA-TBARS, D) neopterin, and E) Total antioxidant capacity before, during, and after a severe respiratory epidemic. Points are individual sample-day concentrations. Black diamonds are sample-days from any of the 5 individuals that died during the epidemic. Boxes represent median and interquartile ranges of concentrations. Horizontal dashed lines indicate significant contrasts in biomarker concentrations between periods determined by generalized linear mixed effects models. For N individuals and samples per biomarker see Table S1.

Table 1. Variation in OS biomarkers between periods of before, during, and after a severe respiratory epidemic. Betas and standard deviations of contrasts from generalized linear mixed effects model with Betas of significant contrasts in bold.

OS Biomarker	N individuals	N samples	Before vs. During	Before vs. After	During vs. After	Sex (M)	Age
8-OHdG	26	258	**0.15 ± 0.06 ***	**0.23 ± 0.07 *****	0.07 ± 0.07	-0.1 ± 0.08	0.01 ± 0.04
Isoprostanes	18	133	*-0.2 ± 0.11 †*	-0.08 ± 0.13	0.11 ± 0.13	-0.21 ± 0.21	-0.04 ± 0.09
MDA-TBARS	22	191	*-1.66 ± 0.94 †*	0.91 ± 1.08	**2.57 ± 1.01 ***	-0.37 ± 1.25	**1.45 ± 0.56 ***
Neopterin	25	229	0.16 ± 0.1	**0.54 ± 0.11 *****	**0.37 ± 0.11 *****	0.01 ± 0.11	0.03 ± 0.05
TAC	20	172	*-0.89 ± 0.46 †*	0.06 ± 0.53	*0.95 ± 0.52 †*	-0.35 ± 0.62	-0.14 ± 0.28

† $p < 0.10$, * $p < 0.05$, ** $p < 0.01$, *** $p < 0.001$
Sample date ranges for Before: 10/01/2012 – 2/28/2013; During: 5/3/2013 – 8/28/2013; After: 9/11/2013 – 3/29/2014.

Validation 2. Before, during, and after severe injury

Open wounds provide a different kind of infection risk than respiratory viruses, and accordingly the responses of OS biomarkers were expected to differ. Both isoprostanes and neopterin increased during injury relative to pre-injury levels (Figure 3B & 3D;

Table 2). The three subjects showed no significant variation in 8-OHdG, MDA-TBARS, or TAC levels in response to injury (Figure 3A, 3C, 3E).

Oneindividual (GG, snared, Figure 3) exhibited an unusual pattern for all of the biomarkers examined. While GG's isoprostanes increased markedly in response to injury, unlike the other individuals, they continued to increase after the wound was visibly healed. On the other hand, while her levels of neopterin were similarly high during the injury phase as the other two, and her post-healing levels were similarly low, she had already exhibited highly elevated neopterin and MDA-TBARS, as well as 8-OHdG prior to her injury. It is possible that GG was suffering an unknown condition despite having exhibited no other visible clinical signs over the pre-injury period.

Figure 3. Variation in OS biomarkers according to stage of severe injury. B) Isoprostanes and D) neopterin varied significantly according to periods of before, during, and after severe injury. *Asterisks indicate significant contrasts between periods, as determined by glmer models. For N individuals and samples see Table S2.

Table 2. Variation in OS biomarkers between periods of before, during, and after severe injury. Betas and standard deviations of contrasts from generalized linear mixed effects model with Betas of significant contrasts in bold. N = 3 individuals (2 M, 1 F).

OS Biomarker	N samples	Before vs. During	Before vs. After	During vs. After	Sex (M)	Age
8-OHdG	50	*0.29 ± 0.17 †*	-0.05 ± 0.18	*-0.34 ± 0.19 †*	**-0.85 ± 0.17 *** **	0.23 ± 0.19
Isoprostanes	41	**0.59 ± 0.16 *** **	0.28 ± 0.19	-0.31 ± 0.19	*-0.34 ± 0.18 †*	**-0.38 ± 0.18 * **
MDA-TBARS	46	-0.23 ± 0.18	-0.25 ± 0.21	-0.02 ± 0.21	0.11 ± 0.2	0.05 ± 0.21
Neopterin	41	**0.8 ± 0.18 *** **	-0.03 ± 0.23	**-0.82 ± 0.23 *** **	0.26 ± 0.21	0.34 ± 0.2
TAC	42	0.11 ± 0.13	0.04 ± 0.15	-0.07 ± 0.14	**0.53 ± 0.13 *** **	**-0.31 ± 0.14 * **

† p < 0.10, * p < 0.05, ** p < 0.01, *** p < 0.001. Sampling date ranges per individual in Table S2.

Validation 3. Mixed-longitudinal analysis

Within a mixed-longitudinal sample of individuals, 8-OHdG decreased weakly but significantly with age (GLMM; $N_{individuals, samples}$ = 36, 583; ß ± se = -0.1 ± 0.05, 95% CI = -0.2 – -0.01, p = 0.03). These effects were not significantly different for males and females. No other markers (isoprostanes, MDA-TBARS, neopterin, TAC) varied with age, sex, or age moderated by sex in the mixed-longitudinal sample (Table S6).

Figure 4. Decrease in 8-OHdG with age in a mixed-longitudinal sample (N = 36 individuals, 583 samples).

Validation 4. Lead up to death

For individuals who died during the study period, isoprostanes increased as time to death decreased (Figure 5B, Table S7A, glmer ß = 0.1, 95% CI 0.01 - 0.2, $p = 0.03$). 8-OHdG decreased as individuals approached death (Figure 5A, Table S7A, glmer ß = -0.11, 95% CI -0.18 - -0.05, $p = 0.001$). Neither neopterin, MDA-TBARS, nor TAC exhibited a significant change over time (Figure 5C-5E). However, inspection of the data suggested that the individual who died in its prime (KK, 27.9 years at death) sometimes exhibited a different pattern than the other 3, who died at an advanced age. Indeed, in models that included only past-prime individuals (OU, BL, ST), neopterin increased as individuals approached death (Figure 5C, Table S7B, lmer ß = 113.6, 95% CI 28.8 – 198.4, $p = 0.01$). The exclusion of the young subject did not affect our results for the other markers (Table S7A,B).

Figure 5. Variation in OS biomarkers by year before individuals' death. *A) 8-OHdG decreased and B) Isoprostanes increased among the 4 subjects as they approach death. D) Neopterin increased among past prime individuals as they approached death. Solid line drawn with simple linear regression, unlike mixed effect models that determined significance. Black dotted line represents population median in biomarker from samples unrelated to epidemic, injury or eventual death.

Discussion

In this study, we examined a set of urinary biomarkers to determine their utility for assessing oxidative stress in future studies of health and aging in wild chimpanzees (Table 5). Redox dynamics in the body are highly complex and, accordingly, our results were not always straightforward. We found that DNA damage (urinary 8-OHdG) correlated with all other measures of oxidative damage (isoprostanes, MDA-TBARS) and

inflammatory activity (neopterin), and each damage marker responded significantly to one or both natural experiments in which chimpanzees faced significant acute immunological challenges. This suggests overall that urinary markers can provide a useful signal of intracellular oxidative damage induced during immunological challenges in a natural environment. Our present study was powered to detect medium to large effect sizes in its various analyses; we encourage investigators to carefully consult Brysbaert & Stevens (2018) in designing future studies with repeated sampling per subject.

Our results further indicate that different markers may be affected by different types of stressors and along different time courses. Specifically, although both respiratory and open-wound infections produced inflammation, the respiratory virus primarily led to prolonged DNA damage, while open wounds led to acute increases in lipid peroxidation (isoprostanes). Though the precise mechanisms are unclear, different pathogens lead to different types of damage depending on the immune pathways activated and the cellular sites affected (Imlay, 2003; Zinman et al., 2011). Such findings lead us to recommend that using multiple biomarkers is the most prudent approach to evaluate oxidative damage, particularly if research involves health monitoring that is not specific to one particular type of stressor.

Finally, while theory posits that oxidative stress should increase rather steadily with age, we did not find this. None of the biomarkers increased with age in the mixed-longitudinal sample. Increasing lipid peroxidation (isoprostanes) and inflammatory activity (neopterin) were observed among elderly chimpanzees in the years preceding death. One marker, DNA damage (urinary 8-OHdG) actually declined with age. These findings suggest that while oxidative stress may play a role in declining health during

senescence, wild chimpanzees do not experience increases in oxidative damage throughout adulthood, as some humans populations do (Capuron et al., 2014; Dorjgochoo et al., 2012; Martínez de Toda et al., 2019; Matsumoto et al., 2017). Such a lack of global increases in OS with age aligns with limited findings in other wild animals (Georgiev et al., 2015; Nussey et al., 2009; Selman et al., 2012).

Table 5. Summary of findings.

Marker	*Respiratory infection*	*Open wound injury*	*Age*	*Years before death*
8-OHdG	↑ During & After	-	↓	↓
Isop	-	↑ During	-	↑
MDA-TBARS	↑ After	-	-	-
Neopterin	↑ After	↑ During	-	↑*
TAC	-	-	-	-

*In model excluding KK

General correlations among OS biomarkers

Damage to DNA (8-OHdG) positively correlated with lipid peroxidation (isoprostanes, MDA-TBARS) and macrophage activity (neopterin). This result was expected because reactive oxygen species, including hypochlorous acid, peroxynitrite, and the hydroxyl radical, cause damage to both DNA and lipids and are produced in large quantities by activated phagocytic cells, such as macrophages (Babior, 2000; Finkel & Holbrook, 2000; Pizzino et al., 2017). Surprisingly, however, neither isoprostanes nor MDA-TBARS correlated with neopterin. Generally, the modest associations across markers suggest that they each provide independent information on oxidative status. As

our experiments suggest, this may be due to variable pathways of oxidative damage (Imlay, 2003) or to differences in time scales of response.

We predicted that total antioxidant capacity (TAC) would be negatively associated with markers of damage because it is this imbalance that characterizes oxidative “stress". As damage-causing radicals increase, more antioxidants should be depleted, as during activities such as exercise (Balakrishnan & Anuradha, 1998). In fact, however, we found that TAC correlated positively with lipid damage (isoprostanes) and inflammation (neopterin). One reason that TAC may increase alongside markers of damage is that pro-oxidants activate Nrf2 transcription factor, which increases the expression of endogenous antioxidant enzymes (Q. M. Chen & Maltagliati, 2018; Hybertson et al., 2011). Nrf2 has evolved such that common processes that produce reactive oxygen species, e.g. physical activity and critical illness, also adaptively induce production of antioxidants such as glutathione and uric acid (Ghiselli et al., 2001; MacKinnon et al., 1999; Maxwell et al., 1993; Sahlin et al., 1991). Overall, we found little evidence to suggest that antioxidant capacity was limiting in chimpanzees, either during acute stressors or during aging.

Acute responses to infection

Infection from both respiratory disease and severe injury led to changes in neopterin and either in markers of DNA damage (8-OHdG) or in lipid peroxidation (isoprostanes). Reactive oxygen species are generated in large numbers during the oxidative burst of macrophages (Babior, 2000; Thomas, 2017). Accordingly, changes in

neopterin levels paralleled those of the main OS marker that responded to each type of infection, respiratory (8-OHdG) and open wounds (isoprostanes).

In other non-human primates assayed during infections, neopterin concentrations are clearly elevated relative to baseline, both in captive (Behringer et al., 2017; Higham et al., 2015) and wild animals (D. F. Wu et al., 2018), and often peak during a short window (Heistermann & Higham, 2015). For example, among chimpanzees in the Taï National Park, Cote D'Ivoire, neopterin was elevated during an acute viral respiratory outbreak and declined to baseline levels after signs of respiratory infection diminished (D. F. Wu et al., 2018). Similarly, in rhesus macaques experimentally inoculated with SIV, concentrations in neopterin peaked sharply over 2-3 days approximately 2 weeks following infection, after which concentrations declined but remained elevated from baseline for several weeks (Heistermann & Higham, 2015). The fact that neopterin appears to spike briefly, even when the infectious agent persists, means that it can be difficult to capture this signal. At Kanyawara, we did not observe a significant increase in neopterin during the course of a viral respiratory outbreak, despite relatively high sampling intensity. However, we did observe increased levels in the period after the respiratory outbreak. By contrast, neopterin levels increased acutely when chimpanzees had severe open wounds, a time of potentially high infection risk, and returned to levels that were close to baseline after injuries were healed.

Unlike the short-term and acute respiratory infections monitored in Taï chimpanzees and rhesus macaques, the respiratory epidemic in the Kanyawara chimpanzee community lasted for 4 months, with three waves of respiratory signs suggesting possible reinfection (Scully et al., 2018). Indeed, 47 of 53 community

members were seen coughing and 31 had productive coughs during multiple months of 2013, and deaths occurred over a span of three months. While it is surprising that our opportunistic sampling procedure failed to capture the expected spikes in neopterin during the outbreak, the elevation in levels after the outbreak suggest that such prolonged (or repeated) illness exerts somatic costs that far outlive the active infection. Whereas abrupt increases in neopterin can be useful to identify infections (in cases that might not be as obvious as respiratory illnesses), it is these lasting inflammatory effects, related to increased oxidative damage, that are predicted to be of particular relevance to longevity. Accordingly, 8-OHdG was increased in our chimpanzees not only during but after the respiratory outbreak.

Variation in damage with age and senescence

In a mixed-longitudinal sample, we found no evidence for increases in oxidative damage and inflammatory activity, nor a decrease in antioxidant capacity with chronological age. In humans, such changes with age often appear in cross-sectional and mixed-longitudinal studies (Bektas et al., 2017; Dorjgochoo et al., 2012; Gan et al., 2018; İnal et al., 2001; Martínez de Toda et al., 2019; Praticò, 2002; Sekhar et al., 2018), but not always (Feillet-Coudray et al., 1999; Frisard et al., 2007; Ialongo, 2017; Nakano et al., 2013; Pilger et al., 2000). For example, urinary 8-OHdG increased with age in all patients with muscular dystrophy (Matsumoto et al., 2017) and between certain age categories in a large sample of men and women (Gan et al., 2018). However, it did not increase with age in silica miners, and instead was simply higher in miners with vs. without silicosis (Pilger et al., 2000).

In contrast to our mixed-longitudinal analysis, isoprostanes and neopterin did increase among very old chimpanzees in their years prior to their deaths. A possible reason for the difference in results between our mixed-longitudinal and within-individual age-based analyses is selection bias, as chimpanzee populations have higher variation in mortality than human populations typically do (Muller & Wrangham, 2014; Wood et al., 2017; Wrigley-Field, 2014). For example, only 18% of chimpanzees at Kanyawara live until age 30, whereas 42% of Hadza hunter gatherers reach age 30 (Wood et al., 2017). Alternatively, while chimpanzees experience acute periods of oxidative stress across the life course they, like many humans, may not suffer a chronic and global redox imbalance unless they reach a very old age and/or are experiencing critical declines in health. In either case, we lack evidence that cumulative oxidative damage is a typical component of chimpanzee aging, despite long lifespans.

The signal of immunosenescence in chimpanzees, characterized by increased inflammation with age, appears weaker than it does in humans where immunosenescence is prominent in both longitudinal and cross-sectional studies. Indeed, in humans increased inflammatory activity is a core component of aging that is visible cross-sectionally in both post-industrial (Bektas et al., 2017; Daynes et al., 2003; Franceschi & Campisi, 2014; Fulop et al., 2018; Murr et al., 2003) and some indigenous horticultural populations (Gurven et al., 2008). The within-individual increase in lipid peroxidation and inflammation in past-prime chimpanzees preceding death could, however, suggest a late-emerging pattern of inflammaging. Namely, on top of an already immunosenescent profile, cellular debris from oxidative damage could perpetuate an inflammatory immune response (Bektas et al., 2017; Daynes et al., 2003).

An unexpected finding was a decrease in urinary 8-OHdG in association with senescence. This pattern was weakly expressed in the mixed-longitudinal data but emerged quite strongly in the years shortly before death. There are several plausible mechanisms to explain such a pattern. First, declining 8-OHdG could result from declining energy metabolism with age, e.g. decreased reproductive effort and reduced physical activity (Frisard et al., 2007; Georgiev et al., 2015; Nussey et al., 2011). For example, urinary 8-OHdG decreased with age in a sample of male rhesus macaques, which mirrored a decline in reproductive effort (Georgiev et al., 2015). However, contradictory evidence from human and rodent studies show that correlations of oxidative damage with RMR and reproductive investment are absent or negative, respectively, possibly because damage induces the expression of protective antioxidant enzymes (Frisard et al., 2007; Selman et al., 2012). A second reason for the age-related decline in 8-OHdG is perhaps because its excretion into urine is actually dependent on nucleotide excision, a key process involved in repairing DNA lesions (Cooke et al., 2005; Il'yasova et al., 2012; Korkmaz et al., 2018; Pilger et al., 2000). While assays of urinary 8-OHdG are commonly used, often under the assumption that repair processes are maximally efficient, nucleotide excision repair in fact declines with age in both humans and mice (Gorbunova et al., 2007; Maynard et al., 2015). Relatedly, studies in rats have found that while 8-OHdG in organs and blood cells increased with age, urinary 8-OHdG decreased (Fraga et al., 1990).

Limitations

We acknowledge certain limitations of this study. Our natural experiments were simply that and accordingly limited in scope. Such conditions were sufficient to evaluate responses to acute challenges, but a larger sample assayed over time is necessary to characterize normative changes in redox status with age. Non-invasive measures, too, are often limited in their specificity. Currently, for example, DNA damage and repair cannot be measured directly and independently of one another in urine (e.g., Nagel et al., 2014; Olive & Banáth, 2006). Also, general antioxidant capacity measures allow particularly abundant antioxidants, such as uric acid, to drown out nuanced signals in other antioxidants (Costantini, 2011; Duplancic et al., 2011; Ghiselli et al., 2001; Ialongo, 2017). Non-invasive markers should thus be interpreted as indicators rather than direct measures of physiological processes.

Conclusions

Urinary markers of DNA damage and lipid peroxidation, specifically, 8-hydroxy-2'-deoxyguanosine and 15-Isoprostane F_{2t}, are particularly useful, alongside inflammatory markers (neopterin), to monitor acute oxidative stress and its potential role in age-related declines in health and mortality in wild primates. We advise measuring both DNA and lipid markers simultaneously, as they may indicate different pathways of oxidative damage resulting from different pathogenic challenges and age.

Although a larger sample is required to authoritatively delineate age-related patterns of OS and immunosenescence in chimpanzees, we found no evidence that oxidative stress generally accumulates or that immune regulation generally declines with age in this species. Oxidative damage and inflammation do, however, appear to be

associated with healthspan, as they increase in particularly old individuals that are approaching death. Such findings broadly warrant further research on non-WEIRD humans and apes (Henrich et al., 2010) to identify if the common occurrence of inflammaging in humans is evolutionarily aberrant. Lastly, the limitation of urinary 8-OHdG as a marker of both DNA damage and repair emphasizes the value of both current invasive sampling to characterize age-related changes in DNA damage, and future development of assays for DNA repair processes in non-invasive samples.

Acknowledgements:

We thank the staff and field assistants of the Kibale Chimpanzee Project for collecting samples and daily records of health status. The authors also thank the Uganda Wildlife Association, the Uganda National Council for Science and Technology, and Makerere University Biological Field Station for their support in conducting long term research in Kibale National Park. Kris Sabbi, Seth Merkley, and Megan Cole provided assistance in the laboratory.

References:

Babior, B. M. (2000). Phagocytes and oxidative stress. *The American Journal of Medicine*, *109*(1), 33–44. https://doi.org/10.1016/S0002-9343(00)00481-2

Balakrishnan, S. D., & Anuradha, C. V. (1998). Exercise, depletion of antioxidants and antioxidant manipulation. *Cell Biochemistry and Function*, *16*(4), 269–275. https://doi.org/10.1002/(SICI)1099-0844(1998120)16:4<269::AID-CBF797>3.0.CO;2-B

Bartz, R. R., & Piantadosi, C. A. (2010). Clinical review: Oxygen as a signaling molecule. *Critical Care (London, England)*, *14*(5), 234–234. https://doi.org/10.1186/cc9185

Basu, S. (2008). F2-Isoprostanes in Human Health and Diseases: From Molecular Mechanisms to Clinical Implications. *Antioxidants & Redox Signaling*, *10*(8), 1405–1434. https://doi.org/10.1089/ars.2007.1956

Behringer, V., Stevens, J., Leendertz, F., Hohmann, G., & Deschner, T. (2017). Validation of a method for the assessment of urinary neopterin levels to monitor health status in non-human-primate species. *Frontiers in Physiology*, *8*(51). https://doi.org/10.3389/fphys.2017.00051

Bektas, A., Schurman, S. H., Sen, R., & Ferrucci, L. (2017). Human T cell immunosenescence and inflammation in aging. *Journal of Leukocyte Biology*, *102*(4), 977–988. https://doi.org/10.1189/jlb.3RI0716-335R

Belsky, D. W., Caspi, A., Houts, R., Cohen, H. J., Corcoran, D. L., Danese, A., Harrington, H., Israel, S., Levine, M. E., Schaefer, J. D., Sugden, K., Williams, B., Yashin, A. I., Poulton, R., & Moffitt, T. E. (2015). Quantification of biological aging in young adults. *Proceedings of the National Academy of Sciences*, *112*(30), E4104. https://doi.org/10.1073/pnas.1506264112

Brysbaert, M., & Stevens, M. (2018). Power Analysis and Effect Size in Mixed Effects Models: A Tutorial. *Journal of Cognition*, *1*(1), 9. https://doi.org/10.5334/joc.10

Capuron, L., Geisler, S., Kurz, K., Leblhuber, F., Sperner-Unterweger, B., & Fuchs, D. (2014). Activated Immune System and Inflammation in Healthy Ageing: Relevance for Tryptophan and Neopterin Metabolism. *Current Pharmaceutical Design*, *20*(38), 6048–6057.

Cayman Chemical. (2016). *Protein Carbonyl Colorimetric Assay Kit*. https://www.caymanchem.com/pdfs/10005020.pdf

Chen, J.-H., Hales, C. N., & Ozanne, S. E. (2007). DNA damage, cellular senescence and organismal ageing: Causal or correlative? *Nucleic Acids Research*, *35*(22), 7417–7428. https://doi.org/10.1093/nar/gkm681

Chen, Q. M., & Maltagliati, A. J. (2018). Nrf2 at the heart of oxidative stress and cardiac protection. *Physiological Genomics*, *50*(2), 77–97. https://doi.org/10.1152/physiolgenomics.00041.2017

Cooke, M. S., Evans, M. D., Dove, R., Rozalski, R., Gackowski, D., Siomek, A., Lunec, J., & Olinski, R. (2005). DNA repair is responsible for the presence of oxidatively damaged DNA lesions in urine. *Genes and Environment*, *574*(1), 58–66. https://doi.org/10.1016/j.mrfmmm.2005.01.022

Costantini, D. (2011). On the measurement of circulating antioxidant capacity and the nightmare of uric acid. *Methods in Ecology and Evolution*, *2*(3), 321–325. https://doi.org/doi:10.1111/j.2041-210X.2010.00080.x

Daynes, R. A., Enioutina, E. Y., & Jones, D. C. (2003). Role of Redox Imbalance in the Molecular Mechanisms Responsible for Immunosenescence. *Antioxidants & Redox Signaling*, *5*(5), 537–548. https://doi.org/10.1089/152308603770310185

Denz, H., Fuchs, D., Hausen, A., Huber, H., Nachbaur, D., Reibnegger, G., Thaler, J., Werner, E. R., & Wachter, H. (1990). Value of urinary neopterin in the differential diagnosis of bacterial and viral infections. *Klinische Wochenschrift*, *68*(4), 218–222. https://doi.org/10.1007/BF01662720

Dorjgochoo, T., Gao, Y.-T., Chow, W.-H., Shu, X., Yang, G., Cai, Q., Rothman, N., Cai, H., Li, H., Deng, X., Franke, A., Roberts, L. J., Milne, G., Zheng, W., & Dai, Q. (2012). Major metabolite of F2-isoprostane in urine may be a more sensitive biomarker of oxidative stress than isoprostane itself. *The American Journal of Clinical Nutrition*, *96*(2), 405–414. https://doi.org/10.3945/ajcn.112.034918

Duplancic, D., Kukoc-Modun, L., Modun, D., & Radic, N. (2011). Simple and Rapid Method for the Determination of Uric Acid-Independent Antioxidant Capacity. *Molecules*, *16*(8). https://doi.org/10.3390/molecules16087058

Emery Thompson, M., Machanda Zarin, P., Scully Erik, J., Enigk Drew, K., Otali, E., Muller Martin, N., Goldberg Tony, L., Chapman Colin, A., & Wrangham Richard, W. (2018). Risk factors for respiratory illness in a community of wild chimpanzees (Pan troglodytes schweinfurthii). *Royal Society Open Science*, *5*(9), 180840. https://doi.org/10.1098/rsos.180840

Feillet-Coudray, C., Tourtauchaux, R., Niculescu, M., Rock, E., Tauveron, I., Alexandre-Gouabau, M.-C., Rayssiguier, Y., Jalenques, I., & Mazur, A. (1999). Plasma levels of 8-epiPGF2α, an in vivo marker of oxidative stress, are not affected by aging or Alzheimer's disease. *Free Radical Biology and Medicine*, *27*(3), 463–469. https://doi.org/10.1016/S0891-5849(99)00096-9

Finkel, T., & Holbrook, N. J. (2000). Oxidants, oxidative stress and the biology of ageing. *Nature*, *408*(6809), 239–247.

Fletcher, Q. E., Selman, C., Boutin, S., McAdam, A. G., Woods, S. B., Seo, A. Y., Leeuwenburgh, C., Speakman, J. R., & Humphries, M. M. (2012). Oxidative damage increases with reproductive energy expenditure and is reduced by food-supplementation. *Evolution*, no-no. https://doi.org/10.1111/evo.12014

Fraga, C. G., Shigenaga, M. K., Park, J. W., Degan, P., & Ames, B. N. (1990). Oxidative damage to DNA during aging: 8-hydroxy-2'-deoxyguanosine in rat organ DNA and urine. *Proceedings of the National Academy of Sciences*, *87*(12), 4533. https://doi.org/10.1073/pnas.87.12.4533

Franceschi, C., & Campisi, J. (2014). Chronic Inflammation (Inflammaging) and Its Potential Contribution to Age-Associated Diseases. *The Journals of Gerontology: Series A*, *69*(Suppl_1), S4–S9. https://doi.org/10.1093/gerona/glu057

Franceschi, C., Garagnani, P., Parini, P., Giuliani, C., & Santoro, A. (2018). Inflammaging: A new immune–metabolic viewpoint for age-related diseases. *Nature Reviews Endocrinology*, *14*(10), 576–590. https://doi.org/10.1038/s41574-018-0059-4

Frisard, M. I., Broussard, A., Davies, S. S., Roberts, L. J., Rood, J., de Jonge, L., Fang, X., Jazwinski, S. M., Deutsch, W. A., & Ravussin, E. (2007). Aging, Resting Metabolic Rate, and Oxidative Damage: Results From the Louisiana Healthy Aging Study. *The Journals of Gerontology: Series A*, *62*(7), 752–759. https://doi.org/10.1093/gerona/62.7.752

Fulop, T., Larbi, A., Dupuis, G., Le Page, A., Frost, E. H., Cohen, A. A., Witkowski, J. M., & Franceschi, C. (2018). Immunosenescence and Inflamm-Aging As Two Sides of the Same Coin: Friends or Foes? *Frontiers in Immunology*, *8*. https://doi.org/10.3389/fimmu.2017.01960

Gan, W., Liu, X.-L., Yu, T., Zou, Y.-G., Li, T.-T., Wang, S., Deng, J., Wang, L.-L., & Cai, J.-P. (2018). Urinary 8-oxo-7,8-dihydroguanosine as a Potential Biomarker of Aging. *Frontiers in Aging Neuroscience*, *10*. https://doi.org/10.3389/fnagi.2018.00034

Gan, W., Nie, B., Shi, F., Xu, X.-M., Qian, J.-C., Takagi, Y., Hayakawa, H., Sekiguchi, M., & Cai, J.-P. (2012). Age-dependent increases in the oxidative damage of DNA, RNA, and their metabolites in normal and senescence-accelerated mice analyzed by LC–MS/MS: Urinary 8-oxoguanosine as a novel biomarker of aging. *Free Radical Biology and Medicine*, *52*(9), 1700–1707. https://doi.org/10.1016/j.freeradbiomed.2012.02.016

Georgiev, A. V., Thompson, M. E., Mandalaywala, T. M., & Maestripieri, D. (2015). Oxidative stress as an indicator of the costs of reproduction among free-ranging rhesus macaques. *The Journal of Experimental Biology*, *218*(13), 1981. https://doi.org/10.1242/jeb.121947

Ghiselli, A., Serafini, M., Natella, F., & Scaccini, C. (2001). Total antioxidant capacity as a tool to assess redox status: Critical view and experimental data. In *Bio-Assays for Oxidative Stress Status* (pp. 219–227). Elsevier.

Gorbunova, V., Seluanov, A., Mao, Z., & Hine, C. (2007). Changes in DNA repair during aging. *Nucleic Acids Research*, *35*(22), 7466–7474. https://doi.org/10.1093/nar/gkm756

Gurven, M., Kaplan, H., Winking, J., Eid Rodriguez, D., Vasunilashorn, S., Kim, J. K., Finch, C., & Crimmins, E. (2009). Inflammation and Infection Do Not Promote Arterial Aging and Cardiovascular Disease Risk Factors among Lean Horticulturalists. *PLOS ONE*, *4*(8), e6590. https://doi.org/10.1371/journal.pone.0006590

Gurven, M., Kaplan, H., Winking, J., Finch, C., & Crimmins, E. M. (2008). Aging and Inflammation in Two Epidemiological Worlds. *The Journals of Gerontology: Series A*, *63*(2), 196–199. https://doi.org/10.1093/gerona/63.2.196

Halliwell, B. (2006). Reactive Species and Antioxidants. Redox Biology Is a Fundamental Theme of Aerobic Life. *Plant Physiology*, *141*(2), 312. https://doi.org/10.1104/pp.106.077073

Hamilton, M. L., Van Remmen, H., Drake, J. A., Yang, H., Guo, Z. M., Kewitt, K., Walter, C. A., & Richardson, A. (2001). Does oxidative damage to DNA increase with age? *Proceedings of the National Academy of Sciences*, *98*(18), 10469. https://doi.org/10.1073/pnas.171202698

Handa, J. T., Cano, M., Wang, L., Datta, S., & Liu, T. (2017). Lipids, oxidized lipids, oxidation-specific epitopes, and Age-related Macular Degeneration. *Biochimica et Biophysica Acta. Molecular and Cell Biology of Lipids*, *1862*(4), 430–440. https://doi.org/10.1016/j.bbalip.2016.07.013

Hardeland, R., Coto- Montes, A., & Poeggeler, B. (2003). Circadian Rhythms, Oxidative Stress, and Antioxidative Defense Mechanisms. *Chronobiology International*, *20*(6), 921–962. https://doi.org/10.1081/CBI-120025245

Harman, D. (1956). Aging: A theory based on free radical and radiation chemistry. *Journal of Gerontology*, *11*, 298–300.

Heistermann, M., & Higham, J. P. (2015). Urinary neopterin, a non-invasive marker of mammalian cellular actiation, is highly stable under field conditions. *Scientific Reports*. https://doi.org/10.1038/srep16308

Henrich, J., Heine, S. J., & Norenzayan, A. (2010). The weirdest people in the world? *Behavioral and Brain Sciences*, *33*(2–3), 61–83. https://doi.org/10.1017/S0140525X0999152X

Higham, J. P., Kraus, C., Stahl-Hennig, C., Engelhardt, A., Fuchs, D., & Heistermann, M. (2015). Evaluating noninvasive markers of nonhuman primate immune activation and inflammation. *American Journal of Physical Anthropology*, *158*(4), 673–684. https://doi.org/10.1002/ajpa.22821

Hill, K., Boesch, C., Goodall, J., Pusey, A., Williams, J., & Wrangham, R. (2001). Mortality rates among wild chimpanzees. *Journal of Human Evolution*, *40*(5), 437–450. https://doi.org/10.1006/jhev.2001.0469

Hybertson, B. M., Gao, B., Bose, S. K., & McCord, J. M. (2011). Oxidative stress in health and disease: The therapeutic potential of Nrf2 activation. *Molecular Aspects of Medicine*, *32*(4), 234–246. https://doi.org/10.1016/j.mam.2011.10.006

Ialongo, C. (2017). Preanalytic of total antioxidant capacity assays performed in serum, plasma, urine and saliva. *Clinical Biochemistry*, *50*(6), 356–363. https://doi.org/10.1016/j.clinbiochem.2016.11.037

Il'yasova, D., Mixon, G., Wang, F., Marcom, P. K., Marks, J., Spasojevich, I., Craft, N., Arredondo, F., & DiGiulio, R. (2009). Markers of oxidative status in a clinical model of oxidative assault: A pilot study in human blood following doxorubicin administration. *Biomarkers*, *14*(5), 321–325. https://doi.org/10.1080/13547500902946757

Il'yasova, D., Scarbrough, P., & Spasojevic, I. (2012). Urinary biomarkers of oxidative status. *Clinica Chimica Acta*, *413*(19), 1446–1453. https://doi.org/10.1016/j.cca.2012.06.012

Il'yasova, D., Spasojevic, I., Wang, F., Tolun, A. A., Base, K., Young, S. P., Marcom, P. K., Marks, J., Mixon, G., DiGiulio, R., & Millington, D. S. (2010). Urinary biomarkers of oxidative status in a clinical model of oxidative assault. *Cancer Epidemiology Biomarkers & Prevention*, *19*(6), 1506. https://doi.org/10.1158/1055-9965.EPI-10-0211

Imlay, J. A. (2003). Pathways of Oxidative Damage. *Annual Review of Microbiology*, *57*(1), 395–418. https://doi.org/10.1146/annurev.micro.57.030502.090938

İnal, M. E., Kanbak, G., & Sunal, E. (2001). Antioxidant enzyme activities and malondialdehyde levels related to aging. *Clinica Chimica Acta*, *305*(1), 75–80. https://doi.org/10.1016/S0009-8981(00)00422-8

Jain, S. K., Pemberton, P. W., Smith, A., McMahon, R. F. T., Burrows, P. C., Aboutwerat, A., & Warnes, T. W. (2002). Oxidative stress in chronic hepatitis C: not just a feature of late stage disease. *Journal of Hepatology*, *36*(6), 805–811. https://doi.org/10.1016/S0168-8278(02)00060-0

Kadiiska, M. B., Gladen, B. C., Baird, D. D., Germolec, D., Graham, L. B., Parker, C. E., Nyska, A., Wachsman, J. T., Ames, B. N., Basu, S., Brot, N., FitzGerald, G. A., Floyd, R. A., George, M., Heinecke, J. W., Hatch, G. E., Hensley, K., Lawson, J. A., Marnett, L. J., … Barrett, J. C. (2005). Biomarkers of Oxidative Stress Study II: Are oxidation products of lipids, proteins, and DNA markers of CCl4 poisoning? *Free Radical Biology and Medicine*, *38*(6), 698–710. https://doi.org/10.1016/j.freeradbiomed.2004.09.017

Kanabrocki, E. L., Murray, D., Hermida, R. C., Scott, G. S., Bremner, W. F., Ryan, M. D., Ayala, D. E., Third, J. L. H. C., Shirazi, P., Nemchausky, B. A., & Hooper, D. C. (2002). Circadian variation in oxidative stress markers in healthy and type II diabetic men. *Chronobiology International*, *19*(2), 423–439. https://doi.org/10.1081/CBI-120002914

Koracevic, D., Koracevic, G., Djordjevic, V., Andrejevic, S., & Cosic, V. (2001). Method for the measurement of antioxidant activity in human fluids. *Journal of Clinical Pathology*, *54*(5), 356. https://doi.org/10.1136/jcp.54.5.356

Korkmaz, K. S., Butuner, B. D., & Roggenbuck, D. (2018). Detection of 8-OHdG as a diagnostic biomarker. *Journal of Laboratory and Precision Medicine; November 2018*.

Kregel, K. C., & Zhang, H. J. (2007). An integrated view of oxidative stress in aging: Basic mechanisms, functional effects, and pathological considerations. *American Journal of Physiology-Regulatory, Integrative and Comparative Physiology*, *292*(1), R18–R36. https://doi.org/10.1152/ajpregu.00327.2006

Lee, & Kang, D. (2008). Stability and Intra-Individual Variation of Urinary Malondialdehyde and 2-Naphthol. *Journal of Preventative Medicine and Public Health*, *41*(3), 195–199. https://doi.org/2008.41.3.195

Lee, S. G., Wang, T., Vance, T. M., Hubert, P., Kim, D.-O., Koo, S. I., & Chun, O. K. (2017). Validation of Analytical Methods for Plasma Total Antioxidant Capacity by Comparing with Urinary 8-Isoprostane Level. *Journal of Microbiology and Biotechnology*, *27*(2), 388–394. https://doi.org/10.4014/jmb.1604.04053

Li, Z., Xu, X., Leng, X., He, M., Wang, J., Cheng, S., & Wu, H. (2017). Roles of reactive oxygen species in cell signaling pathways and immune responses to viral infections. *Archives of Virology*, *162*(3), 603–610. https://doi.org/10.1007/s00705-016-3130-2

Liu, T., Stern, A., Roberts, L. J., & Morrow, J. D. (1999). The Isoprostanes: Novel Prostaglandin-Like Products of the Free Radical-Catalyzed Peroxidation of Arachidonic Acid. *Journal of Biomedical Science*, *6*(4), 226–235. https://doi.org/10.1159/000025391

Longmire, A. W., Swift, L. L., Jackson Roberts, L., Awad, J. A., Burk, R. F., & Morrow, J. D. (1994). Effect of oxygen tension on the generation of F2-isoprostanes and malondialdehyde in peroxidizing rat liver microsomes. *Biochemical Pharmacology*, *47*(7), 1173–1177. https://doi.org/10.1016/0006-2952(94)90389-1

Lushchak, V. I. (2014). Free radicals, reactive oxygen species, oxidative stress and its classification. *Chemico-Biological Interactions*, *224*, 164–175. https://doi.org/10.1016/j.cbi.2014.10.016

MacKinnon, K. L., Molnar, Z., Lowe, D., Watson, I. D., & Shearer, E. (1999). Measures of total free radical activity in critically ill patients. *Clinical Biochemistry*, *32*(4), 263–268. https://doi.org/10.1016/S0009-9120(98)00109-X

Martin, L. J. (2008). DNA Damage and Repair: Relevance to Mechanisms of Neurodegeneration. *Journal of Neuropathology & Experimental Neurology*, *67*(5), 377–387. https://doi.org/10.1097/NEN.0b013e31816ff780

Martínez de Toda, I., Vida, C., Garrido, A., & De la Fuente, M. (2019). Redox Parameters as Markers of the Rate of Aging and Predictors of Life Span. *The Journals of Gerontology: Series A*. https://doi.org/10.1093/gerona/glz033

Martin-Montalvo, A., Mercken, E. M., Mitchell, S. J., Palacios, H. H., Mote, P. L., Scheibye-Knudsen, M., Gomes, A. P., Ward, T. M., Minor, R. K., Blouin, M.-J., Schwab, M., Pollak, M., Zhang, Y., Yu, Y., Becker, K. G., Bohr, V. A., Ingram, D. K., Sinclair, D. A., Wolf, N. S., … de Cabo, R. (2013). Metformin improves healthspan and lifespan in mice. *Nature Communications*, *4*, 2192. https://doi.org/10.1038/ncomms3192 https://www.nature.com/articles/ncomms3192#supplementary-information

Matsumoto, M., Awano, H., Nagai, M., Shirakawa, T., Iijima, K., & Matsuo, M. (2017). Urinary excretion of 8-OHdG, a biomarker of oxidative DNA damage, increases with age in DMD patients. *Neuromuscular Disorders*, *27*, S168–S169. https://doi.org/10.1016/j.nmd.2017.06.274

Maxwell, S. R., Jakeman, P., Thomason, H., Leguen, C., & Thorpe, G. H. (1993). Changes in plasma antioxidant status during eccentric exercise and the effect of vitamin supplementation. *Free Radic Res Commun*, *19*(3), 191–202.

Maynard, S., Fang, E. F., Scheibye-Knudsen, M., Croteau, D. L., & Bohr, V. A. (2015). DNA Damage, DNA Repair, Aging, and Neurodegeneration. *Cold Spring Harbor Perspectives in Medicine*, *5*(10), a025130. https://doi.org/10.1101/cshperspect.a025130

Melov, S. (2002). Animal models of oxidative stress, aging, and therapeutic antioxidant interventions. *The International Journal of Biochemistry & Cell Biology*, *34*(11), 1395–1400. https://doi.org/10.1016/s1357-2725(02)00086-9

Metcalfe, N. B., & Alonso-Alvarez, C. (2010). Oxidative stress as a life-history constraint: The role of reactive oxygen species in shaping phenotypes from conception to death. *Functional Ecology*, *24*(5), 984–996. https://doi.org/doi:10.1111/j.1365-2435.2010.01750.x

Metcalfe, N. B., & Monaghan, P. (2013). Does reproduction cause oxidative stress? An open question. *Trends in Ecology & Evolution*, *28*(6), 347–350. https://doi.org/10.1016/j.tree.2013.01.015

Miller, R. C., Brindle, E., Holman, D. J., Shofer, J., Klein, N. A., Soules, M. R., & O'Connor, K. A. (2004). Comparison of specific gravity and creatinine for normalizing urinary reproductive hormone concentrations. *Clinical Chemistry*, *50*(5), 924.

Moffitt, T. E., Belsky, D. W., Danese, A., Poulton, R., & Caspi, A. (2017). The Longitudinal study of aging in human young adults: Knowledge gaps and research agenda. *The Journals of Gerontology: Series A*, *72*(2), 210–215. https://doi.org/10.1093/gerona/glw191

Muller, M. N., & Wrangham, R. W. (2014). Mortality rates among Kanyawara chimpanzees. *Journal of Human Evolution*, *66*, 107–114. https://doi.org/10.1016/j.jhevol.2013.10.004

Müller, N., Heistermann, M., Strube, C., Schülke, O., & Ostner, J. (2017). Age, but not anthelmintic treatment, is associated with urinary neopterin levels in semi-free ranging Barbary macaques. *Scientific Reports*, *7*, 41973. https://doi.org/10.1038/srep41973

Murr, C., Hainz, U., Asch, E., Berger, P., Jenewein, B., Saurwein-Teissl, M., Grubeck-Loebenstein, B., & Fuchs, D. (2003). Association of increased neopterin production with decreased humoral immunity in the elderly. *Experimental Gerontology*, *38*(5), 583–587. https://doi.org/10.1016/S0531-5565(03)00062-7

Murr, C., Widner, B., & Fuchs, B. W. and D. (2002). Neopterin as a Marker for Immune System Activation. *Current Drug Metabolism*, *3*(2), 175–187. https://doi.org/10.2174/1389200024605082

Nagel, Z. D., Margulies, C. M., Chaim, I. A., McRee, S. K., Mazzucato, P., Ahmad, A., Abo, R. P., Butty, V. L., Forget, A. L., & Samson, L. D. (2014). Multiplexed DNA repair assays for multiple lesions and multiple doses via transcription inhibition and transcriptional mutagenesis. *Proceedings of the National Academy of Sciences*, *111*(18), E1823. https://doi.org/10.1073/pnas.1401182111

Nakano, R., Nakagawa, R., Tokimoto, N., & Okanoya, K. (2013). Alarm call discrimination in a social rodent: Adult but not juvenile degu calls induce high vigilance. *Journal of Ethology*, 1–7.

Nowak, D., Kasielski, M., Antczak, A., Pietras, T., & Bialasiewicz, P. (1999). Increased content of thiobarbituric acid-reactive substances and hydrogen peroxide in the expired breath condensate of patients with stable chronic obstructive pulmonary disease: No significant effect of cigarette smoking. *Respiratory Medicine*, *93*(6), 389–396. https://doi.org/10.1053/rmed.1999.0574

Nussey, D. H., Coulson, T., Delorme, D., Clutton-Brock, T. H., Pemberton, J. M., Festa-Bianchet, M., & Gaillard, J.-M. (2011). Patterns of body mass senescence and selective

disappearance differ among three species of free-living ungulates. *Ecology*, *92*(10), 1936–1947. https://doi.org/10.1890/11-0308.1

Nussey, D. H., Pemberton, J. M., Pilkington, J. G., & Blount, J. D. (2009). Life history correlates of oxidative damage in a free-living mammal population. *Functional Ecology*, *23*(4), 809–817. https://doi.org/10.1111/j.1365-2435.2009.01555.x

Olive, P. L., & Banáth, J. P. (2006). The comet assay: A method to measure DNA damage in individual cells. *Nature Protocols*, *1*(1), 23–29. https://doi.org/10.1038/nprot.2006.5

Pham-Huy, L. A., He, H., & Pham-Huy, C. (2008). Free Radicals, Antioxidants in Disease and Health. *International Journal of Biomedical Science : IJBS*, *4*(2), 89–96.

Pilger, A., Germadnik, D., Schaffer, A., Theiler, A., Pils, P., Sluka, F., Winker, N., & Rüdiger, H. W. (2000). 8-Hydroxydeoxyguanosine in leukocyte DNA and urine of quartz-exposed workers and patients with silicosis. *International Archives of Occupational and Environmental Health*, *73*(5), 305–310. https://doi.org/10.1007/s004200000117

Pinchuk, I., & Lichtenberg, D. (2014). Analysis of the kinetics of lipid peroxidation in terms of characteristic time-points. *Chemistry and Physics of Lipids*, *178*, 63–76. https://doi.org/10.1016/j.chemphyslip.2013.12.001

Pizzino, G., Irrera, N., Cucinotta, M., Pallio, G., Mannino, F., Arcoraci, V., Squadrito, F., Altavilla, D., & Bitto, A. (2017). Oxidative Stress: Harms and Benefits for Human Health. *Oxidative Medicine and Cellular Longevity*, *2017*, 8416763–8416763. https://doi.org/10.1155/2017/8416763

Praticò, D. (2002). Lipid Peroxidation and the Aging Process. *Sci. Aging Knowl. Environ.*, *2002*(50), re5-. https://doi.org/10.1126/sageke.2002.50.re5

Raichlen, D. A., & Alexander, G. E. (2017). Adaptive Capacity: An Evolutionary Neuroscience Model Linking Exercise, Cognition, and Brain Health. *Trends in Neurosciences*, *40*(7), 408–421. https://doi.org/10.1016/j.tins.2017.05.001

Raichlen, D. A., Pontzer, H., Harris, J. A., Mabulla, A. Z. P., Marlowe, F. W., Josh Snodgrass, J., Eick, G., Colette Berbesque, J., Sancilio, A., & Wood, B. M. (2017). Physical activity patterns and biomarkers of cardiovascular disease risk in hunter-gatherers. *American Journal of Human Biology*, *29*(2), e22919. https://doi.org/10.1002/ajhb.22919

Roberts, L. J., & Morrow, J. D. (2000). Measurement of F2-isoprostanes as an index of oxidative stress in vivo. *Free Radical Biology and Medicine*, *28*(4), 505–513. https://doi.org/10.1016/S0891-5849(99)00264-6

Sahlin, K., Ekberg, K., & Cizinsky, S. (1991). Changes in plasma hypoxanthine and free radical markers during exercise in man. *Acta Physiologica Scandinavica*, *142*(2), 275–281. https://doi.org/10.1111/j.1748-1716.1991.tb09157.x

Schwarz, K. B. (1996). Oxidative stress during viral infection: A review. *Free Radical Biology and Medicine*, *21*(5), 641–649. https://doi.org/10.1016/0891-5849(96)00131-1

Scully, E. J., Basnet, S., Wrangham, R. W., Muller, M. N., Otali, E., Hyeroba, D., Grindle, K. A., Pappas, T. E., Emery Thompson, M., Machanda, Z., Watters, K. E., Palmenberg, A. C., Gern, J. E., & Goldberg, T. L. (2018). Lethal respiratory disease associated with human Rhinovirus C in wild chimpanzees, Uganda, 2013. *Emerging Infectious Disease*, *24*(2), 267–274. https://doi.org/10.3201/eid2402.170778

Sekhar, R., Kumar, P., & Liu, C. (2018). REVERSING AGING: PREVENTING AGE-RELATED DECLINE IN GLUTATHIONE AND MITOCHONDRIAL FUNCTION INCREASES LONGEVITY. *Innovation in Aging*, *2*(suppl_1), 887–887. https://doi.org/10.1093/geroni/igy031.3309

Selman, C., Blount, J. D., Nussey, D. H., & Speakman, J. R. (2012). Oxidative damage, ageing, and life-history evolution: Where now? *Trends in Ecology & Evolution*, *27*(10), 570–577. https://doi.org/10.1016/j.tree.2012.06.006

Simm, A., Nass, N., Bartling, B., Hofmann, B., Silber, R.-E., & Navarrete Santos, A. (2008). *Potential biomarkers of ageing*.

Sohal, R. S., & Orr, W. C. (2012). The redox stress hypothesis of aging. *Free Radical Biology and Medicine*, *52*(3), 539–555. https://doi.org/10.1016/j.freeradbiomed.2011.10.445

Soulsbury, C. D., & Halsey, L. G. (2018). Does physical activity age wild animals? *Frontiers in Ecology and Evolution*, *6*(222). https://doi.org/10.3389/fevo.2018.00222

Speakman, J. R., Blount, J. D., Bronikowski, A. M., Buffenstein, R., Isaksson, C., Kirkwood, T. B. L., Monaghan, P., Ozanne, S. E., Beaulieu, M., Briga, M., Carr, S. K., Christensen, L. L., Cochemé, H. M., Cram, D. L., Dantzer, B., Harper, J. M., Jurk, D., King, A., Noguera, J. C., … Selman, C. (2015). Oxidative stress and life histories: Unresolved issues and current needs. *Ecology and Evolution*, *5*(24), 5745–5757. https://doi.org/10.1002/ece3.1790

Thomas, D. C. (2017). The phagocyte respiratory burst: Historical perspectives and recent advances. *Immunology Letters*, *192*, 88–96. https://doi.org/10.1016/j.imlet.2017.08.016

Tucker, P. S., Dalbo, V. J., Han, T., & Kingsley, M. I. (2013). Clinical and research markers of oxidative stress in chronic kidney disease. *Biomarkers*, *18*(2), 103–115. https://doi.org/10.3109/1354750X.2012.749302

Whiting, P. H., Kalansooriya, A., Holbrook, I., Haddad, F., & Jennings, P. E. (2008). The relationship between chronic glycaemic control and oxidative stress in type 2 diabetes mellitus. *British Journal of Biomedical Science*, *65*(2), 71–74. https://doi.org/10.1080/09674845.2008.11732800

Wolf, F. I., Fasanella, S., Tedesco, B., Cavallini, G., Donati, A., Bergamini, E., & Cittadini, A. (2005). Peripheral lymphocyte 8-OHdG levels correlate with age-associated increase of tissue oxidative DNA damage in Sprague–Dawley rats. Protective effects of caloric restriction. *Experimental Gerontology*, *40*(3), 181–188. https://doi.org/10.1016/j.exger.2004.11.002

Wood, B. M., Watts, D. P., Mitani, J. C., & Langergraber, K. E. (2017). Favorable ecological circumstances promote life expectancy in chimpanzees similar to that of human hunter-gatherers. *Journal of Human Evolution*, *105*, 41–56. https://doi.org/10.1016/j.jhevol.2017.01.003

Wrigley-Field, E. (2014). Mortality deceleration and mortality selection: Three unexpected implications of a simple model. *Demography*, *51*(1), 51–71. https://doi.org/10.1007/s13524-013-0256-7

Wu, D. F., Behringer, V., Wittig, R. M., Leendertz, F. H., & Deschner, T. (2018). Urinary neopterin levels increase and predict survival during a respiratory outbreak in wild chimpanzees (Taï National Park, Côte d'Ivoire). *Scientific Reports*, *8*(1), 13346. https://doi.org/10.1038/s41598-018-31563-7

Wu, D., Liu, B., Yin, J., Xu, T., Zhao, S., Xu, Q., Chen, X., & Wang, H. (2017). Detection of 8-hydroxydeoxyguanosine (8-OHdG) as a biomarker of oxidative damage in peripheral leukocyte DNA by UHPLC–MS/MS. *Journal of Chromatography B*, *1064*, 1–6. https://doi.org/10.1016/j.jchromb.2017.08.033

Yan, W., Byrd, G. D., & Ogden, M. W. (2007). Quantitation of isoprostane isomers in human urine from smokers and nonsmokers by LC-MS/MS. *Journal of Lipid Research*, *48*(7), 1607–1617.

Zhou, L., Zhang, H., Davies, K. J. A., & Forman, H. J. (2018). Aging-related decline in the induction of Nrf2-regulated antioxidant genes in human bronchial epithelial cells. *Redox Biology*, *14*, 35–40. https://doi.org/10.1016/j.redox.2017.08.014

Zinman, G., Brower-Sinning, R., Emeche, C. H., Ernst, J., Huang, G. T.-W., Mahony, S., Myers, A. J., O'Dee, D. M., Flynn, J. L., Nau, G. J., Ross, T. M., Salter, R. D., Benos, P. V., Bar Joseph, Z., & Morel, P. A. (2011). Large scale comparison of innate responses to viral and bacterial pathogens in mouse and macaque. *PloS One*, *6*(7), e22401. https://doi.org/10.1371/journal.pone.0022401

Babior, B. M. (2000). Phagocytes and oxidative stress. *The American Journal of Medicine*, *109*(1), 33–44. https://doi.org/10.1016/S0002-9343(00)00481-2

Balakrishnan, S. D., & Anuradha, C. V. (1998). Exercise, depletion of antioxidants and antioxidant manipulation. *Cell Biochemistry and Function*, *16*(4), 269–275. https://doi.org/10.1002/(SICI)1099-0844(1998120)16:4<269::AID-CBF797>3.0.CO;2-B

Bartz, R. R., & Piantadosi, C. A. (2010). Clinical review: Oxygen as a signaling molecule. *Critical Care (London, England)*, *14*(5), 234–234. https://doi.org/10.1186/cc9185

Basu, S. (2008). F2-Isoprostanes in Human Health and Diseases: From Molecular Mechanisms to Clinical Implications. *Antioxidants & Redox Signaling*, *10*(8), 1405–1434. https://doi.org/10.1089/ars.2007.1956

Behringer, V., Stevens, J., Leendertz, F., Hohmann, G., & Deschner, T. (2017). Validation of a method for the assessment of urinary neopterin levels to monitor health status in non-human-primate species. *Frontiers in Physiology*, *8*(51). https://doi.org/10.3389/fphys.2017.00051

Bektas, A., Schurman, S. H., Sen, R., & Ferrucci, L. (2017). Human T cell immunosenescence and inflammation in aging. *Journal of Leukocyte Biology*, *102*(4), 977–988. https://doi.org/10.1189/jlb.3RI0716-335R

Belsky, D. W., Caspi, A., Houts, R., Cohen, H. J., Corcoran, D. L., Danese, A., Harrington, H., Israel, S., Levine, M. E., Schaefer, J. D., Sugden, K., Williams, B., Yashin, A. I., Poulton, R., & Moffitt, T. E. (2015). Quantification of biological aging in young adults. *Proceedings of the National Academy of Sciences*, *112*(30), E4104. https://doi.org/10.1073/pnas.1506264112

Capuron, L., Geisler, S., Kurz, K., Leblhuber, F., Sperner-Unterweger, B., & Fuchs, D. (2014). Activated Immune System and Inflammation in Healthy Ageing: Relevance for Tryptophan and Neopterin Metabolism. *Current Pharmaceutical Design*, *20*(38), 6048–6057.

Cayman Chemical. (2016). *Protein Carbonyl Colorimetric Assay Kit*. https://www.caymanchem.com/pdfs/10005020.pdf

Chen, J.-H., Hales, C. N., & Ozanne, S. E. (2007). DNA damage, cellular senescence and organismal ageing: Causal or correlative? *Nucleic Acids Research*, *35*(22), 7417–7428.

https://doi.org/10.1093/nar/gkm681

Chen, Q. M., & Maltagliati, A. J. (2018). Nrf2 at the heart of oxidative stress and cardiac protection. *Physiological Genomics*, *50*(2), 77–97. https://doi.org/10.1152/physiolgenomics.00041.2017

Cooke, M. S., Evans, M. D., Dove, R., Rozalski, R., Gackowski, D., Siomek, A., Lunec, J., & Olinski, R. (2005). DNA repair is responsible for the presence of oxidatively damaged DNA lesions in urine. *Genes and Environment*, *574*(1), 58–66. https://doi.org/10.1016/j.mrfmmm.2005.01.022

Costantini, D. (2011). On the measurement of circulating antioxidant capacity and the nightmare of uric acid. *Methods in Ecology and Evolution*, *2*(3), 321–325. https://doi.org/doi:10.1111/j.2041-210X.2010.00080.x

Daynes, R. A., Enioutina, E. Y., & Jones, D. C. (2003). Role of Redox Imbalance in the Molecular Mechanisms Responsible for Immunosenescence. *Antioxidants & Redox Signaling*, *5*(5), 537–548. https://doi.org/10.1089/152308603770310185

Denz, H., Fuchs, D., Hausen, A., Huber, H., Nachbaur, D., Reibnegger, G., Thaler, J., Werner, E. R., & Wachter, H. (1990). Value of urinary neopterin in the differential diagnosis of bacterial and viral infections. *Klinische Wochenschrift*, *68*(4), 218–222. https://doi.org/10.1007/BF01662720

Dorjgochoo, T., Gao, Y.-T., Chow, W.-H., Shu, X., Yang, G., Cai, Q., Rothman, N., Cai, H., Li, H., Deng, X., Franke, A., Roberts, L. J., Milne, G., Zheng, W., & Dai, Q. (2012). Major metabolite of F2-isoprostane in urine may be a more sensitive biomarker of oxidative stress than isoprostane itself. *The American Journal of Clinical Nutrition*, *96*(2), 405–414. https://doi.org/10.3945/ajcn.112.034918

Duplancic, D., Kukoc-Modun, L., Modun, D., & Radic, N. (2011). Simple and Rapid Method for the Determination of Uric Acid-Independent Antioxidant Capacity. *Molecules*, *16*(8). https://doi.org/10.3390/molecules16087058

Emery Thompson, M., Machanda Zarin, P., Scully Erik, J., Enigk Drew, K., Otali, E., Muller Martin, N., Goldberg Tony, L., Chapman Colin, A., & Wrangham Richard, W. (2018). Risk factors for respiratory illness in a community of wild chimpanzees (Pan troglodytes schweinfurthii). *Royal Society Open Science*, *5*(9), 180840. https://doi.org/10.1098/rsos.180840

Feillet-Coudray, C., Tourtauchaux, R., Niculescu, M., Rock, E., Tauveron, I., Alexandre-Gouabau, M.-C., Rayssiguier, Y., Jalenques, I., & Mazur, A. (1999). Plasma levels of 8-epiPGF2α, an in vivo marker of oxidative stress, are not affected by aging or Alzheimer's disease. *Free Radical Biology and Medicine*, *27*(3), 463–469. https://doi.org/10.1016/S0891-5849(99)00096-9

Finkel, T., & Holbrook, N. J. (2000). Oxidants, oxidative stress and the biology of ageing. *Nature*, *408*(6809), 239–247.

Fletcher, Q. E., Selman, C., Boutin, S., McAdam, A. G., Woods, S. B., Seo, A. Y., Leeuwenburgh, C., Speakman, J. R., & Humphries, M. M. (2012). Oxidative damage increases with reproductive energy expenditure and is reduced by food-supplementation. *Evolution*, no-no. https://doi.org/10.1111/evo.12014

Fraga, C. G., Shigenaga, M. K., Park, J. W., Degan, P., & Ames, B. N. (1990). Oxidative damage to DNA during aging: 8-hydroxy-2'-deoxyguanosine in rat organ DNA and urine. *Proceedings of the National Academy of Sciences*, *87*(12), 4533. https://doi.org/10.1073/pnas.87.12.4533

Franceschi, C., & Campisi, J. (2014). Chronic Inflammation (Inflammaging) and Its Potential Contribution to Age-Associated Diseases. *The Journals of Gerontology: Series A*, *69*(Suppl_1), S4–S9. https://doi.org/10.1093/gerona/glu057

Franceschi, C., Garagnani, P., Parini, P., Giuliani, C., & Santoro, A. (2018). Inflammaging: A new immune–metabolic viewpoint for age-related diseases. *Nature Reviews Endocrinology*, *14*(10), 576–590. https://doi.org/10.1038/s41574-018-0059-4

Frisard, M. I., Broussard, A., Davies, S. S., Roberts, L. J., Rood, J., de Jonge, L., Fang, X., Jazwinski, S. M., Deutsch, W. A., & Ravussin, E. (2007). Aging, Resting Metabolic Rate, and Oxidative Damage: Results From the Louisiana Healthy Aging Study. *The Journals of Gerontology: Series A*, *62*(7), 752–759. https://doi.org/10.1093/gerona/62.7.752

Fulop, T., Larbi, A., Dupuis, G., Le Page, A., Frost, E. H., Cohen, A. A., Witkowski, J. M., & Franceschi, C. (2018). Immunosenescence and Inflamm-Aging As Two Sides of the Same Coin: Friends or Foes? *Frontiers in Immunology*, *8*. https://doi.org/10.3389/fimmu.2017.01960

Gan, W., Liu, X.-L., Yu, T., Zou, Y.-G., Li, T.-T., Wang, S., Deng, J., Wang, L.-L., & Cai, J.-P. (2018). Urinary 8-oxo-7,8-dihydroguanosine as a Potential Biomarker of Aging. *Frontiers in Aging Neuroscience*, *10*. https://doi.org/10.3389/fnagi.2018.00034

Gan, W., Nie, B., Shi, F., Xu, X.-M., Qian, J.-C., Takagi, Y., Hayakawa, H., Sekiguchi, M., & Cai, J.-P. (2012). Age-dependent increases in the oxidative damage of DNA, RNA, and their metabolites in normal and senescence-accelerated mice analyzed by LC–MS/MS: Urinary 8-oxoguanosine as a novel biomarker of aging. *Free Radical Biology and Medicine*, *52*(9), 1700–1707. https://doi.org/10.1016/j.freeradbiomed.2012.02.016

Georgiev, A. V., Thompson, M. E., Mandalaywala, T. M., & Maestripieri, D. (2015). Oxidative stress as an indicator of the costs of reproduction among free-ranging rhesus macaques. *The Journal of Experimental Biology*, *218*(13), 1981. https://doi.org/10.1242/jeb.121947

Ghiselli, A., Serafini, M., Natella, F., & Scaccini, C. (2001). Total antioxidant capacity as a tool to assess redox status: Critical view and experimental data. In *Bio-Assays for Oxidative Stress Status* (pp. 219–227). Elsevier.

Gorbunova, V., Seluanov, A., Mao, Z., & Hine, C. (2007). Changes in DNA repair during aging. *Nucleic Acids Research*, *35*(22), 7466–7474. https://doi.org/10.1093/nar/gkm756

Gurven, M., Kaplan, H., Winking, J., Eid Rodriguez, D., Vasunilashorn, S., Kim, J. K., Finch, C., & Crimmins, E. (2009). Inflammation and Infection Do Not Promote Arterial Aging and Cardiovascular Disease Risk Factors among Lean Horticulturalists. *PLOS ONE*, *4*(8), e6590. https://doi.org/10.1371/journal.pone.0006590

Gurven, M., Kaplan, H., Winking, J., Finch, C., & Crimmins, E. M. (2008). Aging and Inflammation in Two Epidemiological Worlds. *The Journals of Gerontology: Series A*, *63*(2), 196–199. https://doi.org/10.1093/gerona/63.2.196

Halliwell, B. (2006). Reactive Species and Antioxidants. Redox Biology Is a Fundamental Theme of Aerobic Life. *Plant Physiology*, *141*(2), 312. https://doi.org/10.1104/pp.106.077073

Hamilton, M. L., Van Remmen, H., Drake, J. A., Yang, H., Guo, Z. M., Kewitt, K., Walter, C. A., & Richardson, A. (2001). Does oxidative damage to DNA increase with age? *Proceedings of the National Academy of Sciences*, *98*(18), 10469. https://doi.org/10.1073/pnas.171202698

Handa, J. T., Cano, M., Wang, L., Datta, S., & Liu, T. (2017). Lipids, oxidized lipids, oxidation-specific epitopes, and Age-related Macular Degeneration. *Biochimica et Biophysica Acta. Molecular and Cell Biology of Lipids*, *1862*(4), 430–440. https://doi.org/10.1016/j.bbalip.2016.07.013

Hardeland, R., Coto- Montes, A., & Poeggeler, B. (2003). Circadian Rhythms, Oxidative Stress, and Antioxidative Defense Mechanisms. *Chronobiology International*, *20*(6), 921–962. https://doi.org/10.1081/CBI-120025245

Harman, D. (1956). Aging: A theory based on free radical and radiation chemistry. *Journal of Gerontology*, *11*, 298–300.

Heistermann, M., & Higham, J. P. (2015). Urinary neopterin, a non-invasive marker of mammalian cellular actiation, is highly stable under field conditions. *Scientific Reports*. https://doi.org/10.1038/srep16308

Higham, J. P., Kraus, C., Stahl-Hennig, C., Engelhardt, A., Fuchs, D., & Heistermann, M. (2015). Evaluating noninvasive markers of nonhuman primate immune activation and inflammation. *American Journal of Physical Anthropology*, *158*(4), 673–684. https://doi.org/10.1002/ajpa.22821

Hill, K., Boesch, C., Goodall, J., Pusey, A., Williams, J., & Wrangham, R. (2001). Mortality rates among wild chimpanzees. *Journal of Human Evolution*, *40*(5), 437–450. https://doi.org/10.1006/jhev.2001.0469

Hybertson, B. M., Gao, B., Bose, S. K., & McCord, J. M. (2011). Oxidative stress in health and disease: The therapeutic potential of Nrf2 activation. *Molecular Aspects of Medicine*, *32*(4), 234–246. https://doi.org/10.1016/j.mam.2011.10.006

Ialongo, C. (2017). Preanalytic of total antioxidant capacity assays performed in serum, plasma, urine and saliva. *Clinical Biochemistry*, *50*(6), 356–363. https://doi.org/10.1016/j.clinbiochem.2016.11.037

Il'yasova, D., Mixon, G., Wang, F., Marcom, P. K., Marks, J., Spasojevich, I., Craft, N., Arredondo, F., & DiGiulio, R. (2009). Markers of oxidative status in a clinical model of oxidative assault: A pilot study in human blood following doxorubicin administration. *Biomarkers*, *14*(5), 321–325. https://doi.org/10.1080/13547500902946757

Il'yasova, D., Scarbrough, P., & Spasojevic, I. (2012). Urinary biomarkers of oxidative status. *Clinica Chimica Acta*, *413*(19), 1446–1453. https://doi.org/10.1016/j.cca.2012.06.012

Il'yasova, D., Spasojevic, I., Wang, F., Tolun, A. A., Base, K., Young, S. P., Marcom, P. K., Marks, J., Mixon, G., DiGiulio, R., & Millington, D. S. (2010). Urinary biomarkers of oxidative status in a clinical model of oxidative assault. *Cancer Epidemiology Biomarkers & Prevention*, *19*(6), 1506. https://doi.org/10.1158/1055-9965.EPI-10-0211

Imlay, J. A. (2003). Pathways of Oxidative Damage. *Annual Review of Microbiology*, *57*(1), 395–418. https://doi.org/10.1146/annurev.micro.57.030502.090938

İnal, M. E., Kanbak, G., & Sunal, E. (2001). Antioxidant enzyme activities and malondialdehyde levels related to aging. *Clinica Chimica Acta*, *305*(1), 75–80. https://doi.org/10.1016/S0009-8981(00)00422-8

Jain, S. K., Pemberton, P. W., Smith, A., McMahon, R. F. T., Burrows, P. C., Aboutwerat, A., & Warnes, T. W. (2002). Oxidative stress in chronic hepatitis C: not just a feature of late stage disease. *Journal of Hepatology*, *36*(6), 805–811. https://doi.org/10.1016/S0168-8278(02)00060-0

Kadiiska, M. B., Gladen, B. C., Baird, D. D., Germolec, D., Graham, L. B., Parker, C. E.,

Nyska, A., Wachsman, J. T., Ames, B. N., Basu, S., Brot, N., FitzGerald, G. A., Floyd, R. A., George, M., Heinecke, J. W., Hatch, G. E., Hensley, K., Lawson, J. A., Marnett, L. J., … Barrett, J. C. (2005). Biomarkers of Oxidative Stress Study II: Are oxidation products of lipids, proteins, and DNA markers of CCl4 poisoning? *Free Radical Biology and Medicine*, *38*(6), 698–710. https://doi.org/10.1016/j.freeradbiomed.2004.09.017

Kanabrocki, E. L., Murray, D., Hermida, R. C., Scott, G. S., Bremner, W. F., Ryan, M. D., Ayala, D. E., Third, J. L. H. C., Shirazi, P., Nemchausky, B. A., & Hooper, D. C. (2002). Circadian variation in oxidative stress markers in healthy and type II diabetic men. *Chronobiology International*, *19*(2), 423–439. https://doi.org/10.1081/CBI-120002914

Kirkwood, T. B. L., & Melov, S. (2011). On the Programmed/Non-Programmed Nature of Ageing within the Life History. *Current Biology*, *21*(18), R701–R707. https://doi.org/10.1016/j.cub.2011.07.020

Koracevic, D., Koracevic, G., Djordjevic, V., Andrejevic, S., & Cosic, V. (2001). Method for the measurement of antioxidant activity in human fluids. *Journal of Clinical Pathology*, *54*(5), 356. https://doi.org/10.1136/jcp.54.5.356

Korkmaz, K. S., Butuner, B. D., & Roggenbuck, D. (2018). Detection of 8-OHdG as a diagnostic biomarker. *Journal of Laboratory and Precision Medicine; November 2018*.

Kregel, K. C., & Zhang, H. J. (2007). An integrated view of oxidative stress in aging: Basic mechanisms, functional effects, and pathological considerations. *American Journal of Physiology-Regulatory, Integrative and Comparative Physiology*, *292*(1), R18–R36. https://doi.org/10.1152/ajpregu.00327.2006

Lee, & Kang, D. (2008). Stability and Intra-Individual Variation of Urinary Malondialdehyde and 2-Naphthol. *Journal of Preventative Medicine and Public Health*, *41*(3), 195–199. https://doi.org/2008.41.3.195

Lee, S. G., Wang, T., Vance, T. M., Hubert, P., Kim, D.-O., Koo, S. I., & Chun, O. K. (2017). Validation of Analytical Methods for Plasma Total Antioxidant Capacity by Comparing with Urinary 8-Isoprostane Level. *Journal of Microbiology and Biotechnology*, *27*(2), 388–394. https://doi.org/10.4014/jmb.1604.04053

Li, Z., Xu, X., Leng, X., He, M., Wang, J., Cheng, S., & Wu, H. (2017). Roles of reactive oxygen species in cell signaling pathways and immune responses to viral infections. *Archives of Virology*, *162*(3), 603–610. https://doi.org/10.1007/s00705-016-3130-2

Liu, T., Stern, A., Roberts, L. J., & Morrow, J. D. (1999). The Isoprostanes: Novel Prostaglandin-Like Products of the Free Radical-Catalyzed Peroxidation of Arachidonic Acid. *Journal of Biomedical Science*, *6*(4), 226–235. https://doi.org/10.1159/000025391

Longmire, A. W., Swift, L. L., Jackson Roberts, L., Awad, J. A., Burk, R. F., & Morrow, J. D. (1994). Effect of oxygen tension on the generation of F2-isoprostanes and malondialdehyde in peroxidizing rat liver microsomes. *Biochemical Pharmacology*, *47*(7), 1173–1177. https://doi.org/10.1016/0006-2952(94)90389-1

Lushchak, V. I. (2014). Free radicals, reactive oxygen species, oxidative stress and its classification. *Chemico-Biological Interactions*, *224*, 164–175. https://doi.org/10.1016/j.cbi.2014.10.016

MacKinnon, K. L., Molnar, Z., Lowe, D., Watson, I. D., & Shearer, E. (1999). Measures of total free radical activity in critically ill patients. *Clinical Biochemistry*, *32*(4), 263–268. https://doi.org/10.1016/S0009-9120(98)00109-X

Martin, L. J. (2008). DNA Damage and Repair: Relevance to Mechanisms of

Neurodegeneration. *Journal of Neuropathology & Experimental Neurology*, *67*(5), 377–387. https://doi.org/10.1097/NEN.0b013e31816ff780

Martínez de Toda, I., Vida, C., Garrido, A., & De la Fuente, M. (2019). Redox Parameters as Markers of the Rate of Aging and Predictors of Life Span. *The Journals of Gerontology: Series A*. https://doi.org/10.1093/gerona/glz033

Martin-Montalvo, A., Mercken, E. M., Mitchell, S. J., Palacios, H. H., Mote, P. L., Scheibye-Knudsen, M., Gomes, A. P., Ward, T. M., Minor, R. K., Blouin, M.-J., Schwab, M., Pollak, M., Zhang, Y., Yu, Y., Becker, K. G., Bohr, V. A., Ingram, D. K., Sinclair, D. A., Wolf, N. S., … de Cabo, R. (2013). Metformin improves healthspan and lifespan in mice. *Nature Communications*, *4*, 2192. https://doi.org/10.1038/ncomms3192 https://www.nature.com/articles/ncomms3192#supplementary-information

Matsumoto, M., Awano, H., Nagai, M., Shirakawa, T., Iijima, K., & Matsuo, M. (2017). Urinary excretion of 8-OHdG, a biomarker of oxidative DNA damage, increases with age in DMD patients. *Neuromuscular Disorders*, *27*, S168–S169. https://doi.org/10.1016/j.nmd.2017.06.274

Maxwell, S. R., Jakeman, P., Thomason, H., Leguen, C., & Thorpe, G. H. (1993). Changes in plasma antioxidant status during eccentric exercise and the effect of vitamin supplementation. *Free Radic Res Commun*, *19*(3), 191–202.

Maynard, S., Fang, E. F., Scheibye-Knudsen, M., Croteau, D. L., & Bohr, V. A. (2015). DNA Damage, DNA Repair, Aging, and Neurodegeneration. *Cold Spring Harbor Perspectives in Medicine*, *5*(10), a025130. https://doi.org/10.1101/cshperspect.a025130

Melov, S. (2002). Animal models of oxidative stress, aging, and therapeutic antioxidant interventions. *The International Journal of Biochemistry & Cell Biology*, *34*(11), 1395–1400. https://doi.org/10.1016/s1357-2725(02)00086-9

Metcalfe, N. B., & Alonso-Alvarez, C. (2010). Oxidative stress as a life-history constraint: The role of reactive oxygen species in shaping phenotypes from conception to death. *Functional Ecology*, *24*(5), 984–996. https://doi.org/doi:10.1111/j.1365-2435.2010.01750.x

Metcalfe, N. B., & Monaghan, P. (2013). Does reproduction cause oxidative stress? An open question. *Trends in Ecology & Evolution*, *28*(6), 347–350. https://doi.org/10.1016/j.tree.2013.01.015

Miller, R. C., Brindle, E., Holman, D. J., Shofer, J., Klein, N. A., Soules, M. R., & O’Connor, K. A. (2004). Comparison of specific gravity and creatinine for normalizing urinary reproductive hormone concentrations. *Clinical Chemistry*, *50*(5), 924.

Moffitt, T. E., Belsky, D. W., Danese, A., Poulton, R., & Caspi, A. (2017). The Longitudinal study of aging in human young adults: Knowledge gaps and research agenda. *The Journals of Gerontology: Series A*, *72*(2), 210–215. https://doi.org/10.1093/gerona/glw191

Muller, M. N., & Wrangham, R. W. (2014). Mortality rates among Kanyawara chimpanzees. *Journal of Human Evolution*, *66*, 107–114. https://doi.org/10.1016/j.jhevol.2013.10.004

Müller, N., Heistermann, M., Strube, C., Schülke, O., & Ostner, J. (2017). Age, but not anthelmintic treatment, is associated with urinary neopterin levels in semi-free ranging Barbary macaques. *Scientific Reports*, *7*, 41973. https://doi.org/10.1038/srep41973

Murr, C., Widner, B., & Fuchs, B. W. and D. (2002). Neopterin as a Marker for Immune System Activation. *Current Drug Metabolism*, *3*(2), 175–187.

https://doi.org/10.2174/1389200024605082

Murr, Christian, Hainz, U., Asch, E., Berger, P., Jenewein, B., Saurwein-Teissl, M., Grubeck-Loebenstein, B., & Fuchs, D. (2003). Association of increased neopterin production with decreased humoral immunity in the elderly. *Experimental Gerontology*, *38*(5), 583–587. https://doi.org/10.1016/S0531-5565(03)00062-7

Nagel, Z. D., Margulies, C. M., Chaim, I. A., McRee, S. K., Mazzucato, P., Ahmad, A., Abo, R. P., Butty, V. L., Forget, A. L., & Samson, L. D. (2014). Multiplexed DNA repair assays for multiple lesions and multiple doses via transcription inhibition and transcriptional mutagenesis. *Proceedings of the National Academy of Sciences*, *111*(18), E1823. https://doi.org/10.1073/pnas.1401182111

Nakano, R., Nakagawa, R., Tokimoto, N., & Okanoya, K. (2013). Alarm call discrimination in a social rodent: Adult but not juvenile degu calls induce high vigilance. *Journal of Ethology*, 1–7.

Nowak, D., Kasielski, M., Antczak, A., Pietras, T., & Bialasiewicz, P. (1999). Increased content of thiobarbituric acid-reactive substances and hydrogen peroxide in the expired breath condensate of patients with stable chronic obstructive pulmonary disease: No significant effect of cigarette smoking. *Respiratory Medicine*, *93*(6), 389–396. https://doi.org/10.1053/rmed.1999.0574

Nussey, D. H., Coulson, T., Delorme, D., Clutton-Brock, T. H., Pemberton, J. M., Festa-Bianchet, M., & Gaillard, J.-M. (2011). Patterns of body mass senescence and selective disappearance differ among three species of free-living ungulates. *Ecology*, *92*(10), 1936–1947. https://doi.org/10.1890/11-0308.1

Nussey, D. H., Pemberton, J. M., Pilkington, J. G., & Blount, J. D. (2009). Life history correlates of oxidative damage in a free-living mammal population. *Functional Ecology*, *23*(4), 809–817. https://doi.org/10.1111/j.1365-2435.2009.01555.x

Olive, P. L., & Banáth, J. P. (2006). The comet assay: A method to measure DNA damage in individual cells. *Nature Protocols*, *1*(1), 23–29. https://doi.org/10.1038/nprot.2006.5

Pham-Huy, L. A., He, H., & Pham-Huy, C. (2008). Free Radicals, Antioxidants in Disease and Health. *International Journal of Biomedical Science : IJBS*, *4*(2), 89–96.

Pilger, A., Germadnik, D., Schaffer, A., Theiler, A., Pils, P., Sluka, F., Winker, N., & Rüdiger, H. W. (2000). 8-Hydroxydeoxyguanosine in leukocyte DNA and urine of quartz-exposed workers and patients with silicosis. *International Archives of Occupational and Environmental Health*, *73*(5), 305–310. https://doi.org/10.1007/s004200000117

Pinchuk, I., & Lichtenberg, D. (2014). Analysis of the kinetics of lipid peroxidation in terms of characteristic time-points. *Chemistry and Physics of Lipids*, *178*, 63–76. https://doi.org/10.1016/j.chemphyslip.2013.12.001

Pizzino, G., Irrera, N., Cucinotta, M., Pallio, G., Mannino, F., Arcoraci, V., Squadrito, F., Altavilla, D., & Bitto, A. (2017). Oxidative Stress: Harms and Benefits for Human Health. *Oxidative Medicine and Cellular Longevity*, *2017*, 8416763–8416763. https://doi.org/10.1155/2017/8416763

Praticò, D. (2002). Lipid Peroxidation and the Aging Process. *Sci. Aging Knowl. Environ.*, *2002*(50), re5-. https://doi.org/10.1126/sageke.2002.50.re5

Raichlen, D. A., & Alexander, G. E. (2017). Adaptive Capacity: An Evolutionary Neuroscience Model Linking Exercise, Cognition, and Brain Health. *Trends in*

Neurosciences, *40*(7), 408–421. https://doi.org/10.1016/j.tins.2017.05.001
Raichlen, D. A., Pontzer, H., Harris, J. A., Mabulla, A. Z. P., Marlowe, F. W., Josh Snodgrass, J., Eick, G., Colette Berbesque, J., Sancilio, A., & Wood, B. M. (2017). Physical activity patterns and biomarkers of cardiovascular disease risk in hunter-gatherers. *American Journal of Human Biology*, *29*(2), e22919. https://doi.org/10.1002/ajhb.22919
Roberts, L. J., & Morrow, J. D. (2000). Measurement of F2-isoprostanes as an index of oxidative stress in vivo. *Free Radical Biology and Medicine*, *28*(4), 505–513. https://doi.org/10.1016/S0891-5849(99)00264-6
Sahlin, K., Ekberg, K., & Cizinsky, S. (1991). Changes in plasma hypoxanthine and free radical markers during exercise in man. *Acta Physiologica Scandinavica*, *142*(2), 275–281. https://doi.org/10.1111/j.1748-1716.1991.tb09157.x
Schwarz, K. B. (1996). Oxidative stress during viral infection: A review. *Free Radical Biology and Medicine*, *21*(5), 641–649. https://doi.org/10.1016/0891-5849(96)00131-1
Scully, E. J., Basnet, S., Wrangham, R. W., Muller, M. N., Otali, E., Hyeroba, D., Grindle, K. A., Pappas, T. E., Emery Thompson, M., Machanda, Z., Watters, K. E., Palmenberg, A. C., Gern, J. E., & Goldberg, T. L. (2018). Lethal respiratory disease associated with human Rhinovirus C in wild chimpanzees, Uganda, 2013. *Emerging Infectious Disease*, *24*(2), 267–274. https://doi.org/10.3201/eid2402.170778
Sekhar, R., Kumar, P., & Liu, C. (2018). REVERSING AGING: PREVENTING AGE-RELATED DECLINE IN GLUTATHIONE AND MITOCHONDRIAL FUNCTION INCREASES LONGEVITY. *Innovation in Aging*, *2*(suppl_1), 887–887. https://doi.org/10.1093/geroni/igy031.3309
Selman, C., Blount, J. D., Nussey, D. H., & Speakman, J. R. (2012). Oxidative damage, ageing, and life-history evolution: Where now? *Trends in Ecology & Evolution*, *27*(10), 570–577. https://doi.org/10.1016/j.tree.2012.06.006
Simm, A., Nass, N., Bartling, B., Hofmann, B., Silber, R.-E., & Navarrete Santos, A. (2008). *Potential biomarkers of ageing*.
Sohal, R. S., & Orr, W. C. (2012). The redox stress hypothesis of aging. *Free Radical Biology and Medicine*, *52*(3), 539–555. https://doi.org/10.1016/j.freeradbiomed.2011.10.445
Soulsbury, C. D., & Halsey, L. G. (2018). Does physical activity age wild animals? *Frontiers in Ecology and Evolution*, *6*(222). https://doi.org/10.3389/fevo.2018.00222
Speakman, J. R., Blount, J. D., Bronikowski, A. M., Buffenstein, R., Isaksson, C., Kirkwood, T. B. L., Monaghan, P., Ozanne, S. E., Beaulieu, M., Briga, M., Carr, S. K., Christensen, L. L., Cochemé, H. M., Cram, D. L., Dantzer, B., Harper, J. M., Jurk, D., King, A., Noguera, J. C., … Selman, C. (2015). Oxidative stress and life histories: Unresolved issues and current needs. *Ecology and Evolution*, *5*(24), 5745–5757. https://doi.org/10.1002/ece3.1790
Thomas, D. C. (2017). The phagocyte respiratory burst: Historical perspectives and recent advances. *Immunology Letters*, *192*, 88–96. https://doi.org/10.1016/j.imlet.2017.08.016
Tucker, P. S., Dalbo, V. J., Han, T., & Kingsley, M. I. (2013). Clinical and research markers of oxidative stress in chronic kidney disease. *Biomarkers*, *18*(2), 103–115. https://doi.org/10.3109/1354750X.2012.749302
Whiting, P. H., Kalansooriya, A., Holbrook, I., Haddad, F., & Jennings, P. E. (2008). The

relationship between chronic glycaemic control and oxidative stress in type 2 diabetes mellitus. *British Journal of Biomedical Science*, *65*(2), 71–74. https://doi.org/10.1080/09674845.2008.11732800

Wolf, F. I., Fasanella, S., Tedesco, B., Cavallini, G., Donati, A., Bergamini, E., & Cittadini, A. (2005). Peripheral lymphocyte 8-OHdG levels correlate with age-associated increase of tissue oxidative DNA damage in Sprague–Dawley rats. Protective effects of caloric restriction. *Experimental Gerontology*, *40*(3), 181–188. https://doi.org/10.1016/j.exger.2004.11.002

Wood, B. M., Watts, D. P., Mitani, J. C., & Langergraber, K. E. (2017). Favorable ecological circumstances promote life expectancy in chimpanzees similar to that of human hunter-gatherers. *Journal of Human Evolution*, *105*, 41–56. https://doi.org/10.1016/j.jhevol.2017.01.003

Wrigley-Field, E. (2014). Mortality deceleration and mortality selection: Three unexpected implications of a simple model. *Demography*, *51*(1), 51–71. https://doi.org/10.1007/s13524-013-0256-7

Wu, D. F., Behringer, V., Wittig, R. M., Leendertz, F. H., & Deschner, T. (2018). Urinary neopterin levels increase and predict survival during a respiratory outbreak in wild chimpanzees (Taï National Park, Côte d'Ivoire). *Scientific Reports*, *8*(1), 13346. https://doi.org/10.1038/s41598-018-31563-7

Wu, D., Liu, B., Yin, J., Xu, T., Zhao, S., Xu, Q., Chen, X., & Wang, H. (2017). Detection of 8-hydroxydeoxyguanosine (8-OHdG) as a biomarker of oxidative damage in peripheral leukocyte DNA by UHPLC–MS/MS. *Journal of Chromatography B*, *1064*, 1–6. https://doi.org/10.1016/j.jchromb.2017.08.033

Yan, W., Byrd, G. D., & Ogden, M. W. (2007). Quantitation of isoprostane isomers in human urine from smokers and nonsmokers by LC-MS/MS. *Journal of Lipid Research*, *48*(7), 1607–1617.

Zhou, L., Zhang, H., Davies, K. J. A., & Forman, H. J. (2018). Aging-related decline in the induction of Nrf2-regulated antioxidant genes in human bronchial epithelial cells. *Redox Biology*, *14*, 35–40. https://doi.org/10.1016/j.redox.2017.08.014

Zinman, G., Brower-Sinning, R., Emeche, C. H., Ernst, J., Huang, G. T.-W., Mahony, S., Myers, A. J., O'Dee, D. M., Flynn, J. L., Nau, G. J., Ross, T. M., Salter, R. D., Benos, P. V., Bar Joseph, Z., & Morel, P. A. (2011). Large scale comparison of innate responses to viral and bacterial pathogens in mouse and macaque. *PloS One*, *6*(7), e22401. https://doi.org/10.1371/journal.pone.0022401

Click here to download Figure Fig1.tif

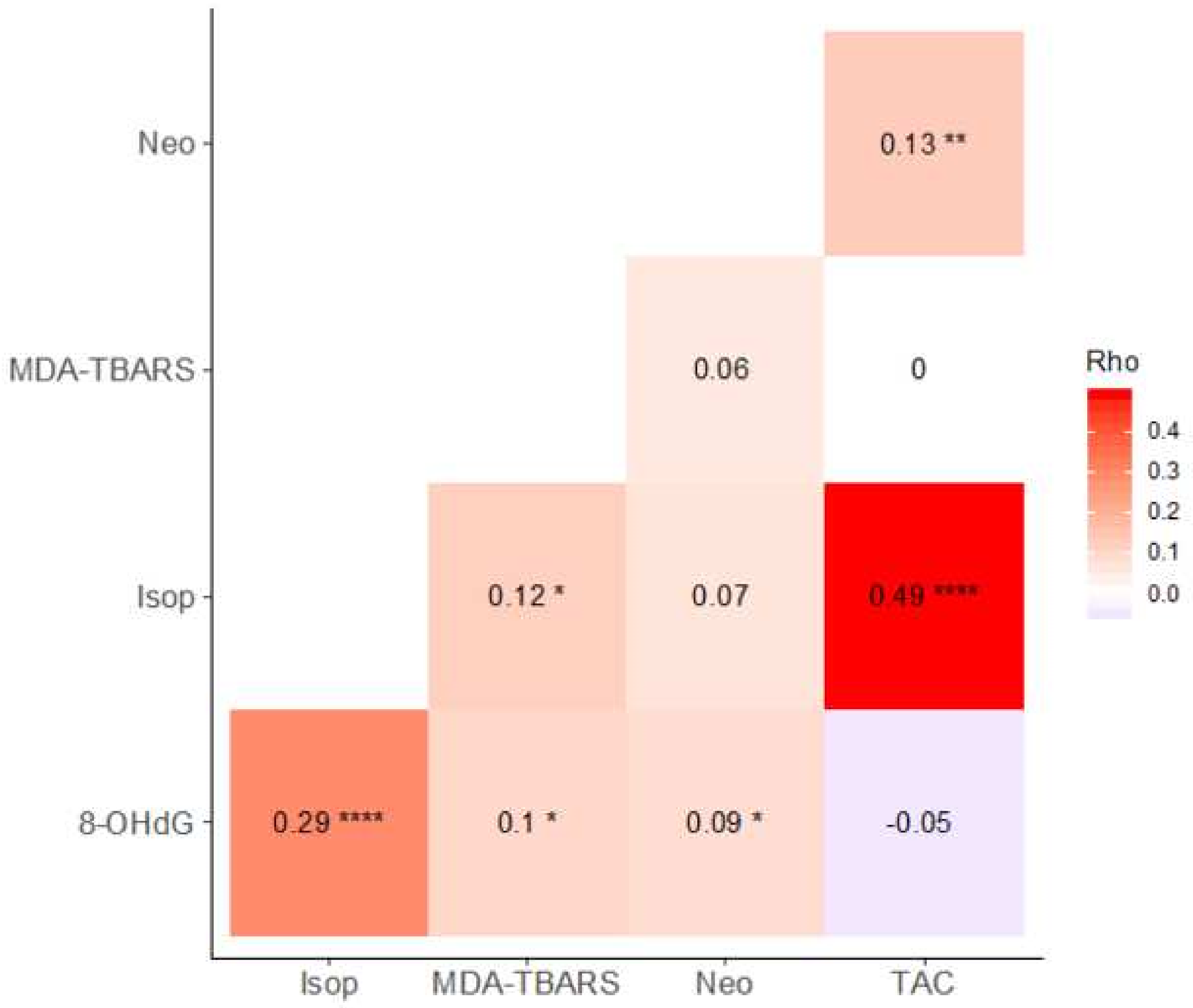

Click here to download Figure Fig2A1024_1.tif

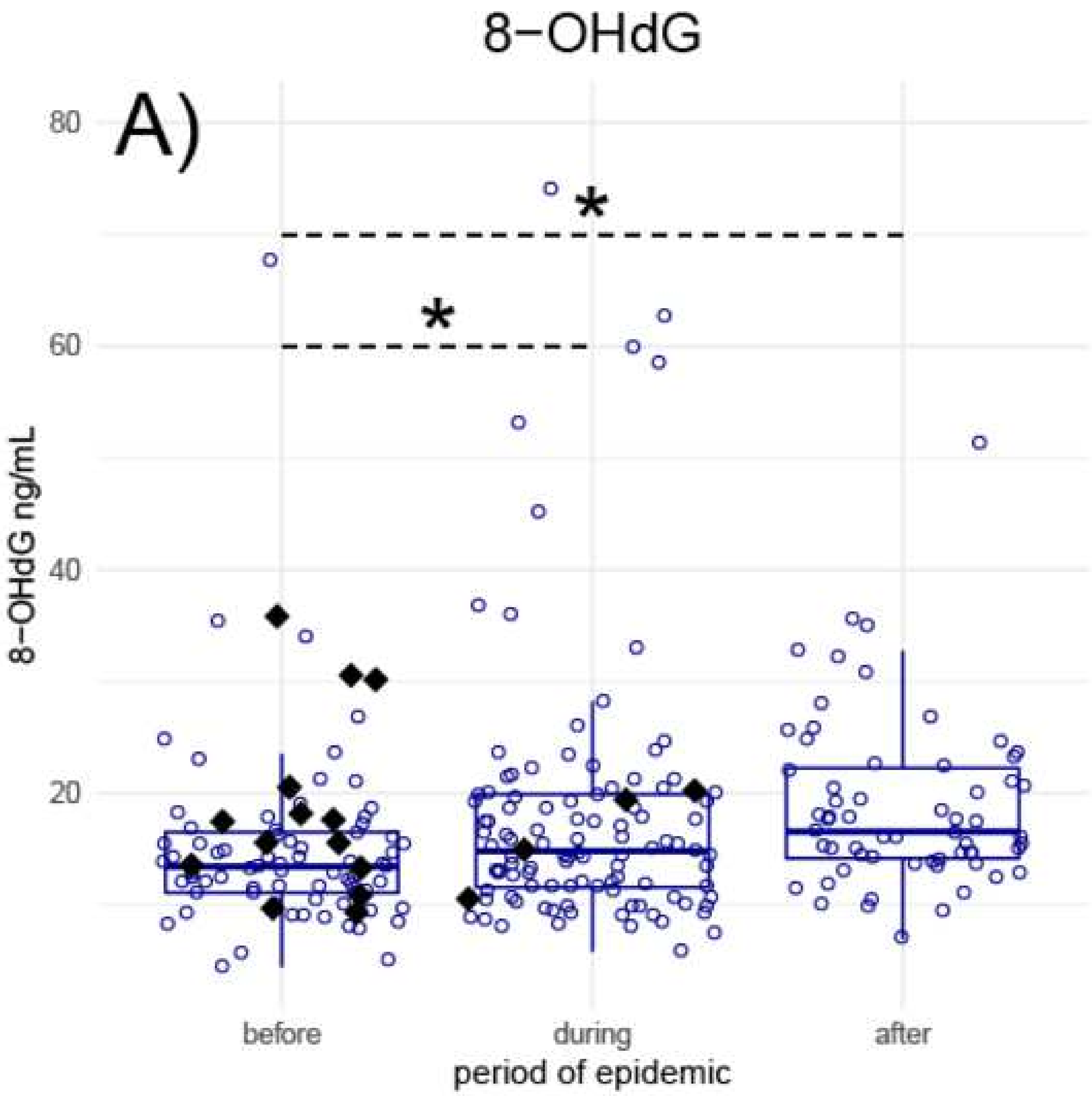

Click here to download Figure Fig2B1024_1.tif

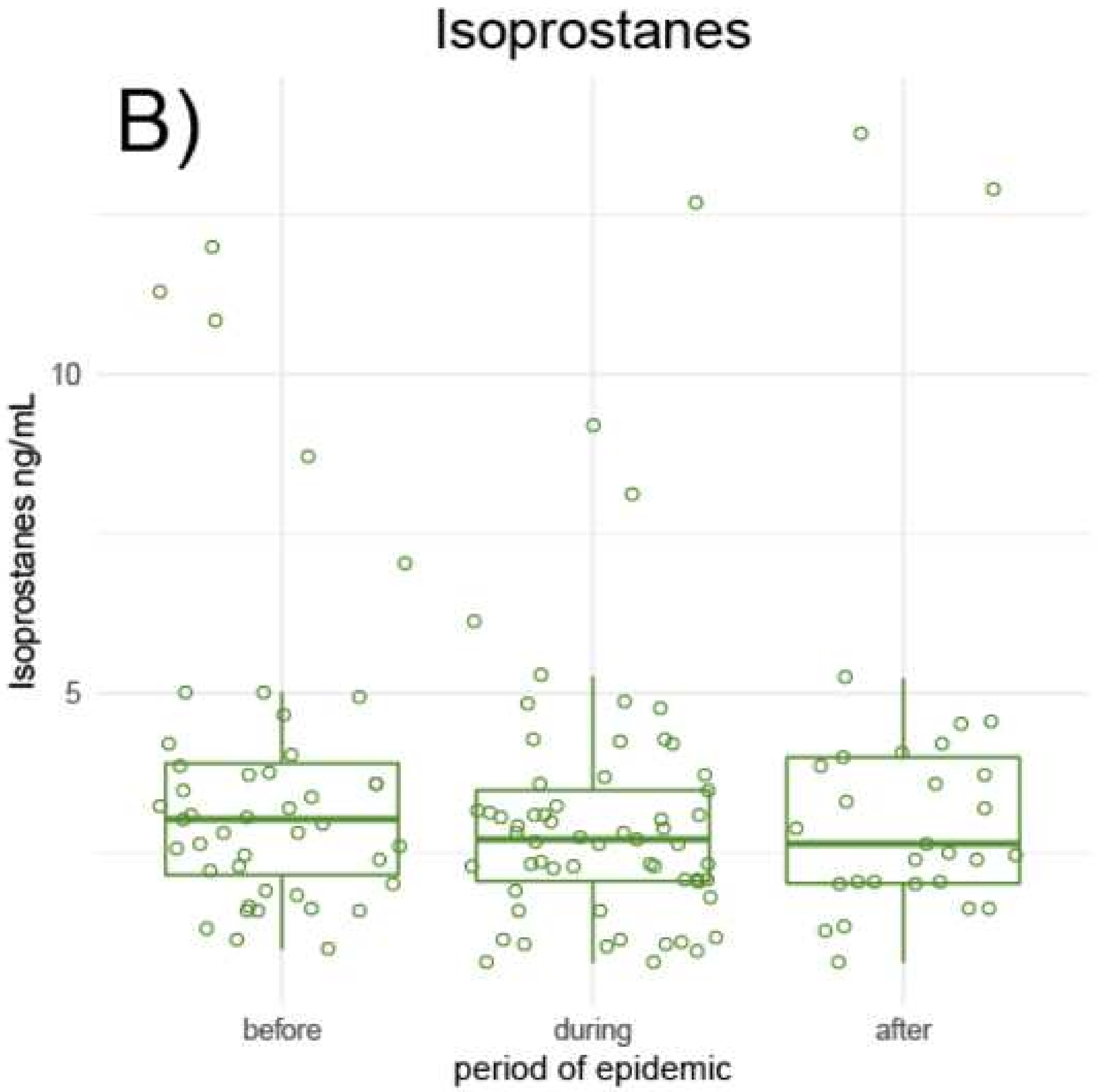

Click here to download Figure Fig2C1024_1.tif

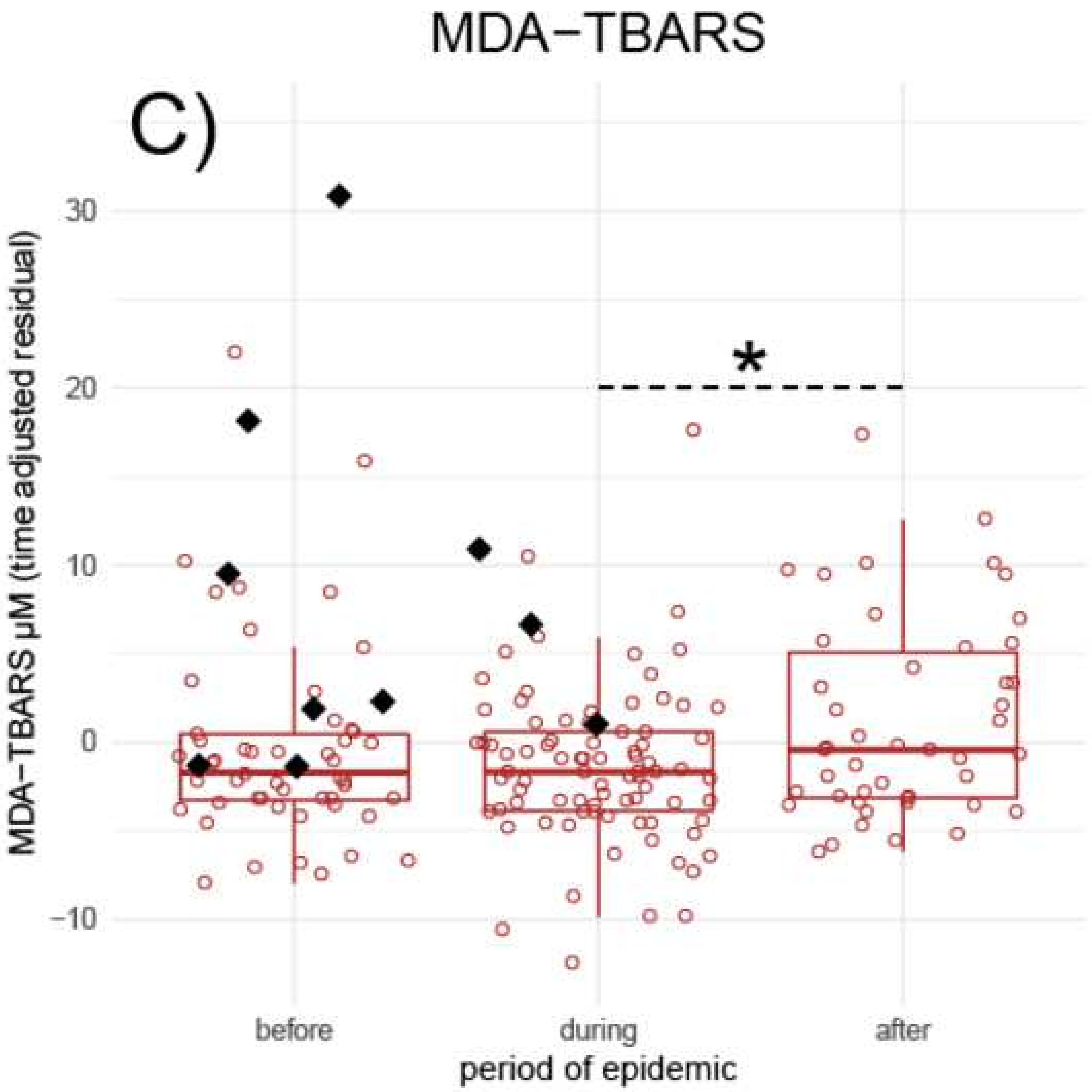

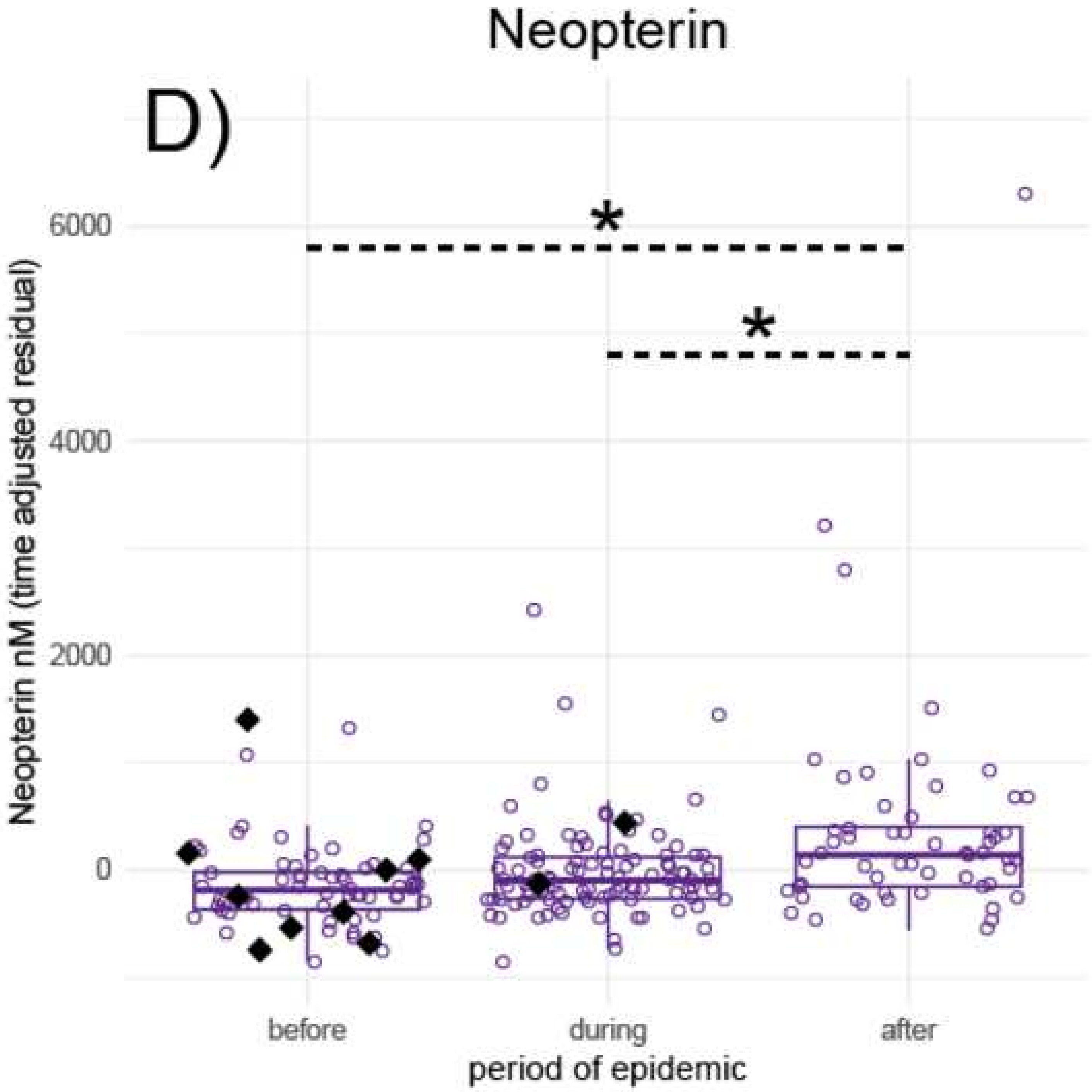
Neopterin
D)
*
*
6000
4000
2000
0
Neopterin nM (time adjusted residual)
before
during
after
period of epidemic

Click here to download Figure Fig2E1024_1.tif

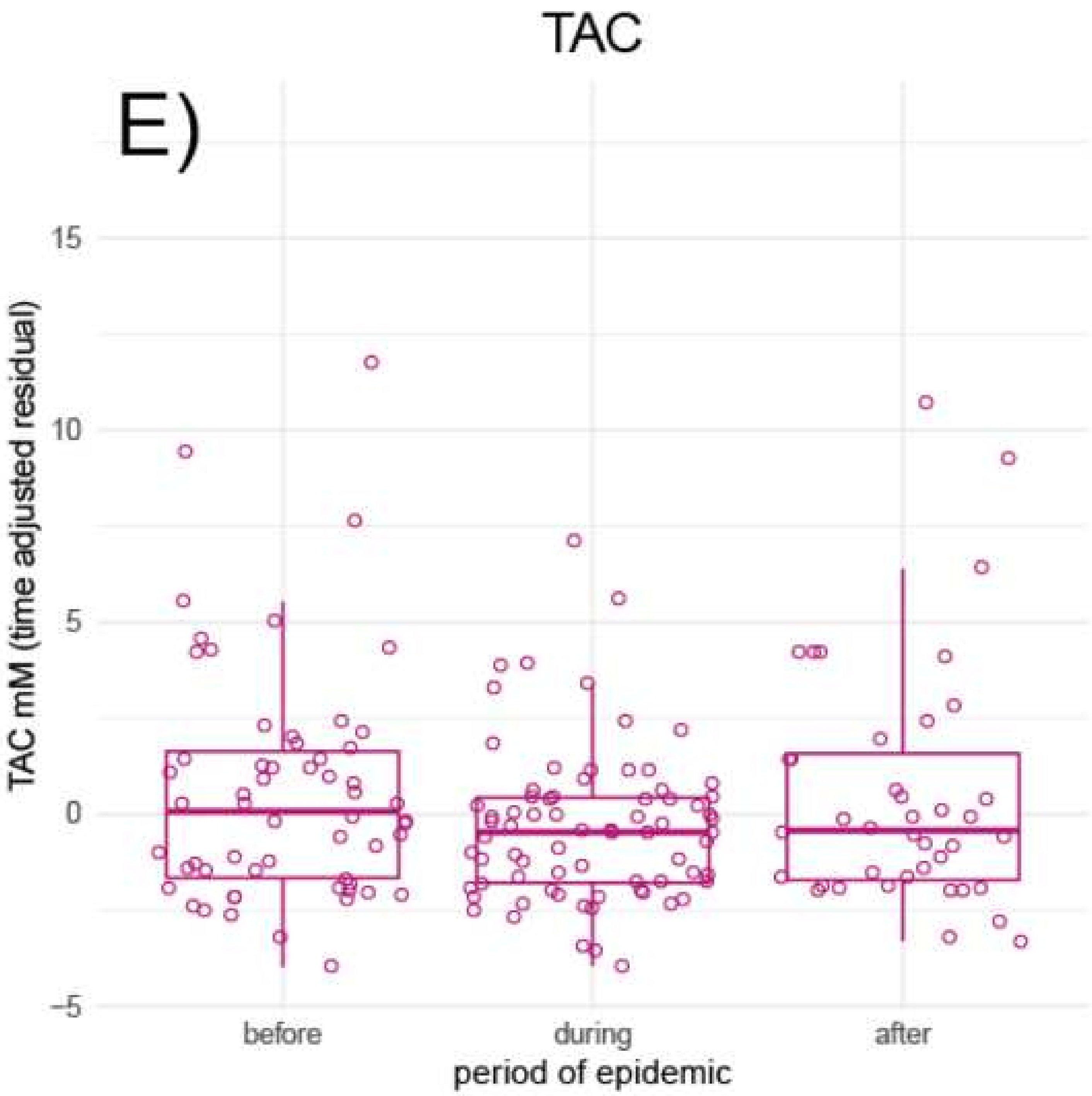

Click here to download Figure Fig3A1024_1.tif

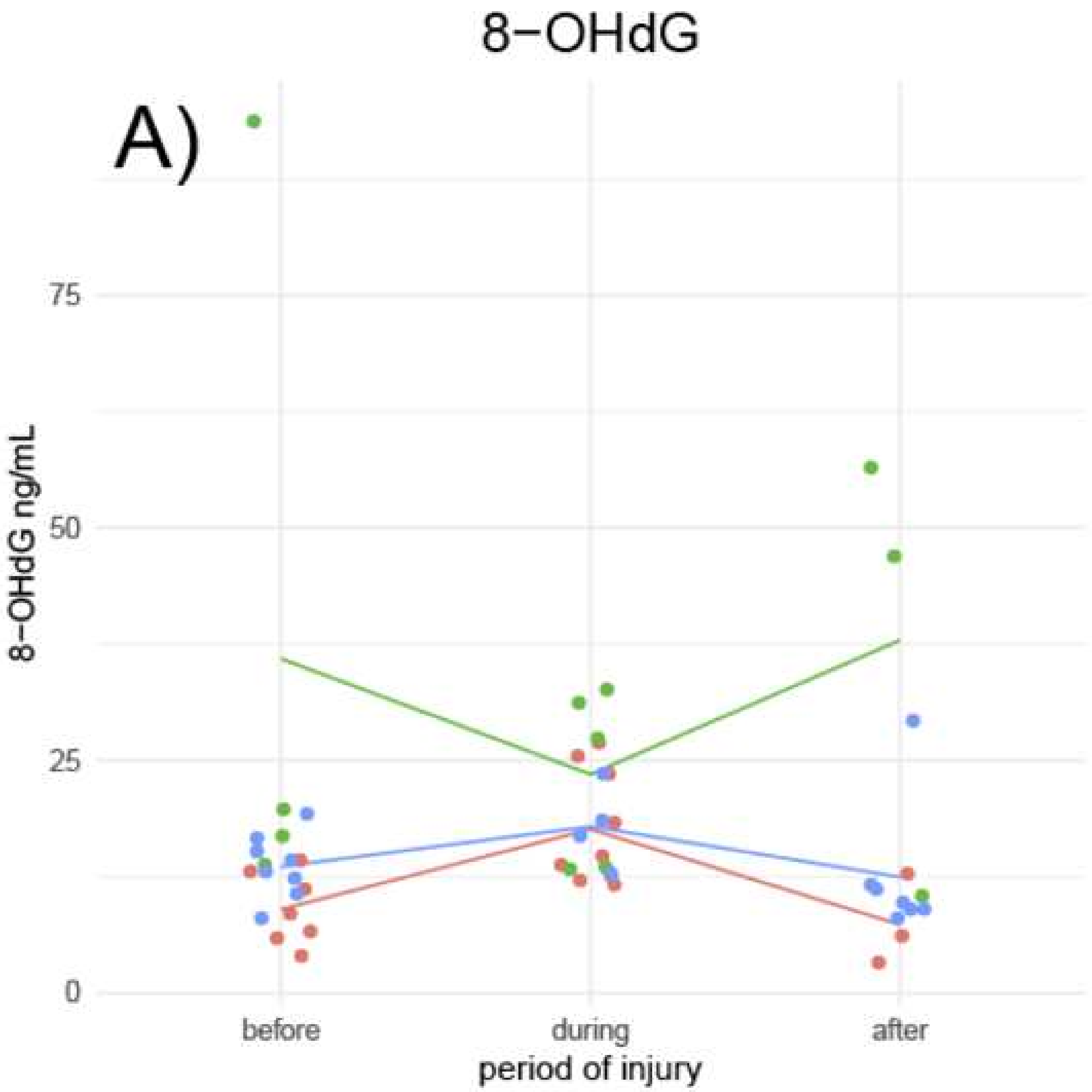

Click here to download Figure Fig3B1024_1.tif

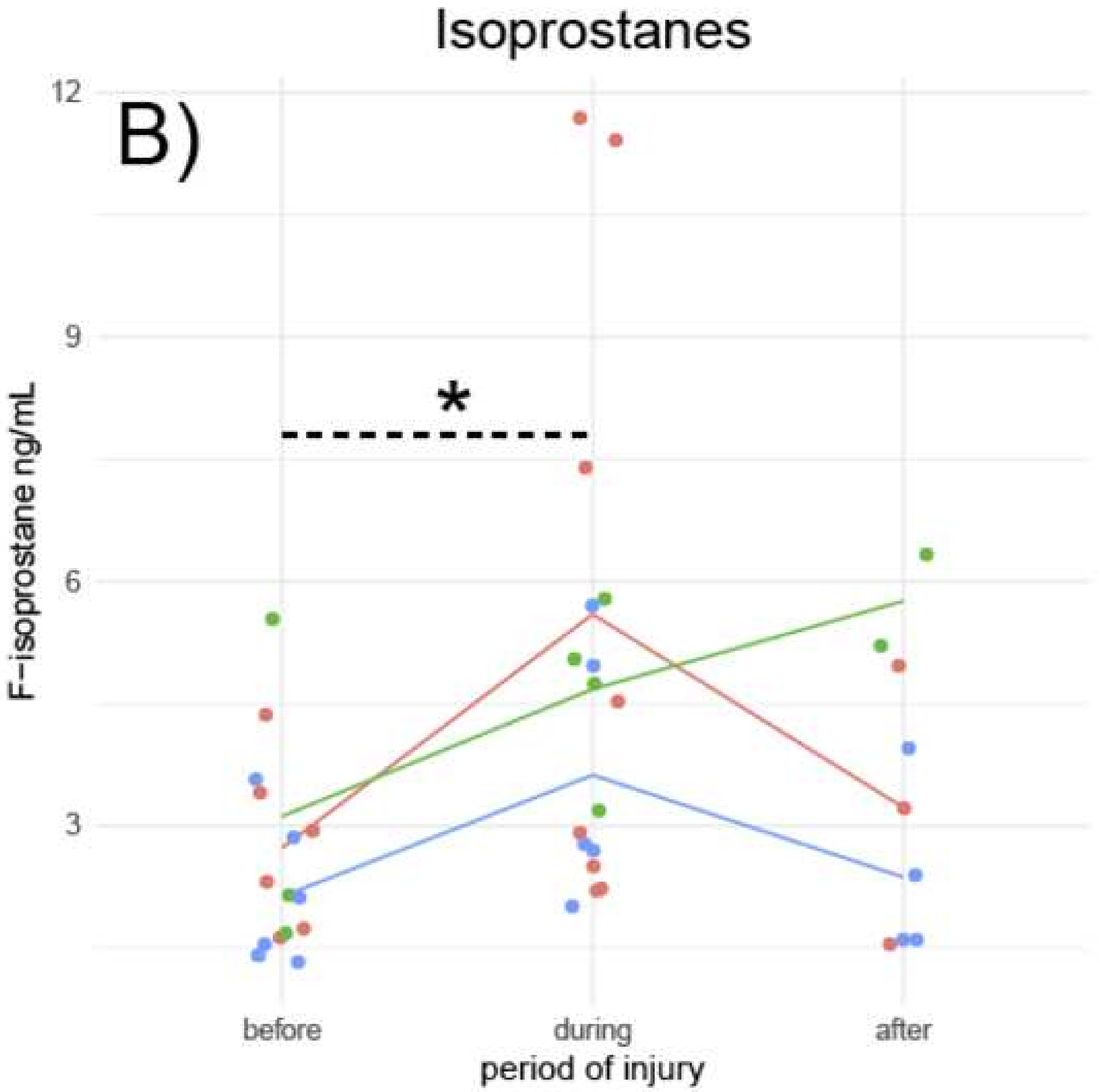

Click here to download Figure Fig3C1024_1.tif

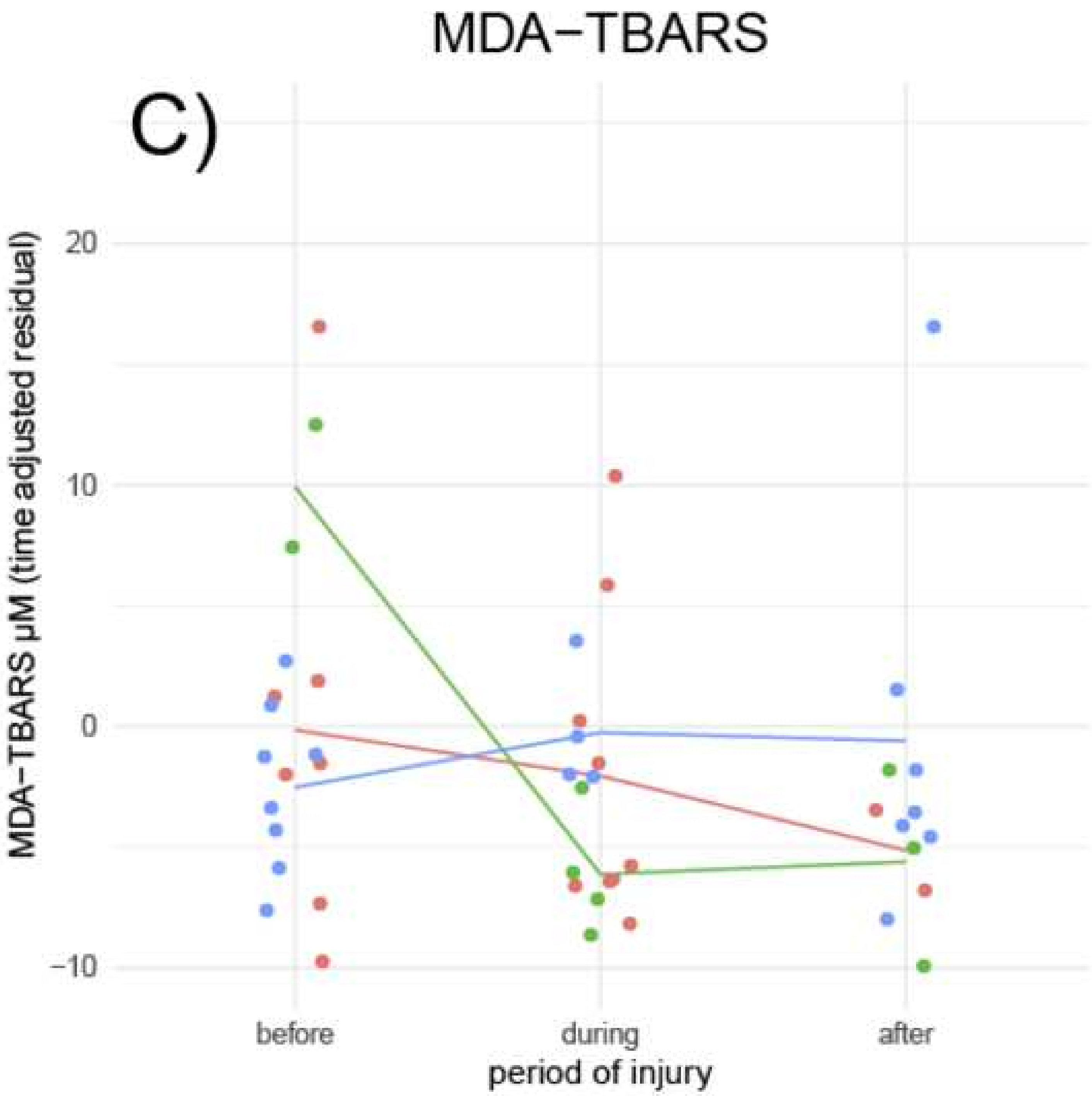

Click here to download Figure Fig3D1024_1.tif

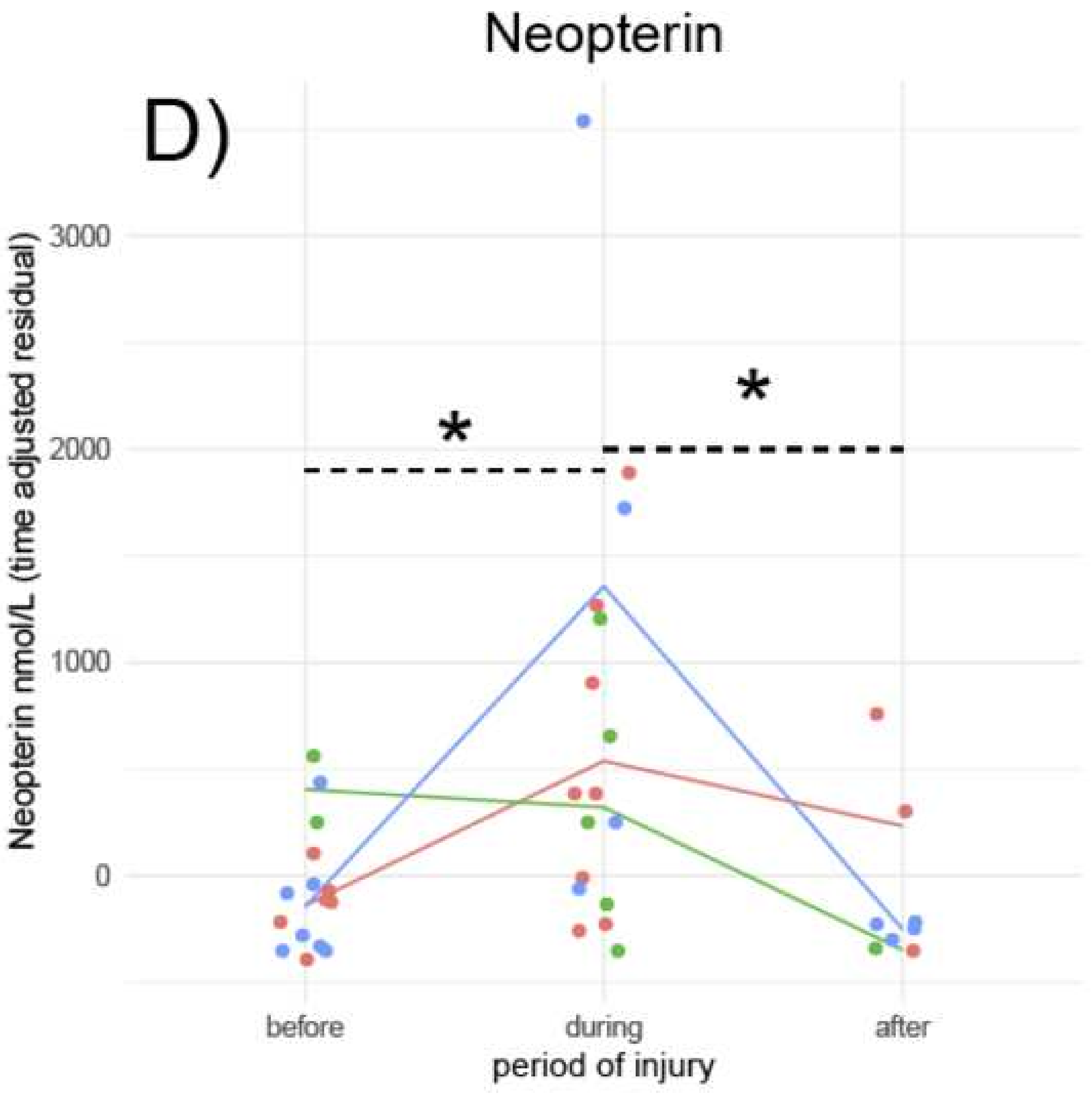

Click here to download Figure Fig3E1024_1.tif

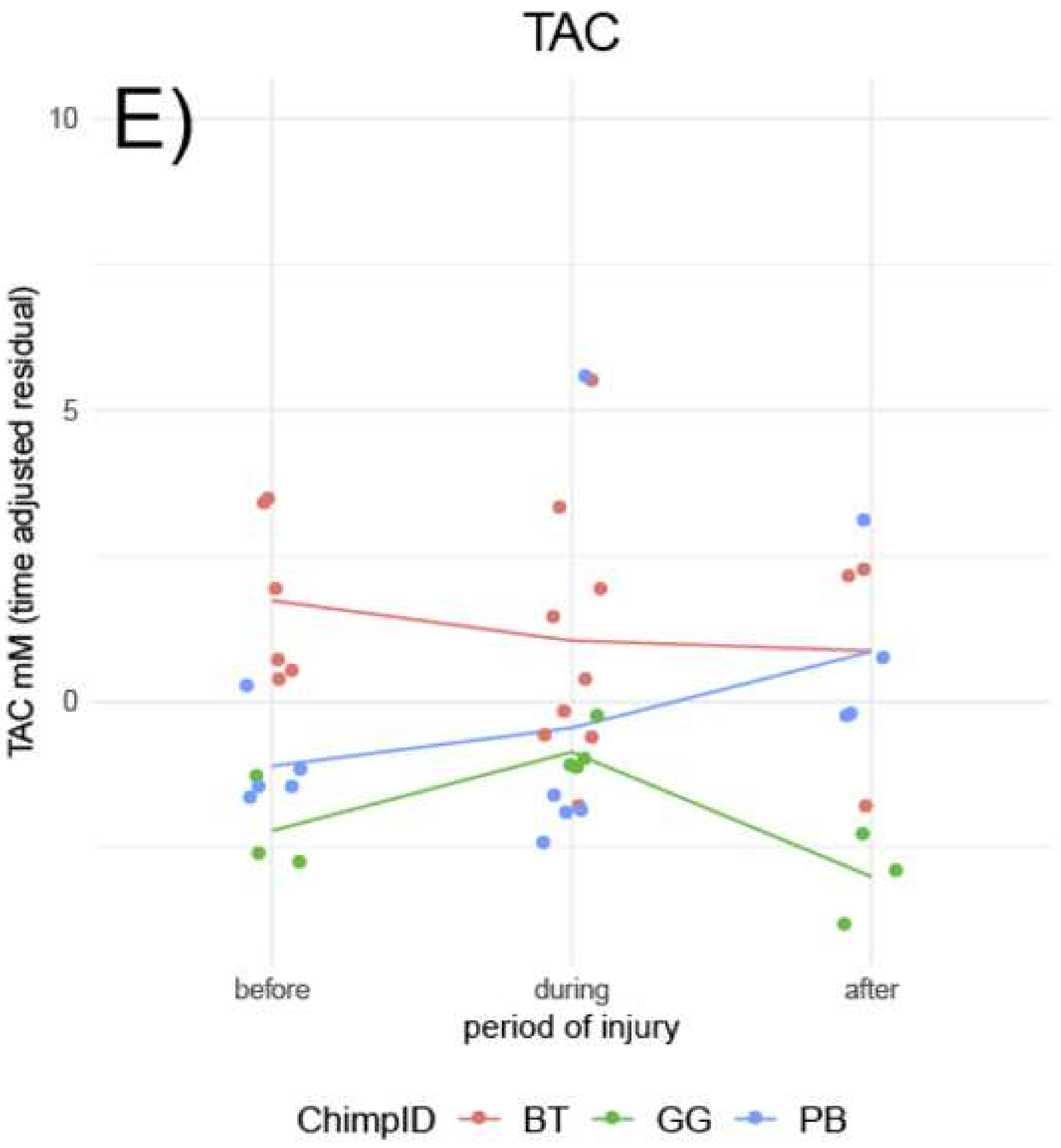

Click here to download Figure Fig41024_1.tif

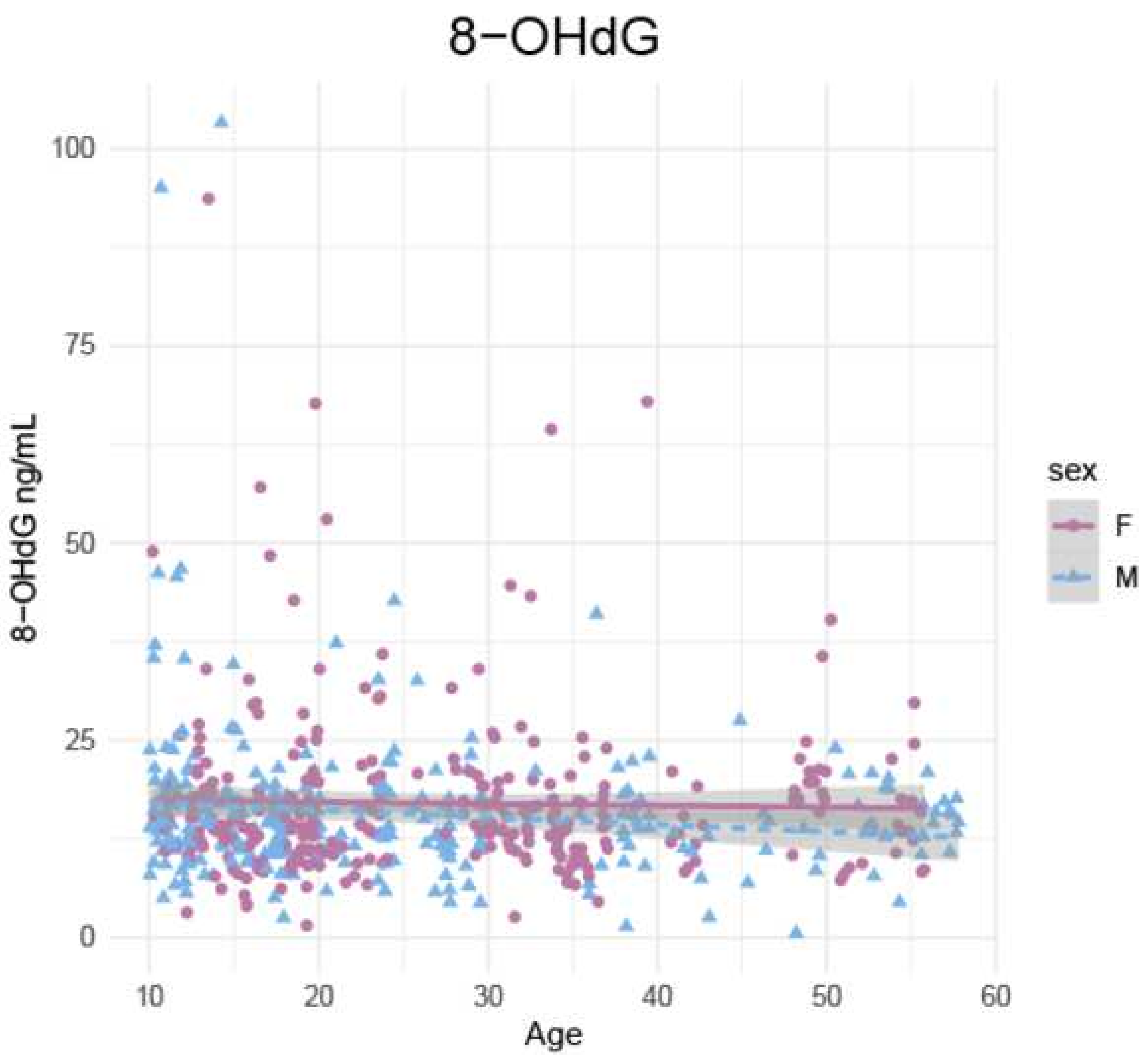

Click here to download Figure Fig5A1024_1.tif

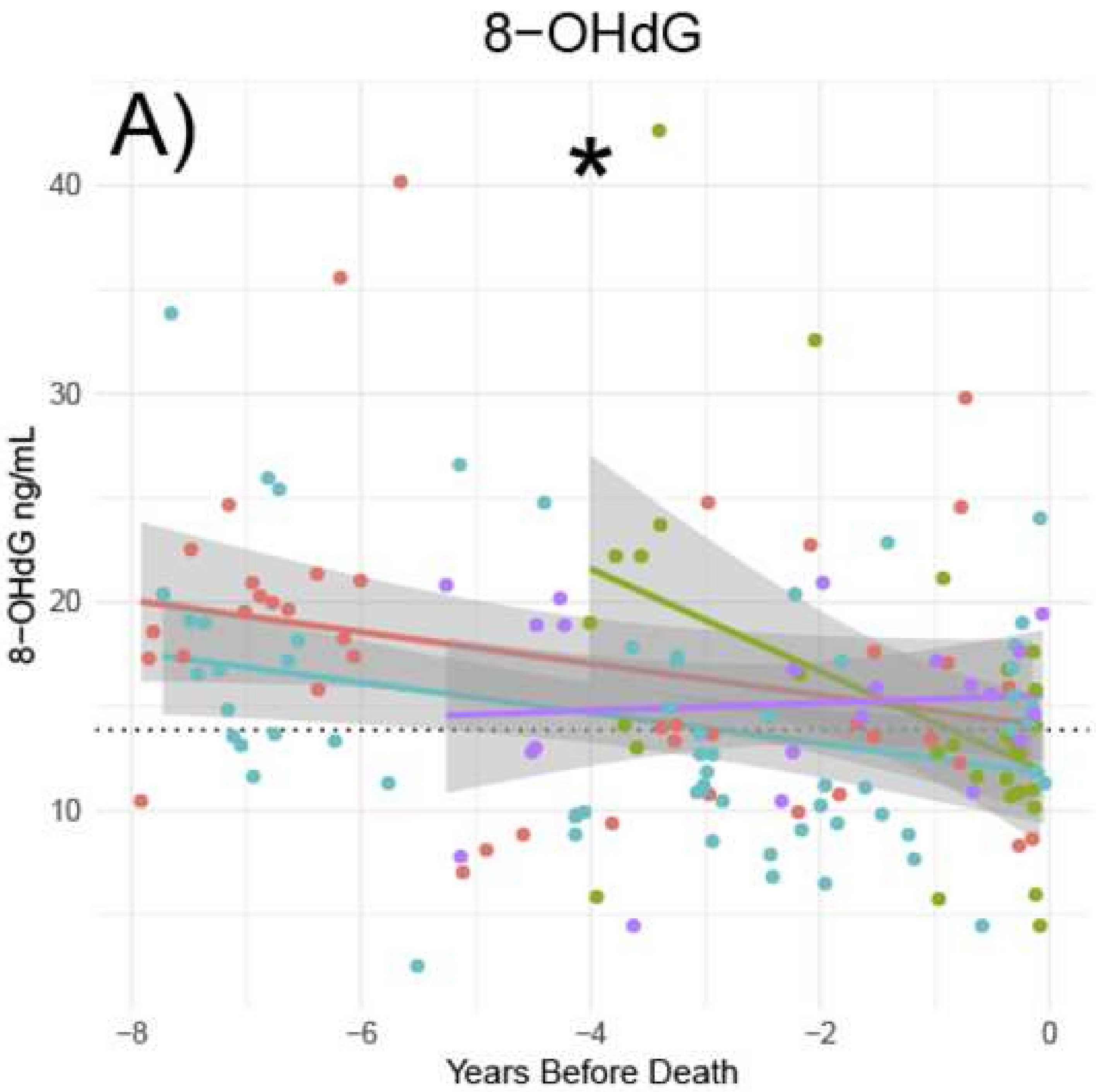

Click here to download Figure Fig5B1024_1.tif

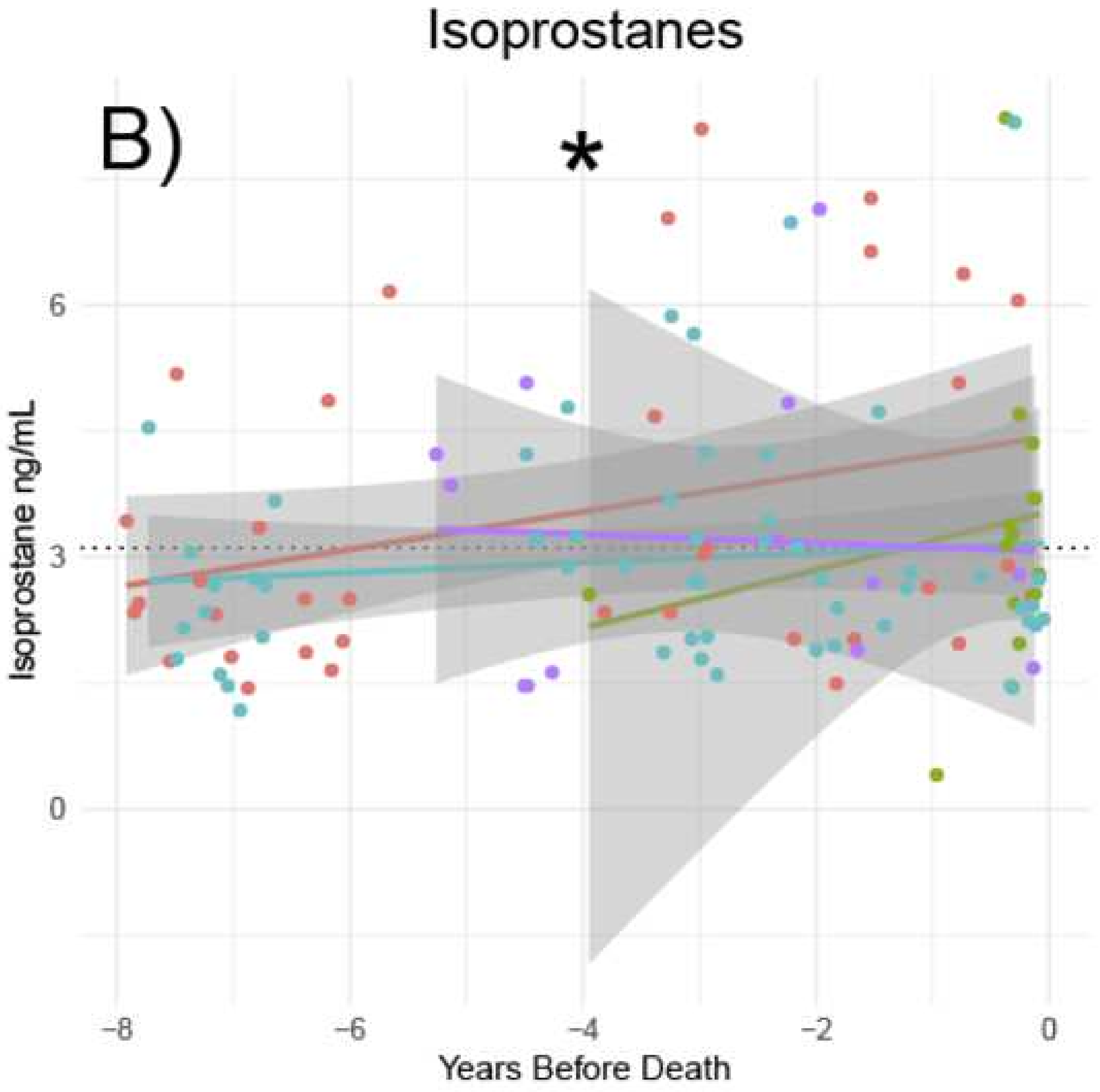

Click here to download Figure Fig5C1024_1.tif

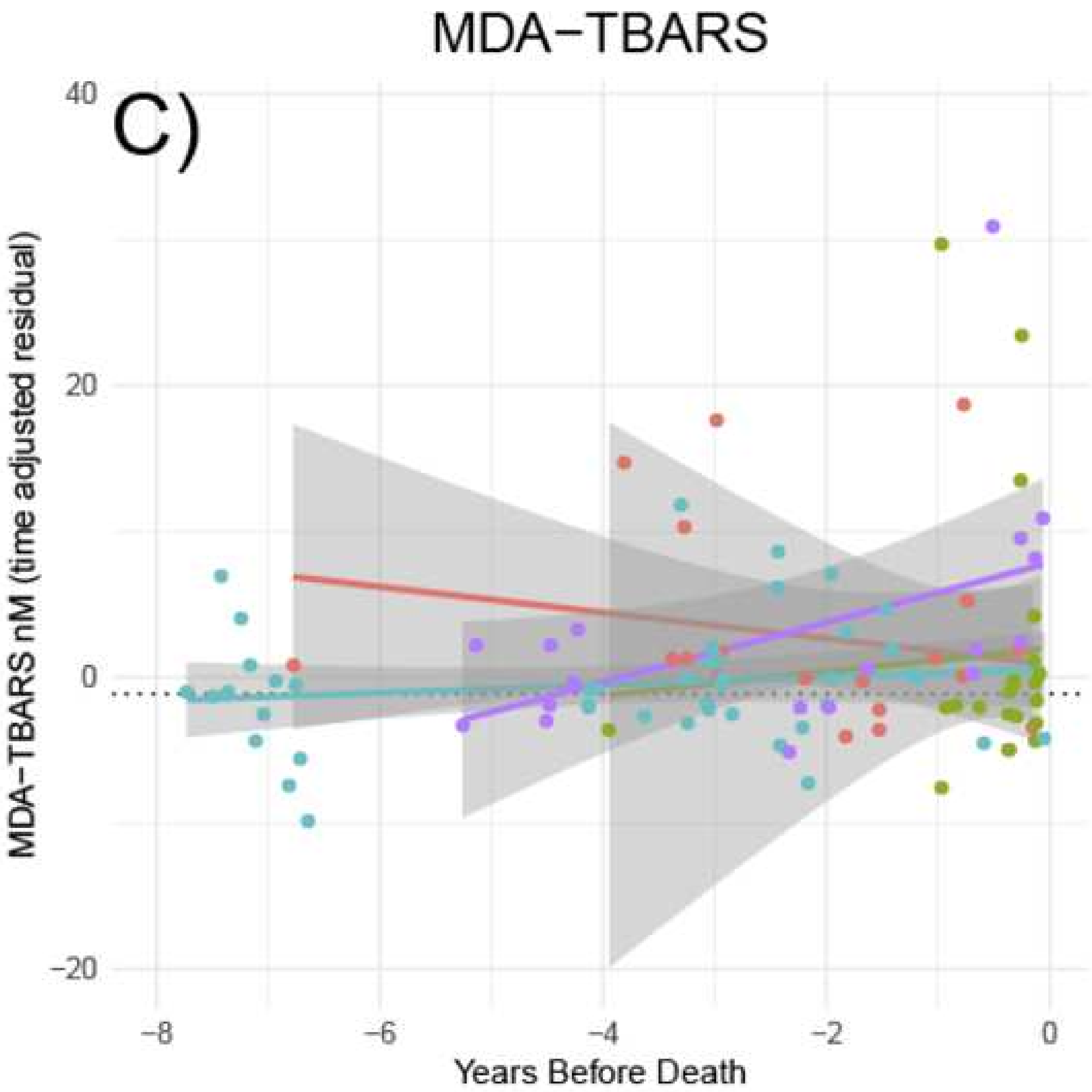

Click here to download Figure Fig5D1024_1.tif

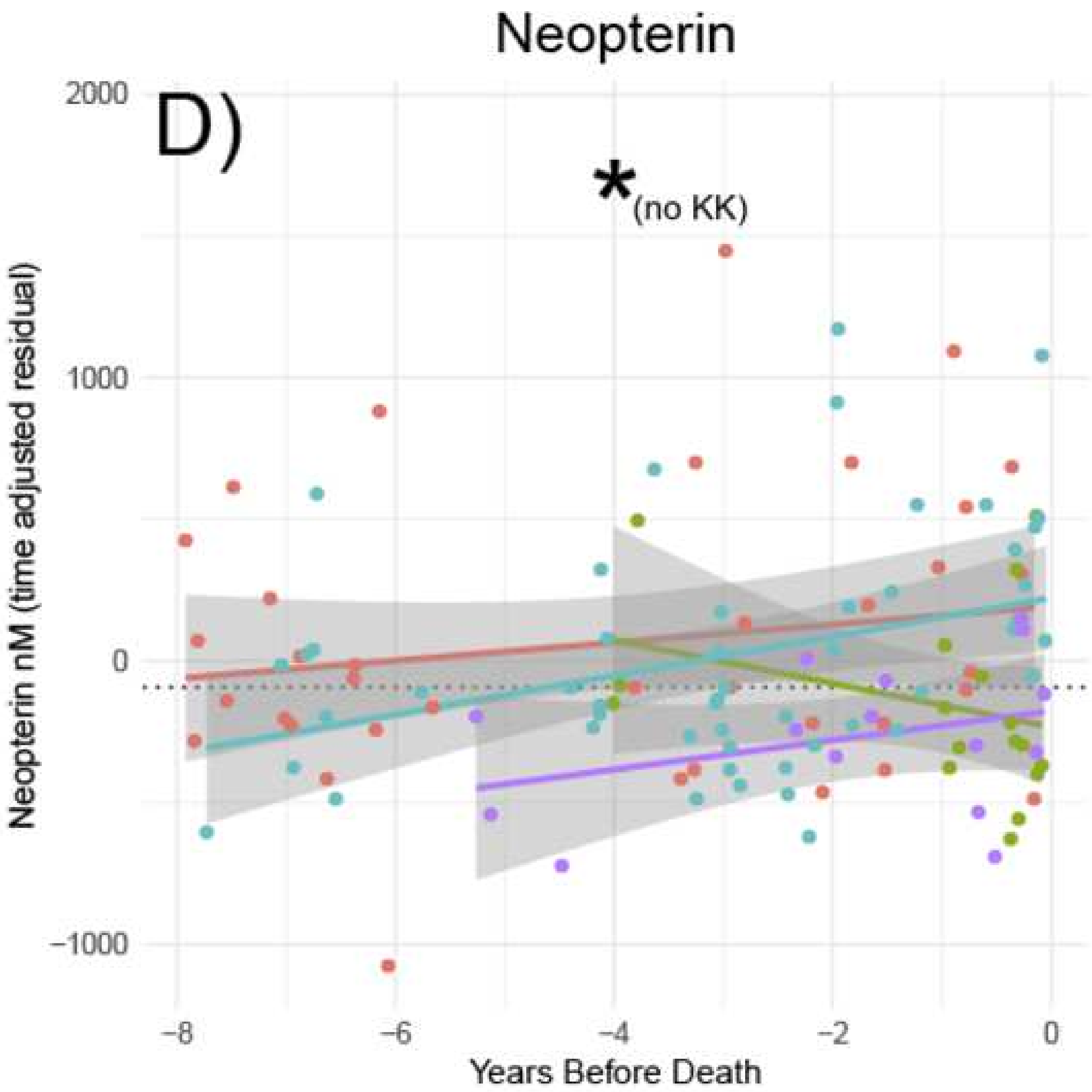

Click here to download Figure Fig5E1024_1.tif

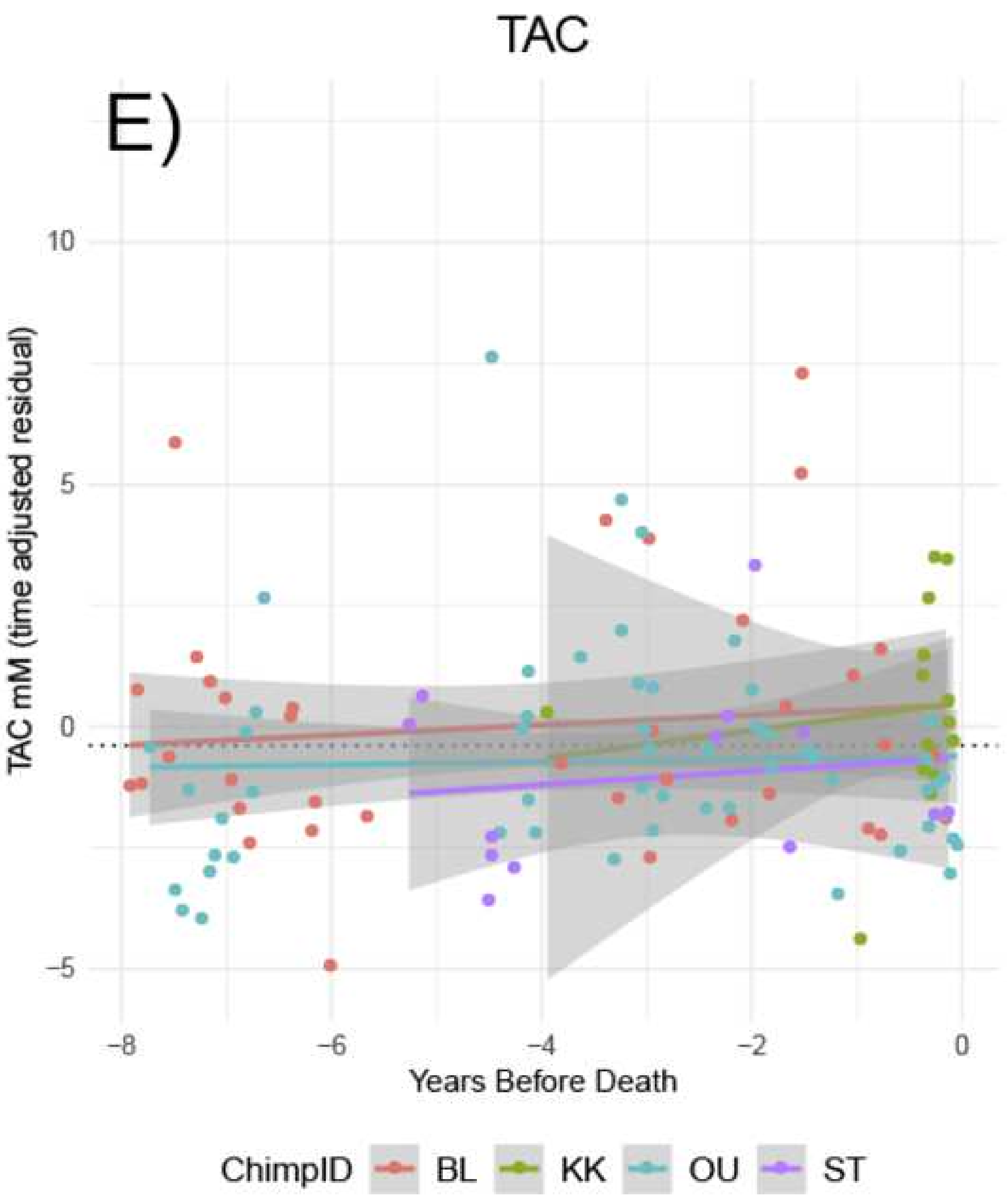

Click here to access/download
Supporting Information
OXS validation supplementary info to submit.docx
